# Insights on long-term ecosystem changes from stable isotopes in historical squid beaks

**DOI:** 10.1186/s12862-024-02274-7

**Published:** 2024-07-03

**Authors:** Alexey V. Golikov, José C. Xavier, Filipe R. Ceia, José P. Queirós, Paco Bustamante, Bram Couperus, Gaël Guillou, Anna M. Larionova, Rushan M. Sabirov, Christopher J. Somes, Henk-Jan Hoving

**Affiliations:** 1 GEOMAR Helmholtz Centre for Ocean Research Kiel, Kiel, Germany; 2 MARE—Marine and Environmental Sciences Centre/ARNET—Aquatic Research Network, Department of Life Sciences, University of Coimbra, Coimbra, Portugal; 3 British Antarctic Survey, Natural Environment Research Council, Cambridge, UK; 4 Littoral Environnement et Sociétés (LIENSs), UMR 7266, CNRS-La Rochelle Université, La Rochelle, France; 5 Wageningen Marine Research, Wageningen University and Research, IJmuiden, The Netherlands; 6 Department of Zoology, Kazan Federal University, Kazan, Russia

**Keywords:** Climate change, Food web, Arctic, North atlantic, Cephalopoda, Predator, Prey, Stable isotope analysis, Environmental conditions, Warming

## Abstract

**Background:**

Assessing the historical dynamics of key food web components is crucial to understand how climate change impacts the structure of Arctic marine ecosystems. Most retrospective stable isotopic studies to date assessed potential ecosystem shifts in the Arctic using vertebrate top predators and filter-feeding invertebrates as proxies. However, due to long life histories and specific ecologies, ecosystem shifts are not always detectable when using these taxa. Moreover, there are currently no retrospective stable isotopic studies on various other ecological and taxonomic groups of Arctic biota. To test whether climate-driven shifts in marine ecosystems are reflected in the ecology of short-living mesopredators, ontogenetic changes in stable isotope signatures in chitinous hard body structures were analysed in two abundant squids (*Gonatus fabricii* and *Todarodes sagittatus*) from the low latitude Arctic and adjacent waters, collected between 1844 and 2023.

**Results:**

We detected a temporal increase in diet and habitat-use generalism (= opportunistic choice rather than specialization), trophic position and niche width in *G*. *fabricii* from the low latitude Arctic waters. These shifts in trophic ecology matched with the Atlantification of the Arctic ecosystems, which includes increased generalization of food webs and higher primary production, and the influx of boreal species from the North Atlantic as a result of climate change. The Atlantification is especially marked since the late 1990s/early 2000s. The temporal patterns we found in *G*. *fabricii*’s trophic ecology were largely unreported in previous Arctic retrospective isotopic ecology studies. Accordingly, *T*. *sagittatus* that occur nowadays in the high latitude North Atlantic have a more generalist diet than in the XIX^th^ century.

**Conclusions:**

Our results suggest that abundant opportunistic mesopredators with short life cycles (such as squids) are good candidates for retrospective ecology studies in the marine ecosystems, and to identify ecosystem shifts driven by climate change. Enhanced generalization of Arctic food webs is reflected in increased diet generalism and niche width in squids, while increased abundance of boreal piscivorous fishes is reflected in squids’ increased trophic position. These findings support opportunism and adaptability in squids, which renders them as potential winners of short-term shifts in Arctic ecosystems.

**Supplementary Information:**

The online version contains supplementary material available at 10.1186/s12862-024-02274-7.

## Introduction

The Arctic is the Earth’s area currently most affected by climate change [1, 2]. Warming temperatures, melting sea-ice, reduction in snow cover, and changes in physical ocean dynamics are among the main changes observed to date [3, 4]. These changes impact all components of the Arctic marine food webs, including phyto- and zooplankton [5], sympagic algae and invertebrates [6], benthos [7], nekton [8, 9], fishes [10, 11], seabirds [12] and marine mammals [13, 14]. Monitoring of marine ecosystems’ structure and functioning and assessing changes on historical scale are among the priorities in Arctic science [15, 16]. However, logistical, financial and geopolitical challenges prevent the organization of a marine ecosystem monitoring program in the Pan-Arctic area [15, 16], hampering long-term observations and temporal analyses.

The North Atlantic is the main source of warm-water inflow to the Arctic [16]. Both modelled and observed mean annual temperatures in the Arctic continuously increase since the late 1990s/early 2000s [2, 17]. As a result, increasing environmental impact of warming in the Arctic has been especially noticeable since the late 1990s/early 2000s [2, 17]. Consequently, the Arctic marine ecosystems undergo an inflow of increasing numbers of Atlantic species occurring northwards [3–5, 7–11, 16, 18]. The influx of opportunistic, large, boreal fishes and invertebrates is leading to an increase of generalist species within the Arctic marine food webs (= ‘generalization’) and an increase in food web trophic complexity [3, 7, 10, 11, 18]. Furthermore, increased primary production [3, 19], as a result of temperature rise and sea-ice melting, drives an increase in abundance of newly arrived boreal species but is unfavorable for resident Arctic species [3, 7, 10, 11, 18]. Consequently, the Arctic marine ecosystems are becoming more similar to boreal Atlantic ecosystems over time (= ‘Atlantification’ sensu Ref [20]). where the pelagic food web component dominates over the benthic, and large fishes with generalist diets dominate over small fishes with specialist diet [3, 7, 10, 11, 18]. A major obstacle for understanding the ongoing changes in the contemporary Arctic Ocean is the lack of baseline retrospective data.

Stable isotope analysis (SIA) can potentially be used to back-track previous ecosystem state, and to obtain an insight of current changes [21, 22]. The most commonly applied stable isotopes in marine ecological studies are carbon *δ*^13^C, which is mainly used to assess the primary source of dietary carbon, and nitrogen *δ*^15^N, which is typically used to assess the trophic position (TP) [23, 24] (see Methods for *δ* definition). Isotopic values at the base of the food web (= baseline values) are needed to be taken into account to correctly interpret the meaning of isotopic values higher up the food web [23, 24]. Both *δ*^13^C and *δ*^15^N baseline values are highly variable in space and time [25–27]. For instance, baseline values and respectively bulk SIA values of detritivores and carnivores can differ significantly in close proximity to each other along the western Pacific coast [26, 28]. Without accounting for baseline differences, TPs of populations with similar diets would erroneously be estimated as different among these locations, while in fact their TPs are similar [28]. Additionally, long-term bulk SIA *δ*^13^C data need to be corrected for the Suess effect, which is the anthropogenic-induced decline in baseline *δ*^13^C values [29].

Metabolically inert but continuously growing tissues record stable isotope signatures during their formation, reflecting and preserving information on animals ecology through time without altering these signatures afterwards [30] (= archival tissues). The archival tissues with known growth patterns can be used to reconstruct individual life-long trophic ecology by analysing the SI composition per growth layer [31–38]. This approach indirectly provides insights into the ecosystem structure back in time [31–38]. In the Arctic and North Atlantic, retrospective ecological analyses were done for coral endoskeletons, bivalve shells and marine mammal teeth and tusks [31–38]. However, all of these are long-living taxa, in which it can be difficult to disentangle ontogenetic signals from changes in the ecosystems, since the respective isotopic signal changes over time [33, 35]. In general, most of the long-term bulk SIA studies of marine mammals from the Arctic and North Atlantic published to date do not account for the Suess effect and baseline variation [22, 31, 34, 37, 39–41]. The use of compound-specific SIA (CSIA) does not require baseline corrections, since it uses differences in isotopic signatures of trophic and source amino acids to overcome baseline-related issues [42]. However, challenges of this method include unknown taxon-specific trophic discrimination factor and *β* values for many taxa, as well as the requirement for additional data collection for fingerprinting of *δ*^13^C and *δ*^15^N [42]. Overall, both bulk and CSIA retrospective studies on Arctic and North Atlantic marine mammals present either that *δ*^13^C and *δ*^15^N increase, decrease or stay the same of over time in the different areas and across species [22, 31, 34, 36, 37, 39–41, 43, 44] (Tables S1 & S2). This suggests that top predators’ trophic ecologies respond to ecosystem changes in diverse and inconsistent ways. Few studies have detected changes in the trophic ecology of Arctic top predators since the late 1990s/early 2000s [34, 39], which is the onset of increased environmental effects of climate change in the Arctic [2, 17]. Top predators are positioned the furthest away from the basal part of the food web, which may limit their direct response to certain ecosystem changes, potentially explaining the absence of clear retrospective stable isotopic patterns in relation to environmental change.

Retrospective SIA studies of abundant filter-feeding, long-living, sedentary, and slow moving invertebrates (= corals and bivalves, respectively) in the Arctic and adjacent parts of the North Atlantic largely focus on *δ*^15^N [32, 33, 35, 38]. Ontogenetic patterns were found in bivalves [35, 38], and temporal changes in baseline values were evident through both corals and bivalves [32, 33, 35, 38]. Filter feeders occupy the position directly above the basal part of the food web, which probably explains them being able to show temporal baseline changes. While the Arctic was subjected to major ecosystems changes [3–5, 7–11, 16, 18] since the late 1990s/early 2000s [2, 17], these changes were not reflected in the retrospective SIA studies of filter feeders [32, 35, 38]. Their proximity to the basal part of the food web is most likely the explanation. Longevity of over a hundred of years in a single bivalve shell or coral endoskeleton piece, coupled with low sample sizes, may limit the possibility to disentangle ontogenetic and temporal SIA patterns [32, 33, 35, 38]. In this sense, abundant mesopredators (= animals from mid-trophic levels) with short life cycles can be good candidates for retrospective studies. Changes in their relatively short life histories over time can provide additional parameters to compare among different years, offering a higher resolution view compared to long-living taxa. One such taxon of mesopredators is the Class of Cephalopoda.

Cephalopods (Phylum Mollusca) are abundant and important as prey and predators in the Arctic [9, 45]. They prey on crustaceans, cephalopods and fishes, and their main predators are fishes, sharks, seabirds and marine mammals [9, 46]. Cephalopods can be affected by climate change leading to shifts in their geographical distribution, potential habitat expansions, and increases in biomass [9, 28, 47, 48]. The relatively short lifespan, fast population turnover, short generation time and high biomass production [49] supposedly explain high adaptability of this group to a changing environment [9, 28, 47, 49]. As such, the ongoing changes in the Arctic environment and food webs (outlined above) [3, 7, 10, 11, 18] will most likely be reflected in the trophic ecology of cephalopods. And these changes in trophic ecology can be retrospectively tracked by SIA, as seen from other groups with suitable archival tissues [31–38]. Cephalopods have chitinous jaws (= beak) to cut their prey into smaller pieces [50, 51]. The beaks were successfully used as archival tissue for detailed SIA life history reconstructions of contemporary cephalopods in the Arctic and Antarctic [52–54]. However, there is only a single study from the Atlantic that involves historical squid, where the sample size (*n* = 4) is too small to assess potential ecosystem implications [55].

Our main study species is the Boreoatlantic armhook squid *Gonatus fabricii* (Lichtenstein, 1818). It is the most abundant cephalopod in the Arctic Ocean (standing biomass up to 8 million tonnes in the Nordic Seas alone), and is important as both prey and predator [9, 56, 57]. *Gonatus fabricii* has an ontogenetic descent from epi- to bathypelagic layers, and performs diel vertical migrations [58, 59]. Our second study species from the North Atlantic is the European flying squid *Todarodes sagittatus* (Lamarck, 1798), which is an abundant, ecologically relevant and commercially important squid in the North Atlantic [60, 61]. *Todarodes sagittatus* performs diel vertical and large scale seasonal horizontal feeding migrations, but no has ontogenetic descent [60, 61]. It was chosen as an abundant species of North Atlantic squid fauna, which occasionally enters low latitude Arctic ecosystems [60, 61]. Both species show ontogenetic dietary shifts from small to large crustaceans, and then to fishes and cephalopods, including cannibalism [54, 58, 60–62]. Early paralarvae of *T. sagittatus* are detritivorous [63].

We hypothesize that shifts in squids’ ecology in low latitude Arctic marine ecosystems will coincide with the climate-driven ecosystem changes in the area, especially notable since the late 1990s/early 2000s. Specifically, we expect that (a) increased biodiversity in and generalization of food webs, and high abundance of newly arrived boreal species will alter the diet and niche width of squid; and (b) high abundance of large, piscivorous, boreal fishes will result in changes of TP in squid. In order to test these hypotheses, we use *δ*^13^C and *δ*^15^N signatures in *G. fabricii* from the low latitude Arctic areas (the Baffin Bay and southern part of Nordic Seas), which were sampled between 1900 and 2019. *Todarodes sagittatus* from the high boreal Atlantic (Iceland and Faroe Islands) was sampled between 1844 and 2023. The data were corrected for baseline isoscapes from a global ocean biogeochemical model (see Methods) [26, 27, 64, 65] to adjust for the Suess effect and baseline variation. Our study provides evidence that archival tissues in an abundant and accessible taxon of short-living mesopredators can be used for retrospective isotopic ecology studies in the Arctic and beyond, offering insights into major ecosystem shifts.

## Methods

### Material used, measurements and squid size estimations

**Fig. 1:**
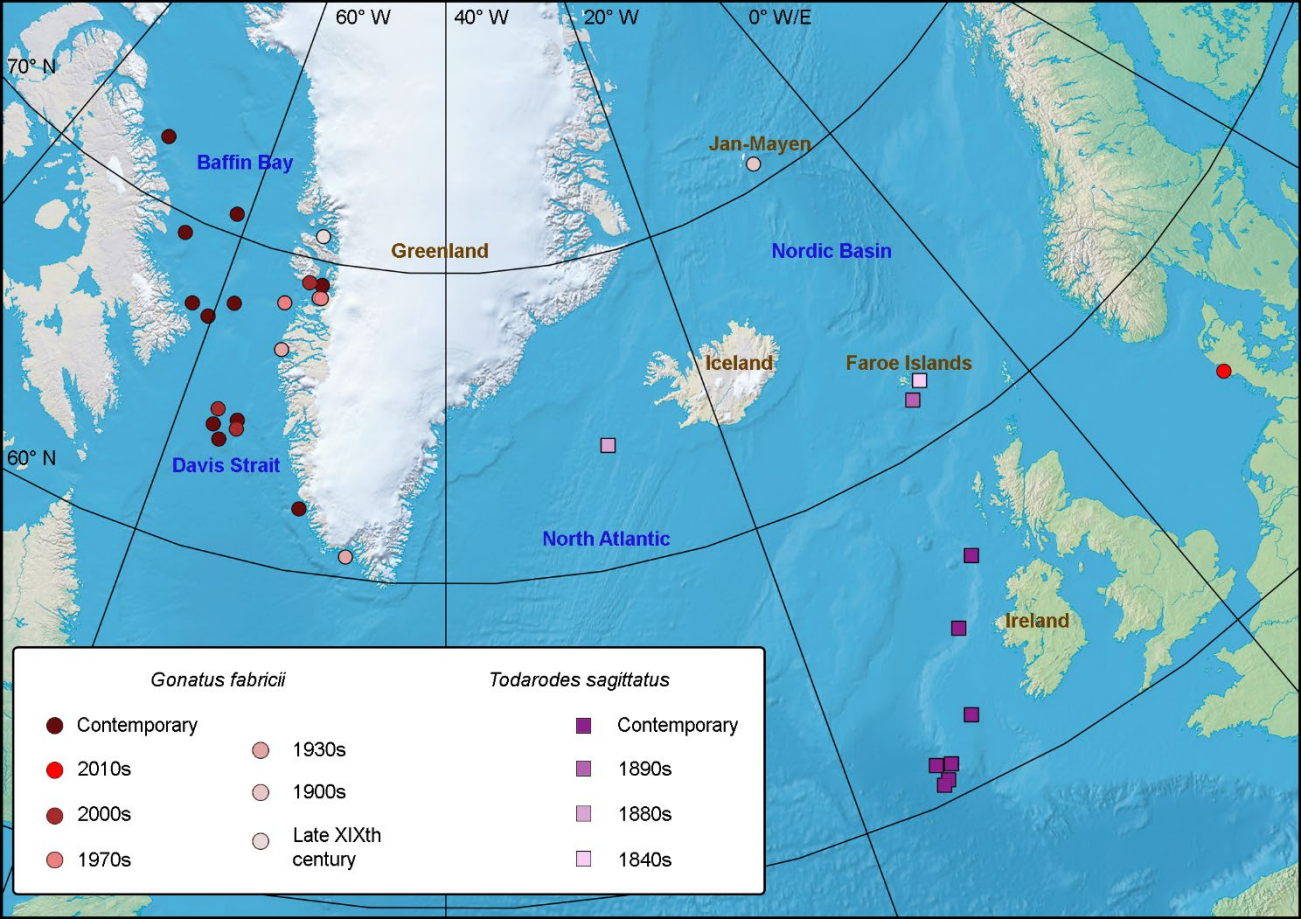
Sampling locations and time series of *Gonatus fabricii* and *Todarodes sagittatus*. Contemporary time series represents 2016, 2017 and 2019 in *G. fabricii* (reused from Golikov et al. 2022 [54]) and 2016–2018 and 2023 in *T. sagittatus*; rationale behind the pooling is provided in Methods. Geographic objects used in the text are marked on the map

Nineteen *G. fabricii* beaks from the Nordic Seas and 28 beaks from the Baffin Bay and Davis Strait were analysed (Fig. 1; Table 1). Main time series are ‘1900s’, ‘1930s’, ‘1970s’, ‘2000s’ and ‘2010s’, while ‘late XIX^th^ century’ with *n* = 2 were only used in some analyses (see below). Stable isotope data on ‘contemporary’ *G. fabricii* beaks, sampled in 2016, 2017 and 2019 in the Baffin Bay and Davis Strait were previously analysed in Ref [54]. We did not pool these beaks with the ‘2010s’ from this study (sampled in 2014) because the ‘2010s’ were from the Nordic Seas and *δ*^13^C values in *G. fabricii* were significantly different between the Nordic Seas and Baffin Bay [57]. Thirty one *T. sagittatus* beaks, forming the time series ‘1840s’, ‘1880s’, ‘1890s’ and ‘contemporary’ from Faroe Islands, Iceland and Ireland were analysed (Fig. 1; Table 1). The ‘contemporary’ *T. sagittatus* beaks were sampled in 2016–2018 and 2023 in Ireland. The majority of the samples were borrowed from Natural History Museum of Denmark (Copenhagen, Denmark). The only exceptions were: ‘2000s’ time series of *G. fabricii* were from GEOMAR (Kiel, Germany); and ‘contemporary’ *T. sagittatus* were caught as a bycatch during blue whitening stock assessment surveys by R/V ‘Tridens’, Wageningen Marine Research (IJmuiden, The Netherlands). Mantle length (ML in mm), mass (W in g), sample size, year and sampling method are provided in Table 1. Specimens obtained from the stomach contents of predators (*n* = 45) were allocated within a uncertainty radius around a predator’s suggested capture or stranding location, coinciding with the baseline model grid (see model description below) [26, 27, 64].

**Table 1:** Samples of *Gonatus fabricii* and *Todarodes sagittatus* used in this study. ML – mantle length, W – mass, *n* – sample size

*Gonatus fabricii*							
Time series	Year	Area	Sampling: trawl/predator	ML, mm (min–max)^1^	W, g (min–max)^1^	*n*, individuals	*n*, subsections per individual (min–max)
Late XIX^th^ century^2^	1882	Baffin Bay	Trawl	285.0	396.6	1	17
	1891			230.0	144.6	1	15
1900s	1900	Jan-Mayen (Nordic Seas)	Northern fulmar (*Fulmarus glacialis*)	149.7–197.8	72.3–146.7	9	8–10
1930s	1930	Davis Strait	Greenland shark (*Somniosus microcephalus*)	215.6, 217.8	181.7, 186.3	2	18
	1936	Baffin Bay	Shrimp trawl	170.0–189.0	83.2–98.0	5	11–12
1970s	1975	Baffin Bay		186.0–223.0	70.3–146.1	5	12
	1977			154.0, 182.0	66.3, 83.9	2	12
	1979			136.9, 172.0	58.9, 103.8	2	10
2000s	2004		‘Alfredo 3’ trawl	126.0–214.0	48.0–178.3	10	11–14
2010s	2014	Nordic Seas^3^	Sperm whale (*Physeter microcephalus*)	180.3–218.2	116.7–187.1	10	15–17
Contemporary^4^	2016	Baffin Bay, Davis Strait (both areas each year)	‘Alfredo 3’ trawl	238.0–325.0	218.0–294.0	5	13–17
	2017			214.0–285.0	116.5–380.0	6	11–16
	2019			230.0, 252.0	165.5, 255.7	2	14, 16
***Todarodes sagittatus***							
1840s	1844	Faroe Islands	Long-finned pilot whale (*Globicephala melas*)	182.5–240.5	125.2–287.4	4	12–15
1880s	1881	Iceland		177.5–249.4	115.1–320.5	10	12–15
1890s	1897	Faroe Islands		250.4–285.1	324.2–379.0	10	15–20
Contemporary	2016	Ireland	Pelagic trawl	256.0	357.0	1	15
	2017			343.0	1051.0	1	21
	2018			213.0	180.0	1	12
	2023			209.0–405.0	188.0–1844.0	4	14–23

^1^estimated for beaks found in predators’ stomach contents, measured in trawl-caught individuals (see Methods for references to equations used for estimations);

^2^not included to the majority of analyses due to *n* = 2;

^3^found in the sperm whale stranded in Denmark, which are known to prey on large *G. fabricii* in the Nordic Seas [56];

^4^published in Golikov et al. 2022 [54] and reused in this study

Upper beaks were used for SIA, in line with previous ontogenetic SIA reconstructions for cephalopods [52, 54, 55]. Upper beak rostrum length (URL) and crest length (UCL) were measured following Ref [50]. Predator stomach contents-provided beaks’ measurements were used to estimate ML of the respective individuals. ML was estimated from URL, and ML at given subsection of the crest was estimated from UCL. W at given subsection was estimated from ML, as no reliable equation exist to estimate W directly from UCL. The equations are from Refs [54, 57]. for *G. fabricii* and Refs [62, 66]. for *T. sagittatus*.

Consecutive subsections of the upper beak from rostrum to the end of the crest were prepared following Ref [54]. (Fig. S1). Subsections in the anterior half of the beak sized as close as possible to 1 mm, and subsections in the posterior half of the beak sized as close as possible to 2 mm [54] (Fig. S1). First eight 1 mm subsections are common to all studied individuals, corresponding to *G. fabricii* ML 7–65 mm and *T. sagittatus* ML 9–81 mm. In the smallest beaks of *G. fabricii*, only these eight subsections were available to prepare, or in some cases, 1–3 of the posterior subsections were also prepared. In the largest beaks of *T. sagittatus*, the posterior subsections were larger than 2 mm. The largest beaks belong to the ‘contemporary’ time series. Transparent parts of the upper beaks were cut prior to subsections’ preparation, as they have significantly different isotopic values from the tanned parts of the beaks [67]. Transparent parts darken in predators’ stomach contents [51]. If the posterior-most part of the crest was not already broken out in the stomach, the posterior-most 3 to 4 mm were discarded. A total of 579 *G. fabricii* subsections and 474 *T. sagittatus* subsections were analysed.

### Stable isotope analyses

All beak subsections were dried at 60 °C for 24–48 h, weighed (to the closest value to 0.3 mg) with a micro-balance and sterile-packed in tin containers. Stable isotopic signatures were determined by a continuous flow mass spectrometer (Delta V Plus with a Conflo IV Interface, Thermo Scientific, Bremen, Germany) coupled to an elemental analyzer (Flash 2000 or EA Isolink, Thermo Scientific, Milan, Italy) and expressed in ‰ as:

$${\delta ^{13}}{\text{C}}{\text{ }}{\text{and}}\,{\delta ^{15}}{\text{N}}{\text{ }} = {\text{ }}\left[ {\left( {{{\text{R}}_{\text{sample}}}/{{\text{R}}_{\text{standard}}}} \right)\, - \,1} \right]{\text{ }}*{\text{ }}1000$$

where R = ^13^C/^12^C and ^15^N/^14^N, respectively. The carbon and nitrogen isotope ratios were expressed in delta (*δ*) notation relative to Vienna-PeeDee Belemnite limestone (V-PDB) for *δ*^13^C and atmospheric nitrogen (AIR) for *δ*^15^N. Replicate measurements of internal laboratory standards (USGS-61 and USGS-63) in every batch (*n* = 20) indicated precision < 0.10 ‰ and < 0.15 ‰ for *δ*^13^C and *δ*^15^N values, respectively. Subsections with C: N ratio below 2.90 or above 3.99 were discarded [51], and C: N ratio was 2.91–3.99 in *G. fabricii* and 2.90–3.96 in *T. sagittatus* in the subsections used. The analyses were carried out at the Littoral Environnement et Sociétés, La Rochelle University (La Rochelle, France). Reused SIA data from Ref [54]. are based upon the analyses carried out at the Marine and Environmental Science Centre, University of Coimbra (Coimbra, Portugal). The analytical performance of these two laboratories has recently been compared with no differences found in *δ*^13^C and *δ*^15^N values [68].

### Data analyses

#### Fixation- and chitin-induced corrections

Neither ethanol nor formalin fixation significantly affect *δ*^13^C or *δ*^15^N signatures of chitinous cephalopod beaks [69], thus, no corrections were performed due to fixation. Values of *δ*^15^N in cephalopod beaks, in contrast to *δ*^13^C values, are on average 4.8‰ lower than values in muscle tissue [69–72]. Therefore, we added 4.8‰ to raw beak *δ*^15^N values when estimating TP as proposed by e.g. Refs [28, 54, 57, 72, 73].

#### Trophic position and correction for the suess effect

‘Trophic position’ (TP) term was used instead of ‘trophic level’, as more appropriate for natural food webs where predators prey on many prey species with mixed diet [74]. The standard equation was used to estimate TP from *δ*^15^N values [75]. ‘Classical’ trophic enrichment factor (TEF) *δ*^15^N = 3.4 ‰[76] was used for *T. sagittatus* in the North Atlantic, and ‘Arctic’ TEF *δ*^15^N = 3.8 ‰[77] was used for *G. fabricii* in the Arctic. Baseline *δ*^15^N values of phytoplankton as TP = 1.0 from the locations and years from each data point in the euphotic zone average (0–130 m) on the model grid (1.8°x3.6° horizontal resolution), which were obtained from the Model of Ocean Biogeochemistry and Isotopes (MOBI) [26, 27, 64, 65] (see detailed model description below), and are available online (see Data availability). Trophic positions for reused *δ*^15^N data from Ref [54]. were recalculated here in the similar way to ensure comparability. Correction of *δ*^13^C values for the Suess effect were performed by adding the correction values to raw values following Refs [68, 78]. Correction values were obtained from the locations and years from each data point in the euphotic zone average (0–130 m) on the model grid (1.8°x3.6° horizontal resolution), which were obtained from MOBI [26, 27, 64, 65] (see detailed model description below) and are available online (see Data availability). The isotope-enabled global ocean biogeochemical model simulations in MOBI were forced with transient hindcast scenarios of observed increasing atmospheric CO_2_ and decreasing atmospheric *δ*^13^CO_2_ (see model description below) [26, 27, 64, 65]. The *δ*^13^C data from Ref [54]. were reanalyzed here using a similar correction method to ensure comparability.

#### Specialization index

A specialization index (*s*) was used to estimate the degree of individual specialization [79, 80] based on the variation within and among individuals of the same species from a given time series [54, 81]. The specialization index (*s*) was defined as:

$$s = {\text{ }} \frac{ \Delta {\delta ^{13}}\text{C}{\text{ }}\left( {\text{or}{\text{ }}\Delta {\text{ TP}}} \right){\text{ }}\text{along\,the\,crest\,of\,individual}{\text{ }}}{ {\text{ }} \left( \begin{aligned}&\Delta {\delta ^{13}}\text{C}{\text{ }}\left( {\text{or}{\text{ }}\Delta {\text{ }}\text{TP}} \right){\text{ }}\text{among\,studied}\\&{\text{ }}\text{individuals\,from\,this\,time\,series}{\text{ }} \\&+ {\text{ }} \Delta {\delta ^{13}}\text{C}{\text{ }}\left( {\text{or}{\text{ }}\Delta {\text{ }}\text{TP}} \right){\text{ }}\\&\text{along\,the\,crest\,of\,individual} \end{aligned}\right)},$$

where Δ = variance [54, 81]. Accordingly, *s* values range from 0 to 1, with 1 representing a complete overlap between the individual and the population (= extreme generalist individual) and lower *s* values representing higher degrees of specialization [54, 81]. The specialization index (*s*) was calculated separately for each individual using corrected *δ*^13^C values and TP.

#### Correlations, statistical tests, generalized additive and mixed effect models and niche analyses

Correlations between raw *δ*^13^C and *δ*^15^N values in each individual were assessed using a Spearman’s rank correlation [82]. However, for all other analyses, corrected *δ*^13^C values and TP were used. A Mann–Whitney *U* test [82] was used to compare between two groups, e.g., recalculating from Ref [22]. (Table S2) or comparing corrected *δ*^13^C values from 1900s to 2010s within the Nordic Seas. A Kruskal–Wallis *H* test with a post-hoc Dunn’s *Z* test [82] was used to compare among more than two groups, e.g., corrected *δ*^13^C values, TP and *s* among the time series. All the tests mentioned above were two-tailed [82]. Differences in corrected *δ*^13^C values and TP among beak subsections were tested using a Skillings–Mack test proceeded by the Nemenyi post hoc test [83]: only the subsections common to all the beaks within a given time series were tested. The packages PMCMR 4.4 [84] and Skillings.Mack 1.10 [85] in R 4.1.3 [86] were used for these analyses. Effect size (Cohen’s *d*) was calculated by standard procedures where applicable [87].

Generalized additive models (GAMs) [88] were used to assess the temporal trends of corrected *δ*^13^C values, TP and *s*, using data from a particular subsection through time (= 1st and 8th). Each variable was assessed independently against year as a smoother. To assess the ontogenetic trends of corrected *δ*^13^C values and TP in the first eight subsections within every time series, generalized additive mixed effect models (GAMMs) were used to account for a repetitive sampling from the same individual. Each variable was assessed independently against subsection as a smoother with individual # as a factor. The smoothed term (*k*) to avoid overfitting and oversimplification was adapted for each GAM and GAMM based on the lowest Akaike’s Information Criterion adjusted for small sample sizes [88] (Table S3). The package mgcv 1.8–42 [89] in R 4.1.3 [86] was used for these analyses, and also to check the GAM and GAMM assumptions by the residual analyses (Table S4).

Sequential subsections of the same beak do not comply with the assumption of sample independence. Due to that, the package nicheROVER 1.1.0 [90] in R 4.1.3 [86] was chosen over the commonly used package SIBER [91] for isotopic niche analyses. Moreover, SIBER is known to be biased by low sample size [92], while we have no more than 13 beaks per time series (Table 1). Default Markov chain Monte Carlo settings from nicheROVER 1.1.0 [90] were employed (1,000 iterations and *α* = 0.95). The overlap interpretation among the isotopic niches followed Ref [93]. where overlap ranging from 0 to 0.29 indicated no overlap, from 0.30 to 0.60 indicated medium overlap, and from 0.61 to 1 indicated large overlap, with only the large overlap considered significant [54, 73]. Trophic positions were used instead of *δ*^15^N values (y axis) in niche estimations. This approach improves the ecological meaning of isotopic data when comparing across different years and areas, as it is a mean to counter high *δ*^15^N baseline variation [26, 27]. This approach has been repeatedly applied to cephalopods [28, 54, 73].

Statistical analyses and calculations were performed in R 4.1.3 [86] and PAST 4.12b [94]. The sample size is given wherever statistical results are reported, preferably as *n* of individuals and subsections. The value of *α* = 0.05 was considered significant in this study.

#### Description of model of ocean biogeochemistry and isotopes (MOBI)

The Model of Ocean Biogeochemistry and Isotopes (MOBI) is coupled within the global three dimensional ocean circulation component of the UVic Earth System Climate Model, version 2.9 [95]. MOBI predicts *δ*^13^C and *δ*^15^N isotope concentrations in all respective model tracers including baseline dissolved inorganic nutrients, phytoplankton and zooplankton trophic levels [26, 27, 65]. The model is comprehensively described in Refs [26, 27, 64, 65] and here we provide a brief description.

The two stable carbon isotopes, *δ*^12^C and *δ*^13^C, are included for dissolved inorganic carbon and organic carbon including phytoplankton, zooplankton, sinking particulate organic matter, and dissolved organic carbon. The most relevant processes determining the *δ*^13^C distribution in the model include air-sea gas exchange, physical ocean transport, biological uptake and remineralization of organic carbon [64]. To account for the Suess effect, a hindcast simulation with increasing atmospheric CO_2_ and decreasing *δ*^13^CO_2_ from atmospheric observations were applied to the model forcing, which results in a spatially varying Suess effect depending on the local ocean dynamics and biogeochemistry. The baseline phytoplankton *δ*^13^C decline averaged over our study region (35°N–75°N, 75°W–0°) from year 1850 to 2023 is 3.2 ‰, of which 0.78 ‰ is due to the Suess effect and 2.3 ‰ is due to increased phytoplankton fractionation under higher atmospheric CO_2_ concentrations in the model.

The two stable nitrogen isotopes, *δ*^14^N and *δ*^15^N, are included in nitrate and organic nitrogen including phytoplankton, zooplankton, sinking particulate organic matter, and dissolved organic nitrogen. The processes in the model that fractionate the nitrogen isotopes (i.e., preferentially incorporate *δ*^14^N into the product) are phytoplankton NO_3_ assimilation (6 ‰), zooplankton excretion (4 ‰), N_2_ fixation (-1 ‰), water column denitrification (22 ‰) and benthic denitrification (6 ‰), in which the respective fractionation factor yields the *δ*^15^N difference between substrate and product [65]. In the high latitude North Atlantic, water column denitrification and N_2_ fixation occur at insufficient rates to significantly affect the *δ*^15^N distribution. Therefore, the nitrate utilization by phytoplankton drives the major meridional gradient of decreasing *δ*^15^N values by 2.2 ‰ from 35°N to the nitrate maximum at 60°N, with this trend reversing towards even higher latitudes due to lower nitrate availability there. The transient change from 1850 to 2023 averaged over the region is relatively small (0.05 ‰).

## Results

### Temporal trends

#### *Gonatus fabricii*

**Fig. 2:**
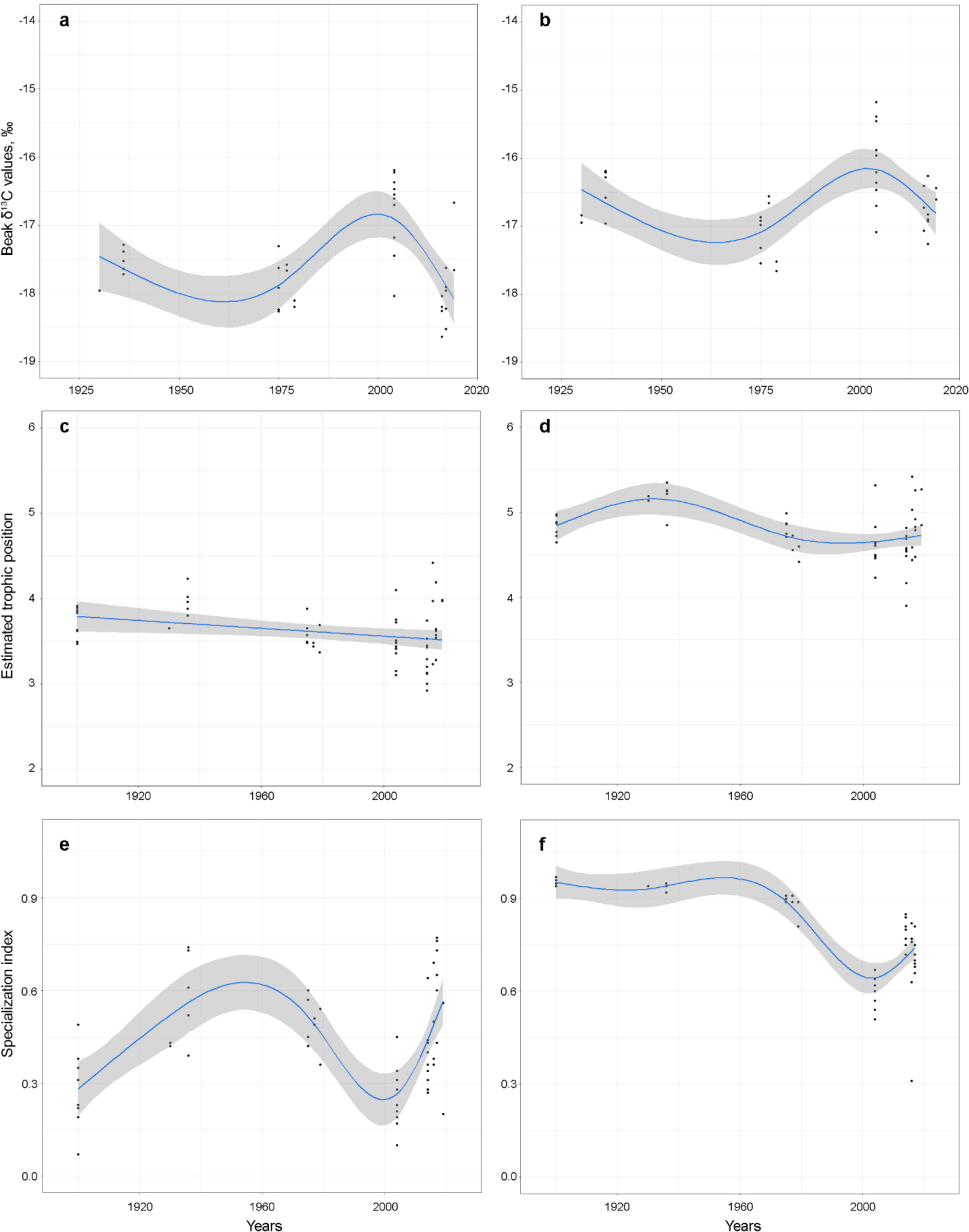
Long-term trends in *δ*^13^C values (**a**, **b**) between 1930 and 2019, and estimated trophic position (**c**, **d**) and specialization index (**e**, **f**) of *Gonatus fabricii* between 1900 and 2019. **a**, **c**. Squid with mantle length (ML) 6.6–7.5 mm (= beak subsection 1). **b**, **d**. Squid ML 54.9–64.5 mm (= beak subsection 8). **e**. Specialization index of *δ*^13^C, entire beaks. **f**. Specialization index of trophic position, entire beaks. Blue line represents generalized additive model and grey area represents confidence intervals. All individual data points are shown

**Fig. 3:**
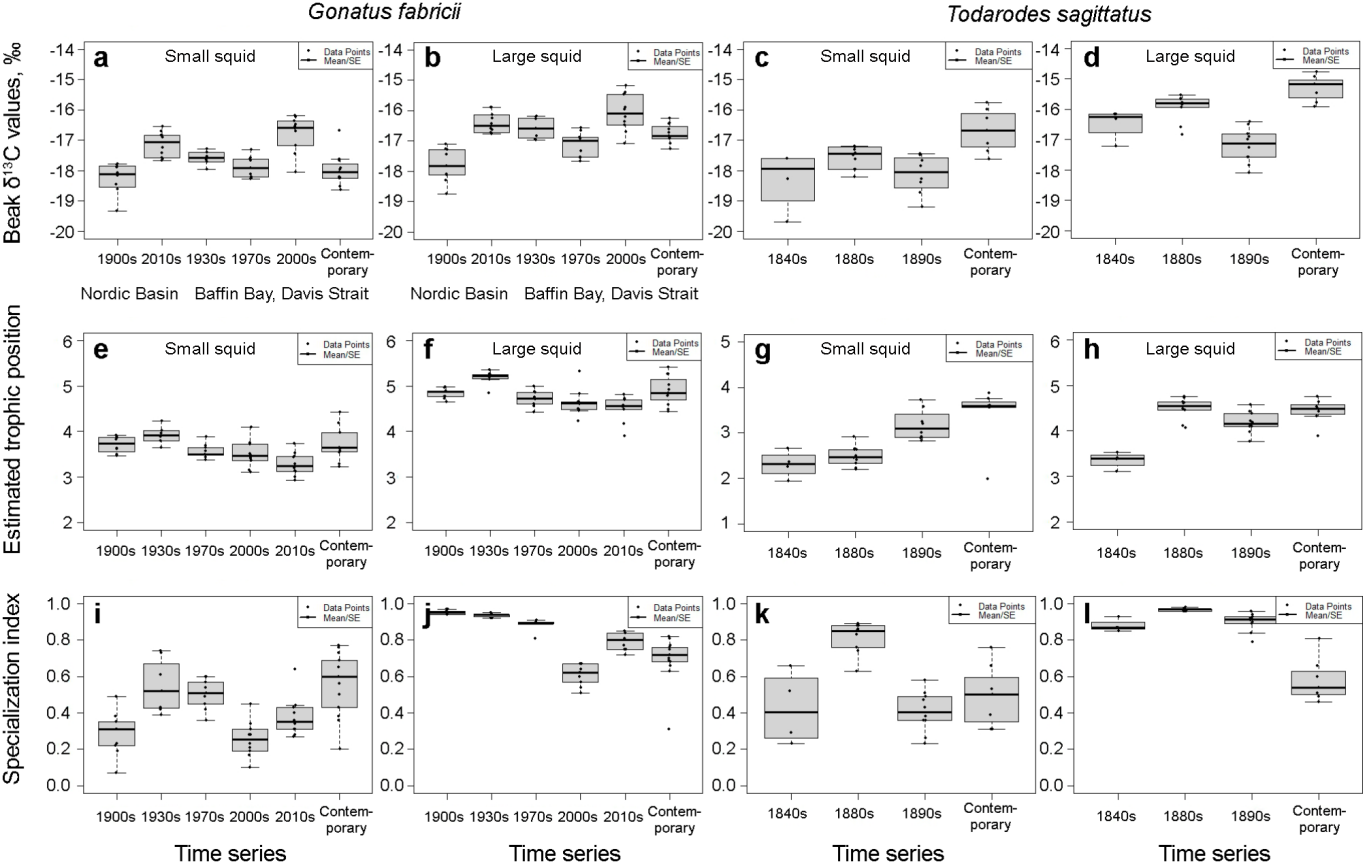
Differences in *δ*^13^C values (**a**–**d**), estimated trophic position (**e**–**h**) and specialization index of *δ*^13^C (**i**, **k**) and trophic position (**j**, **l**) among different time series of *Gonatus fabricii* and *Todarodes sagittatus* between 1844 and 2023. **a**, **e**. *Gonatus fabricii* with mantle length (ML) 6.6–7.5 mm (= beak subsection 1). **b**, **f**. *Gonatus fabricii* ML 54.9–64.5 mm (= beak subsection 8). **c**, **g**. *Todarodes sagittatus* ML 9.1–10.0 mm (= beak subsection 1). **d**, **h**. *Todarodes sagittatus* ML 72.7–80.4 mm (= beak subsection 8). **i**. Specialization index of *δ*^13^C, entire beaks of *G. fabricii*. **j**. Specialization index of trophic position, entire beaks of *G. fabricii*. **k**. Specialization index of *δ*^13^C, entire beaks of *T. sagittatus*. **l**. Specialization index of trophic position, entire beaks of *T. sagittatus*. Boxplots indicate mean values (midlines), upper and lower quartiles (box limits) and standard errors (whiskers). All individual data points are shown. Figures from subsection 1 are labelled as small squid, and figures from subsection 8 are labelled as large squid

Only subsections 1^st^ and 8^th^ (= the last common to all beaks) were used to study temporal trends, which represented squid with mantle length (ML) 6.6–7.5 mm (small) and 54.9–64.5 mm (large), respectively. Significant differences were found for *δ*^13^C values among the time series in both small and large squid, and in both Baffin Bay and Nordic Seas (Figs. 2 & 3; Tables 2 & S5). Different *δ*^13^C values in *G. fabricii* from these areas [57] precluded the comparison between them in temporal analyses of *δ*^13^C values. Generalized additive models (GAMs) suggested more substantial differences among the time series earlier in ontogeny (Fig. 2; Table 2). Moreover, GAM for small squid explained more variation (Table 2). Overall, the significant temporal trend from the available time series highlighted by both GAMs and Kruskal–Wallis *H* test was: (a) small decrease from 1930s to 1960s; (b) increase from 1960s to 1990s/2000s; and (c) ongoing decrease since 1990s/2000s (Fig. 2; Tables 2 & S5).

Significant differences in TP among the time series were found in both small and large squid (Figs. 2 & 3; Tables 2 & S5). Differences were more substantial in large squid (Fig. 2; Table 2). GAM for large squid explained much more variation (Table 2). The significant temporal trend in large squid from the available time series, again highlighted by both GAMs and Kruskal–Wallis *H* test, was: (a) small increase from 1900s to 1930s; (b) decrease from 1930s to 1990s; and (c) ongoing small increase since 1990s (Fig. 2; Tables 2 & S5).

**Table 2:** Outputs of Generalized Additive Models (GAMs) of long-term trends in *δ*^13^C values, Trophic Position (TP) and Specialization index (*s*) of *Gonatus fabricii*. In GAMs, *k* indicates the number of knots and *s* represents the smoother (year), where values are effective degrees of freedom. *n* – sample size. Significant *p*-values are in **bold**

δ^13^C							TP						
Subsection 1							Subsection 1						
Time	*n*	Intercept (mean ± SE)	*k*	*s*(Year)	*p*	Deviance explained, %	Time	*n*	Intercept (mean ± SE)	*k*	*s*(Year)	*p*	Deviance explained, %
1930–2019^1^	36	-17.56 ± 0.09	4	2.96	**0.0003**	45.3	1900–2019	54	3.60 ± 0.04	3	1.00	**0.0255**	9.2
Subsection 8							Subsection 8						
1930–2019^1^	37	-16.62 ± 0.08	4	2.94	**0.0009**	40.0	1900–2019	56	4.78 ± 0.04	4	2.91	**0.0004**	30.2
*s*							*s*						
1900–2019	58	0.42 ± 0.02	5	3.93	**< 0.0001**	46.2	1900–2019	58	0.80 ± 0.01	5	3.94	**< 0.0001**	70.1

^1^Baffin Bay data only, as *δ*^13^C values in *G. fabricii* from the Baffin Bay and Nordic Seas are proven to be significantly different [57]

Both *δ*^13^C values’ and TP’s specialization indices (*s*) showed a significant temporal change (Figs. 2 & 3; Tables 2 & S5). For *s* of *δ*^13^C values the significant temporal trend from the available time series was: (a) increase from 1900s to 1950s; (b) decrease from 1950s to 1990s; and (c) ongoing increase since 1990s (Fig. 2; Tables 2 & S5). For *s* of TP, the significant temporal trend from the available time series was: (a) no changes from 1900s to 1960s; (b) decrease from 1960s to 2000s; and (c) ongoing increase since 2000s (Fig. 2; Tables 3 & S5). GAM of TP’s *s* explained more variation, than that of *δ*^13^C values (Table 2). Overall, GAMs did not coincide in temporal patterns, except for the last time series: there was an increase of *δ*^13^C values’ *s*, TP’s *s* and TP and decrease of *δ*^13^C values starting from 1990s/2000s (Fig. 2).

**Table 3:** Ontogenetic changes in *δ*^13^C values and Trophic Position (TP) of *Gonatus fabricii*. *n* – sample size, GAMM – generalized additive mixed effect model, n/a – not applicable. Significant *p*-values are in **bold**

δ^13^C											
Time series	*n*		Main increase		After main increase		*s*, min–max (mean ± SE)	GAMM			
	individuals	subsections	rate	subsections	rate	subsections		Intercept (mean ± SE)	*k, s*(Subsection)^1^	*p*	Deviance explained, %
Late XIX^th^ century	2	32	0.41 ‰	1 to 4	-0.01 ‰	4 to 15	n/a	-16.67 ± 0.36	4, 2.99	**< 0.0001**	99.1
1900s	9	73	0.33 ‰	1 to 4	-0.13 ‰	4 to 8	0.07–0.49 (0.28 ± 0.04)	-17.63 ± 0.16	4, 2.91	**< 0.0001**	90.9
1930s	7	91	0.39 ‰	1 to 4	-0.06 ‰	4 to 12	0.39–0.74 (0.55 ± 0.06)	-16.73 ± 0.16	4, 2.93	**< 0.0001**	92.5
1970s	9	101	0.35 ‰	1 to 4	-0.02 ‰	4 to 12	0.36–0.60 (0.50 ± 0.03)	-17.14 ± 0.11	3, 2.00	**< 0.0001**	91.7
2000s	10	123	0.34 ‰	1 to 4	-0.06 ‰	4 to 11	0.10–0.45 (0.26 ± 0.03)	-16.06 ± 0.17	4, 2.85	**< 0.0001**	89.1
2010s	10	153	0.30 ‰	1 to 4	-0.04 ‰	4 to 11^2^	0.27–0.64 (0.38 ± 0.03)	-16.52 ± 0.13	3, 1.97	**< 0.0001**	78.7
Contemporary (2016, 2017, 2019)^3^	13	157	0.29 ‰	1 to 5	0.01 ‰	5 to 15	0.20–0.67 (0.56 ± 0.05)	-17.17 ± 0.12	3, 1.93	**< 0.0001**	80.7
TP											
Late XIX^th^ century	2	32	0.30 TP	1 to 5	0.02 TP	5 to 15	n/a	4.44 ± 0.56	4, 2.87	**< 0.0001**	98.3
1900s	9	73	0.24 TP	1 to 5	0.06 TP	5 to 8	0.94–0.97 (0.95 ± 0.01)	4.42 ± 0.04	4, 2.93	**< 0.0001**	94.7
1930s	7	91	0.23 TP	2 to 7	0.02 TP	7 to 12	0.92–0.95 (0.93 ± 0.01)	4.62 ± 0.05	4, 2.96	**< 0.0001**	95.6
1970s	9	101	0.28 TP	3 to 6	0.05 TP	6 to 12	0.81–0.91 (0.89 ± 0.01)	4.16 ± 0.06	4, 2.98	**< 0.0001**	96.1
2000s	10	123	0.31 TP	2 to 6	0.04 TP	6 to 12	0.51–0.67 (0.61 ± 0.02)	4.08 ± 0.09	4, 2.97	**< 0.0001**	96.3
2010s	10	153	0.21 TP	1 to 6	0.05 TP	6 to 15	0.72–0.85 (0.79 ± 0.01)	3.93 ± 0.09	3, 1.00	**< 0.0001**	89.6
Contemporary (2016, 2017, 2019)^3^	13	165	0.22 TP	2 to 7	0.04 TP	7 to 15	0.31–0.82 (0.69 ± 0.04)	4.38 ± 0.09	3, 1.00	**< 0.0001**	88.2

^1^in GAMMs, *k* indicates the number of knots and *s* represents the smoother (subsection), where values are effective degrees of freedom;

^2^there is a secondary increase of 0.20 ‰/subsection at subsections 11 to 15;

^3^published in Golikov et al. 2022 [54] and reused in this study

Niches after 2000s were wider than those of previous time series in both small and large squid (Fig. S2; Table S6). Niches from historical time series overlapped more with contemporary (= 2016, 2017 and 2019 in *G. fabricii*, see Methods) ones in both small and large squid, than vice versa (Fig. S2; Table S7).

Within-individual correlation between *δ*^13^C and *δ*^15^N values only presented in substantial proportion in 2010s and contemporary squid, being completely absent prior to 2000s (Table S8). The reasons for that are outlined in ontogenetic trends section, below.

#### *Todarodes sagittatus*

Similar to *G. fabricii*, only subsections 1^st^ (small; ML 9.1–10.0 mm) and 8^th^ (large; ML 72.7–80.4 mm) were used. Significant differences in both *δ*^13^C values and TP among the time series were found in both small and large squid with both GAMs and Kruskal–Wallis *H* test (Fig. 3 & S3; Tables S9 & S10). GAMs suggested more substantial differences for both *δ*^13^C values and TP among the time series later in ontogeny (Fig. S3; Table S10). GAMs for large squid explained more variation (Table S10). Specialization indices (*s*) of *δ*^13^C values and TP both showed significant differences among the time series (Figs. 3 & S3; Tables S9 & S10). GAM of TP’*s* explained more variation, than that of *δ*^13^C values (Table S10). We did not rely entirely on the temporal trends from GAMs (Fig. S3), because we only had samples from the XIX^th^ and XXI^st^ centuries (see Methods). Other methods of analyses, however, rendered similar results (Fig. 3; Table S9).

Niches from the XIX^th^ century were narrower than contemporary (= 2016–2018 and 2023 in *T. sagittatus*, see Methods) in small squid (Fig. S2; Table S6). In the large squid, niches from the contemporary and 1890s were wider than those from 1840s to 1880s (Fig. S2; Table S6). Niches from the XIX^th^ century overlapped more with contemporary ones in small squid (Fig. S2; Table S7). In large squid, this temporal overlap was similar (Fig. S2; Table S7).

Within-individual correlation between *δ*^13^C and *δ*^15^N values presented in substantial proportion in all the time series (Table S8).

### Ontogenetic trends

#### *Gonatus fabricii*

Significant ontogenetic increase of *δ*^13^C values and TP was found within all the time series (Figs. S4 & S5; Tables 3, S11 & S12). The main increase of *δ*^13^C values took place in squid from ML 6.6–7.5 mm to 23.4–27.5 mm in all but contemporary time series. In the latter, it took place from ML 7.0–7.4 mm to 30.9–35.2 mm (Fig. S4; Table 3). Slight decrease after the subsection 4 (ML 23.4–27.5 mm) was well-pronounced in all the time series prior to 2000s. The rate of change in *δ*^13^C values suggests it was only absent in the contemporary time series (Fig. S4; Table 3). The main ontogenetic increase of TP varied among time series, but always ended at ML 46.3–54.4 mm. Afterwards the TP increase continued at a 4–15 times slower rate throughout ontogeny (Fig. S5; Table 3). GAMs of ontogenetic changes in TP always explained more variation than in *δ*^13^C values (Table 3). GAMs of ontogenetic changes in *δ*^13^C values and TP suggested smoother changes after 2000s than in the previous time series (Figs. S4 & S5; Table 3).

No pattern of ontogenetic changes in niche width was found (Table S6). The narrowest niches were frequently found in beak subsections 2 and 10 (= ML 11.5–12.8 and 76.0–109.3 mm), and the widest in subsections 4, 3 and 9 (= ML 23.4–27.5, 17.1–19.7 and 65.0–85.6 mm). Finally, the medium-sized niches were most frequently in beak subsections 5, 2 and 14 (= ML 30.5–35.9, 11.5–12.8 and 133.0–190.3 mm) (Table S6). Thus, ontogenetic patterns of niche width are not further discussed in this study.

Niche overlap pattern was used to classify the history of niche changes of the species to four consecutive periods (Figs. S6 & S7, Tables S13 & S14). The initial period had a limited overlap with other beak subsections (Fig. S7). The 1st stable period followed with an increased overlap between the subsections (Fig. S7). The change period followed where the main shifts of overlap occurred (Fig. S7). Finally, the main stable period was the last one, and it exhibited large overlap among all beak subsections (Fig. S7). A decrease in overlap was observed in every time series prior to 2000s during the change period (Figs. S6 & S7, Tables S13 & S14). The onset of the main stable period after ML 38.1–64.5 mm means squid have bypassed the main shifts in its diet and habitat. However, both *δ*^13^C values and TP continue to change throughout ontogeny, albeit at smaller rates (Fig. S6, Tables 3, S11, S12, S13 & S14). As a result, occasional secondary shifts occurred during the main stable period, as evident in 1930s and 2010s (Tables S13 & S14). Prolonged initial periods and shortened change periods occasionally occurred in the time series after 2000s (Tables S13 & S14).

#### *Todarodes sagittatus*

Significant ontogenetic increase of *δ*^13^C values and TP was present within all the time series (Fig. S8; Tables S15, S16 & S17). The main ontogenetic increase of *δ*^13^C values took place in squid from ML 9.1–10.0 mm to 45.6–50.2 mm in all but 1890s time series. In 1890s, it took place from ML 9.1–10.0 mm to 27.9–30.0 mm (Fig. S8; Table S15). The main ontogenetic increase of TP varied among time series, but always over at ML 63.9–70.3 mm (= beak subsection 7). Afterwards, the TP increase continued with a slower rate throughout ontogeny (Fig. S8; Table S15). GAMs of ontogenetic changes in TP always explained more variation than in *δ*^13^C values, except for the contemporary time series (Table S15).

Niches from small squid (ML 9.1–40.1 mm; = beak subsections 1–4) were most frequently the widest (Fig. S9; Table S6). The narrowest and most frequently medium-sized niches strongly varied among the time series (Table S6). Squid with ML larger than 46.6 mm had their niches completely overlapped in the contemporary time series (Fig. S9; Table S18). All of the XIX^th^ century squid showed overlap shrinkage in mid- to late ontogeny (Fig. S9; Table S18).

## Discussion

Increased environmental effects of climate change in the Arctic are especially marked since the late 1990s/early 2000s, following the continuous increase in mean annual temperatures [2, 17]. In turn, temperature increase causes sea-ice melting, reduction in snow cover, and changes in physical ocean dynamics [3, 4]. These environmental effects lead to changes in the ecosystems, which mainly include increased generalization of the food webs, higher primary production, and inflow of boreal species into the Arctic marine ecosystems [3–5, 7–11, 16, 18, 19]. The retrospective SIA studies from the Arctic and North Atlantic to date use basal (= filter feeders) and top (= marine mammal top predators) food web components [21, 22, 31, 32, 34–41, 43, 44]. Shifts in their trophic ecology, which would coincide in time with the mentioned increased environmental impact of climate change since the late 1990s/early 2000s, were only evident in narwhals and ringed seals from East and West Greenland [21, 22, 31, 32, 34–41, 43, 44]. Ontogenetic changes and different timing of temporal changes in trophic ecology, however, were often found [21, 22, 31, 32, 34–41, 43, 44]. Our study showed that in an abundant short-living invertebrate mesopredator, the Arctic squid *G. fabricii*, the changes in trophic ecology were observed since 1990s/2000s. Specifically, we observed an increase of trophic position (TP), specialization indices (*s*) in TP and *δ*^13^C values, niche width, and a decrease of *δ*^13^C values. These trends were generally more evident in large squid (see below for reasons).

The main climate-driven shifts in the Arctic marine ecosystems are increased generalization of the food webs, higher primary production, and inflow of boreal species [3–5, 7–11, 16, 18, 19]. These shifts may explain temporal trends of TP, *s* of TP and niche width, which on their turn reflect changes in diet of *G. fabricii* over time. Trophic position and niche width of herbivorous fishes and piscivorous seabirds decrease with increasing primary production (= food availability), as they specialize on particular targeted prey sources when they become abundant [96, 97]. Top predators can either decrease or increase TP with increasing primary production, either specializing on a particular food source or broadening their food spectra [21]. Opportunistic invertebrates, such as copepods and Ommastrephidae squids, have only demonstrated increase of TP and niche size with increasing primary production (i.e., they broaden their food spectra with increasing food availability [98–100]). Increasing TP in contemporary *G. fabricii* suggests that they hunt larger fishes or cephalopods. Capture of large prey has been documented by in situ observations and stomach contents analyses in other gonatid squids [101, 102]. As a result of Atlantification there is a migration of large, boreal, piscivorous fishes with a generalist diet into the Arctic ecosystems [10, 11, 18]. *Gonatus fabricii* change their crustacean diet to piscivorous and cannibalistic as they grow [54, 57, 58]. Therefore, large piscivorous fishes arriving from the boreal Atlantic and becoming abundant in the Arctic [10, 11, 18] may now be the dominant prey for large *G. fabricii*, which can explain the more pronounced temporal change in TP in large *G. fabricii*, than in small *G. fabricii*.

Increased niche width in *G. fabricii* after 2000s denotes a more generalist diet, as observed in copepods, other squids, fishes and seabirds in tropical and temperate areas [96–100]. This supports increased generalism of *G. fabricii* diet shown by specialization index (*s*). Temporal trends of *s* (both TP and *δ*^13^C values) explain more variation than TP and *δ*^13^C values. Both *δ*^13^C values and *s* of *δ*^13^C are more temporally variable, than their TP counterparts. Before 1960s, in times of fewer temperature anomalies [2] and lower fisheries pressure [103, 104] in the Arctic, *s* of TP in *G. fabricii* was the highest over the study period. It indicates very broad generalist diet in less disturbed ecosystems, in line with dietary opportunistic generalism known for squids [9, 57, 60, 61, 99–101]. Decrease in diet generalism from 1960s, in this case, suggests increase in ecosystem disturbance by more frequent temperature anomalies [2] and increasing fisheries pressure [103, 104]. Cephalopods are known to adapt well and eventually can adapt to short-term climate change impact [9, 28, 47, 48, 73]. Increase of TP and diet generalism (shown by both niche width and *s*) after 1990s/2000s suggests that *G. fabricii* in the low latitude Arctic is well adapted to cope with the ecosystems’ Atlantification. The degree of generalism in *G. fabricii*, however, still did not reach the values associated with the relatively less disturbed ecosystems of before the 1960s. Contemporary *T. sagittatus* in the high latitude North Atlantic also demonstrate increase niche width of contemporary vs. the XIX^th^ century squid, which is the same indirect evidence of a more generalist diet, as in *G. fabricii*. However, (a) *T. sagittatus* is under fisheries’ pressure [60, 61]; (b) climate change impact in the area is not as strong as in the Arctic [1, 2]; and (c) we only have samples of this species from the XIX^th^ and XXI^st^ centuries. As such, a link to climate change is not evident in *T. sagittatus*, unlike in *G. fabricii* from the low latitude Arctic.

Ontogenetic changes in *δ*^13^C values and TP in *G. fabricii* are less pronounced in 2000s, 2010s and contemporary time series, than in all of the earlier time series. This pattern matches with increased climate change effects in the Arctic [2, 17]. The consecutive ontogenetic isotopic niches in this species became more similar over time. These observations indicate less abrupt changes in diet and habitat throughout the species’ ontogeny in contemporary individuals compared to historical individuals. The reason for this difference may be that during the climate-driven alteration of the marine ecosystems, biodiversity, primary production and food web generalism also increase [3–5, 7–11, 16, 18, 19]. As such, the spectra of available prey for squid can increase in both diversity and abundance.

Previous SIA studies in contemporary *G. fabricii* support our results on ontogenetic changes in *δ*^13^C values and TP [54, 57, 105]. In *T. sagittatus*, ontogenetic increase of *δ*^13^C values and TP is reported for the first time. Small squids (ML 6.6–7.5 mm in *G. fabricii* and ML 9.1–10.0 mm in *T. sagittatus*) feed largely on copepods [54, 58, 60–62]. This may explain why almost all trophic ecology characters we assessed through SIA in *G. fabricii*, and all such characters in *T. sagittatus* were better expressed in large squids. Early paralarvae of *T. sagittatus* are detritivorous [63], which is reflected in lowered TP of small *T. sagittatus* in comparison to small *G. fabricii*. Large squids have almost similar TPs. The early paralarval stage, however, does not constitute more than 10% of the subsection 1 of the beak in any of the species, judging by size of early paralarvae beaks in other Ommastrephidae [106]. It means small *T. sagittatus* feed on lower TP prey, than small *G. fabricii*.

One of the common limitations of retrospective ecology studies is sample availability (e.g., Refs [35, 38, 68, 78]). This leads to non-selective use of all available material, and consecutive comparisons of individuals of different sizes among different periods. While we had no control over the availability of the historical samples, we addressed the latter issue by using large beaks, which were cut into subsections. The common subsections among all studied beaks were used for ontogenetic GAMMs, and similar subsections (1st and 8th) were used for temporal trend GAMs among the time series. This allowed us to compare squid of similar sizes through time. Moreover, it allows the more effective application of techniques that are not sensitive to sample size [52, 54] (see Methods), and allows to test for specialization [54, 81]. The specialization index (*s*) shows the degree of generalism/specialism based on within- and among-individual variation [54, 81]. Analyzing beaks by subsections also allowed us to find ontogenetic trends in *δ*^13^C values and TP. It would be impossible to achieve these comparisons if using whole beaks due to sample variations and different beak sizes. Furthermore, the mixing of different SI signatures along ontogeny would result in averaged values, potentially masking any discernible trend. Another common caveat of retrospective isotopic ecology studies is related to high baseline variation of *δ*^13^C and *δ*^15^N values in space and time [25–27], and the Suess effect [29]. To overcome this issue, we used baseline and correction values the Model of Ocean Biogeochemistry and Isotopes (MOBI) [26, 27, 64, 65]; these values together with raw data are available online (see Data availability). The MOBI is a global model capable of hindcast simulations from the preindustrial era [26, 27, 64, 65], and a data-constrained isotope model [26, 27, 64, 65], that has been successfully applied to large-scale stable isotope ecological studies (e.g., Refs [107–109]).

## Conclusion

Our study highlights a shift in the trophic ecology of an Arctic squid species in the late 1990s/early 2000s over the low latitude Arctic areas. Specifically, *G. fabricii* demonstrates an increase in diet and habitat use generalism (= opportunistic choice rather than specialization), TP and niche width. These temporal changes coincide with the increase of environmental impact of climate change in the Arctic (warming temperatures, sea-ice melting, reduction in snow cover, and changes in physical ocean dynamics) [2, 17]. The climate-driven shifts in the Arctic ecosystems (i.e., generalization and Atlantification of food webs, increased presence of boreal species new to the area and increased primary production [3–5, 7–11, 16, 18, 19]) potentially explain the observed shifts in the trophic ecology of the Arctic squid species. Specifically, increased generalization of food webs coincides with increased diet generalism and niche width of this squid, while increased abundance of boreal piscivorous fishes may be responsible for the squid’s increased TP. We suggest that abundant and ecologically important opportunistic mesopredatory invertebrates are good model organisms to study marine ecosystem changes via retrospective analysis. Moreover, the high degree of opportunism and adaptability of squids enables them to adjust their trophic ecology, and as such makes them potential winners of short-term shifts in Arctic ecosystems.

### Electronic supplementary material

Below is the link to the electronic supplementary material.

Supplementary Material 1

## Data Availability

All relevant data are included in the paper and/or in the supplementary materials; raw data and primary analysis results, δ^13^C correction values for the Suess effect and δ^15^N baseline values used to estimate TP are available from Zenodo (https://zenodo.org/record/11108528 and https://zenodo.org/record/8256576), and PANGAEA (https://doi.pangaea.de/10.1594/PANGAEA.968467); reused data from Golikov et al. (2022) [54] are available in the respective paper and its supplementary materials, and from Zenodo repository (https://zenodo.org/record/7113726); MOBI-UVic model code is publicly available on GitHub (https://github.com/chrissomes/UVic2.9).
