# Supplementary material for: Insights on long-term ecosystem changes from stable isotopes in historical squid beaks
Source: BMC Ecol Evol. 2024 Jul 3;24:90. doi: 10.1186/s12862-024-02274-7 (PMC11221165; doi:10.1186/s12862-024-02274-7)
Supplement: Supplementary file 1 — Supplementary Material 1 [file 12862_2024_2274_MOESM1_ESM.docx]

**Supplementary materials for:**

**Insights on long-term ecosystem changes from stable isotopes in historical squid samples**

Alexey V. Golikov^1*^, José C. Xavier^2,3^, Filipe R. Ceia^2^, José P. Queirós^2,3^, Paco Bustamante^4^, Bram Couperus^5^, Gaël Guillou^4^, Anna M. Larionova^6^, Rushan M. Sabirov^6^, Christopher J. Somes^1^ & Henk-Jan Hoving^1^

^1^GEOMAR Helmholtz Centre for Ocean Research Kiel, Kiel, Germany

^2^University of Coimbra, MARE—Marine and Environmental Sciences Centre/ARNET—Aquatic Research Network, Department of Life Sciences, Coimbra, Portugal

^3^British Antarctic Survey, Natural Environment Research Council, Cambridge, UK

^4^Littoral Environnement et Sociétés (LIENSs), UMR 7266, CNRS-La Rochelle Université, La Rochelle, France

^5^Wageningen Marine Research, Wageningen University and Research, IJmuiden, The Netherlands

^6^Department of Zoology, Kazan Federal University, Kazan, Russia

^*^Correspondence should be addressed to A.V.G. (email: golikov.ksu@gmail.com)

**This file contains:**

Tables S1 to S18;

Figures S1 to S9;

References (all also cited in the main text).

Table S1. Summary of the retrospective bulk stable isotope studies on marine mammals from the Arctic and adjacent areas of the North Atlantic

| Area | Species | Tissue | Timeline | *δ*^13^C temporal pattern | *δ*^15^N temporal pattern | Reference |
| --- | --- | --- | --- | --- | --- | --- |
| Canadian Eastern  Arctic, Labrador | Beluga whale  (*Delphinapterus leucas*) | Teeth, by  growth layers | 1963–2008 | Decreasing | Not used in  this study | Matthews,  Ferguson 2014 |
|  | Killer whale  (*Orcinus orca*) |  | 1944–1999 |  |  |  |
| Canadian Eastern  Arctic | Beluga whale  (*Delphinapterus leucas*) | Muscle | 1990–2002  compared with  2005–2012 | Older values  significantly higher  than newer values^1^ | Older values significantly higher  than newer values^1^ | Yurkowski  *et al.* 2018 |
|  | Ringed seal  (*Pusa hispida*) |  |  |  | No differences between  older and newer values^1^ |  |
| Canadian Eastern  Arctic | Narwhal (*Monodon*  *Monoceros*) | Tusks, by  growth layers | 1982 and 1983  compared with  2015 and 2017 | Older values  significantly higher  than newer values | Older values significantly higher  than newer values | Zhao  *et al.* 2022 |
| Baffin Bay | Narwhal (*Monodon*  *Monoceros*) |  | 1961–2010 | Non-linear decreasing | Non-linear; increasing from late 1960s  to late 1990s, decreasing from late  1990s to early 2010s | Dietz  *et al.* 2021 |
| Baffin Bay | Polar bear  (*Ursus martimus*) | Hairs | 1987–2008 | No trend | No trend | Dietz  *et al.* 2011 |
| Baffin Bay | Ringed seal  (*Pusa hispida*) | Teeth,  entire | 1982–2006 | Non-linear; decreasing from early 1980s  to late 1990s, increasing from late  1990s to mid-2000s | Non-linear; no changes from early 1980s  to mid-1990s, decreasing from mid-1990s  to late 1990s, increasing in early 2000s,  decreasing in mid-2000s | Aubail  *et al.* 2010 |
| East Greenland | Ringed seal  (*Pusa hispida*) |  | 1986–2006 | Non-linear; decreasing from mid-  1980s to mid-2000s | Non-linear; increasing from early 1980s  to mid-1990s, decreasing from mid-1990s  to late 1990s, increasing in early 2000s,  decreasing in mid-2000s |  |
| Svalbard | Polar bear  (*Ursus martimus*) |  | 1964–2003 | Non-linear; increasing in mid-1960s,  decreasing in late 1960s, increasing from  late 1960s to early 1980s, decreasing  from early 1980s to mid-2000s | Non-linear; increasing from mid-1960s to late  1970s, decreasing from late 1970s to mid-2000s | Aubail  *et al.* 2012 |

^1^differences were assessed by A.V.G. based on raw data from Yurkowski *et al.* 2018, see below (Table S2).

Table S2. Differences in *δ*^13^C and *δ*^15^N values in beluga whale (*Delphinapterus leucas*) and ringed seal (*Pusa hispida*) from 1990–2002 and 2005–2012 in the Canadian Eastern Arctic, using raw data from Yurkowski *et al.* 2018. Mann–Whitney *U* test are provided in the table. *n* – sample size. Significant *p*-values are in **bold**

| Species | *δ*^13^C values | | | *δ*^15^N values | | |
| --- | --- | --- | --- | --- | --- | --- |
|  | 1990–2002:  *n*, mean ± SD | 2005–2012:  *n*, mean ± SD | Differences | 1990–2002:  *n*, mean ± SD | 2005–2012:  *n*, mean ± SD | Differences |
| Beluga whale  (*Delphinapterus*  *leucas*) | 63,  -18.1 ± 0.04 ‰ | 25,  -18.3 ± 0.08 ‰ | *d* = 0.45,  *U* = 567.5,  *p* = **0.0422** | 175,  -18.7 ± 0.04 ‰ | 53,  -19.1 ± 0.04 ‰ | *d* = 1.99,  *U* = 154.0,  *p* < **0.0001** |
| Ringed seal  (*Pusa hispida*) | 63,  17.6 ± 0.13 ‰ | 25,  15.9 ± 0.16 ‰ | *d* = 73.16,  *U* = 2950.0,  *p* < **0.0001** | 175,  15.2 ± 0.07 ‰ | 53,  15.0 ± 0.11 ‰ | *d* = 0.16,  *U* = 4124.0,  *p* = 0.22 |

Table S3. Aikaike’s Information Criterion (AIC) adjusted for small sample sizes used to choose right *k* (the number of knots) for Generalized Additive Models (GAMs) used for temporal trends and for Generalized Additive Mixed Effect Models (GAMMs) used for ontogenetic trends. AIC_c_ – Aikaike’s Information Criterion adjusted for small sample sizes, TP – trophic position, *s* – specialization index, *n* – sample size. GAMMs marked with an asterisk (*). Chosen *k* values (= the lowest AIC_c_) are in **bold**

| Model (GAM or  GAMM) | *Gonatus fabricii* | | | | | | Model (GAM or  GAMM) | *Todarodes sagittatus* | | | | | |
| --- | --- | --- | --- | --- | --- | --- | --- | --- | --- | --- | --- | --- | --- |
|  | *δ*^13^C values | | | TP | | |  | *δ*^13^C values | | | TP | | |
|  | *n* | *k* | *AIC_c_* | *n* | *k* | *AIC_c_* |  | *n* | *k* | *AIC_c_* | *n* | *k* | *AIC_c_* |
| Late XIXth century* | 16 | 6 | 4.25 | 16 | 6 | 20.58 | 1840s* | 32 | 6 | 90.86 | 32 | 6 | 12.10 |
|  |  | 5 | -5.74 |  | 5 | 16.35 |  |  | 5 | 86.43 |  | 5 | 8.01 |
|  |  | **4** | **-12.74** |  | **4** | **8.06** |  |  | 4 | 82.38 |  | 4 | 5.91 |
|  |  | 3 | 22.37 |  | 3 | 10.40 |  |  | **3** | **78.67** |  | **3** | **1.45** |
| 1900s* | 66 | 6 | 23.76 | 66 | 6 | -43.12 | 1880s* | 80 | 6 | 17.02 | 80 | 6 | 34.89 |
|  |  | 5 | 19.98 |  | 5 | -46.22 |  |  | 5 | 13.67 |  | 5 | 31.42 |
|  |  | **4** | **15.40** |  | **4** | **-50.90** |  |  | 4 | 9.75 |  | **4** | **27.46** |
|  |  | 3 | 23.49 |  | 3 | -35.10 |  |  | **3** | **8.78** |  | 3 | 29.99 |
| 1930s* | 55 | 6 | 8.41 | 55 | 6 | -29.37 | 1890s* | 78 | 6 | 95.92 | 78 | 6 | 17.88 |
|  |  | 5 | 4.63 |  | 5 | -33.77 |  |  | 5 | 92.20 |  | 5 | 14.64 |
|  |  | **4** | **1.92** |  | **4** | **-37.36** |  |  | **4** | **89.49** |  | 4 | 11.24 |
|  |  | 3 | 11.23 |  | 3 | -14.66 |  |  | 3 | 91.46 |  | **3** | **7.18** |
| 1970s* | 70 | 6 | -17.05 | 70 | 6 | -65.58 | Contemporary^2^* | 56 | 6 | 51.05 | 56 | 6 | -8.54 |
|  |  | 5 | -19.49 |  | 5 | -68.82 |  |  | 5 | 47.44 |  | 5 | -12.12 |
|  |  | 4 | -19.44 |  | **4** | **-69.53** |  |  | 4 | 43.66 |  | 4 | -15.80 |
|  |  | **3** | **-21.09** |  | 3 | -25.82 |  |  | **3** | **39.98** |  | **3** | **-19.81** |
| 2000s* | 75 | 6 | 55.82 | 75 | 6 | -58.44 | Subsection 1 | 29 | 6 | 77.26 | 29 | 6 | 53.10 |
|  |  | 5 | 52.86 |  | 5 | -62.00 |  |  | 5 | 77.85 |  | 5 | 49.78 |
|  |  | **4** | **48.69** |  | **4** | **-66.30** |  |  | 4 | 74.80 |  | **4** | **47.69** |
|  |  | 3 | 51.12 |  | 3 | -33.76 |  |  | **3** | **72.00** |  | 3 | 48.91 |
| 2010s* | 78 | 6 | 71.71 | 78 | 6 | 14.94 | Subsection 8 | 31 | 6 | 64.31 | 31 | 6 | 30.60 |
|  |  | 5 | 68.34 |  | 5 | 11.38 |  |  | **5** | **61.10** |  | **5** | **27.39** |
|  |  | 4 | 64.72 |  | 4 | 9.55 |  |  | 4 | 65.27 |  | 4 | 34.95 |
|  |  | **3** | **61.54** |  | **3** | **7.91** |  |  | 3 | 78.76 |  | 3 | 39.02 |
| Contemporary^1^* | 89 | 6 | 106.73 | 89 | 6 | 36.50 | *s* | 31 | 6 | -13.29 | 31 | 6 | -56.99 |
|  |  | 5 | 103.35 |  | 5 | 33.04 |  |  | **5** | **-16.51** |  | 5 | -60.20 |
|  |  | 4 | 100.15 |  | 4 | 29.58 |  |  | 4 | 10.38 |  | 4 | -61.13 |
|  |  | **3** | **96.67** |  | **3** | **28.35** |  |  | 3 | 7.65 |  | **3** | **-62.85** |
| Subsection 1 | 36 | 6 | 79.38 | 54 | 6 | 45.22 |  | | | | | | |
|  |  | 5 | 76.09 |  | 5 | 44.51 |  |  |  |  |  |  |  |
|  |  | **4** | **73.44** |  | 4 | 42.03 |  |  |  |  |  |  |  |
|  |  | 3 | 88.48 |  | **3** | **39.64** |  |  |  |  |  |  |  |
| Subsection 8 | 37 | 6 | 69.06 | 56 | 6 | 32.09 |  |  |  |  |  |  |  |
|  |  | 5 | 65.73 |  | 5 | 29.42 |  |  |  |  |  |  |  |
|  |  | **4** | **65.29** |  | **4** | **27.22** |  |  |  |  |  |  |  |
|  |  | 3 | 77.00 |  | 3 | 34.72 |  |  |  |  |  |  |  |
| *s* | 58 | 6 | -45.74 | 58 | 6 | -103.53 |  |  |  |  |  |  |  |
|  |  | **5** | **-48.25** |  | **5** | **-106.75** |  |  |  |  |  |  |  |
|  |  | 4 | -31.31 |  | 4 | -89.30 |  |  |  |  |  |  |  |
|  |  | 3 | -26.20 |  | 3 | -91.73 |  |  |  |  |  |  |  |

^1^2016, 2017 and 2019; published in Golikov *et al.* 2022 and reused in this study;

^2^2016–2018 and 2023.

Table S4. Outputs of the residual analyses for the chosen *k* (the number of knots) for Generalized Additive Models (GAMs) used for temporal trends and for Generalized Additive Mixed Effect Models (GAMMs) used for ontogenetic trends. GAMMs marked with an asterisk (*). TP – trophic position, *s* – specialization index, *n* – sample size, edf – effective degrees of freedom. Significant *p*-values are in **bold**

| Model (GAM or  GAMM) | | *Gonatus fabricii* | | | | | | Model (GAM or  GAMM) | | *Todarodes sagittatus* | | | | | |
| --- | --- | --- | --- | --- | --- | --- | --- | --- | --- | --- | --- | --- | --- | --- | --- |
|  |  | *n* | *k* | Residual analyses | | | |  |  | *n* | *k* | Residual analyses | | | |
|  |  |  |  | *k*’ | edf | *k*-index | *p* |  |  |  |  | *k*’ | edf | *k*-index | *p* |
| Late XIXth century* | *δ*^13^C | 16 | 4 | 3.00 | 2.99 | 0.99 | 0.41 | 1840s* | *δ*^13^C | 32 | 3 | 2.00 | 1.00 | 1.29 | 0.94 |
|  | TP | 16 | 4 | 3.00 | 2.89 | 1.05 | 0.43 |  | TP | 32 | 3 | 2.00 | 1.89 | 1.32 | 0.94 |
| 1900s* | *δ*^13^C | 66 | 4 | 3.00 | 2.91 | 1.24 | 0.96 | 1880s* | *δ*^13^C | 80 | 3 | 2.00 | 1.99 | 0.88 | 0.16 |
|  | TP | 66 | 4 | 3.00 | 2.93 | 0.79 | 0.06 |  | TP | 80 | 4 | 3.00 | 2.85 | 0.98 | 0.40 |
| 1930s* | *δ*^13^C | 55 | 4 | 3.00 | 2.93 | 0.88 | 0.16 | 1890s* | *δ*^13^C | 78 | 4 | 3.00 | 2.79 | 0.87 | 0.09 |
|  | TP | 55 | 4 | 3.00 | 2.96 | 0.97 | 0.44 |  | TP | 78 | 3 | 2.00 | 1.95 | 1.05 | 0.58 |
| 1970s* | *δ*^13^C | 70 | 3 | 2.00 | 2.00 | 0.85 | 0.09 | Contemporary^2^* | *δ*^13^C | 56 | 3 | 2.00 | 1.97 | 0.89 | 0.13 |
|  | TP | 70 | 4 | 3.00 | 2.98 | 0.85 | 0.08 |  | TP | 56 | 3 | 2.00 | 1.96 | 1.12 | 0.77 |
| 2000s* | *δ*^13^C | 75 | 4 | 3.00 | 2.85 | 1.04 | 0.57 | *δ*^13^C | Subsection 1 | 29 | 3 | 2.00 | 1.00 | 0.80 | 0.10 |
|  | TP | 75 | 4 | 3.00 | 2.97 | 1.06 | 0.73 |  | Subsection 8 | 31 | 5 | 4.00 | 3.55 | 1.00 | 0.40 |
| 2010s* | *δ*^13^C | 78 | 3 | 2.00 | 1.97 | 1.10 | 0.78 | TP | Subsection 1 | 29 | 4 | 3.00 | 2.83 | 1.03 | 0.50 |
|  | TP | 78 | 3 | 2.00 | 1.00 | 0.97 | 0.35 |  | Subsection 8 | 31 | 5 | 4.00 | 3.48 | 1.26 | 0.90 |
| Contemporary^1^* | *δ*^13^C | 89 | 3 | 2.00 | 1.93 | 1.01 | 0.48 | *s* | *δ*^13^C | 31 | 5 | 4.00 | 3.79 | 1.11 | 0.70 |
|  | TP | 89 | 3 | 2.00 | 1.00 | 0.72 | **0.0050** |  | TP | 31 | 3 | 2.00 | 1.93 | 0.97 | 0.34 |
| *δ*^13^C | Subsection 1 | 36 | 4 | 3.00 | 2.95 | 0.88 | 0.17 |  | | | | | | | |
|  | Subsection 8 | 37 | 4 | 3.00 | 2.94 | 1.23 | 0.90 |  |  |  |  |  |  |  |  |
| TP | Subsection 1 | 54 | 3 | 2.00 | 1.00 | 0.79 | **0.0450** |  |  |  |  |  |  |  |  |
|  | Subsection 8 | 56 | 4 | 3.00 | 2.91 | 0.86 | 0.11 |  |  |  |  |  |  |  |  |
| *s* | *δ*^13^C | 58 | 5 | 4.00 | 3.93 | 0.97 | 0.35 |  |  |  |  |  |  |  |  |
|  | TP | 58 | 5 | 4.00 | 3.94 | 0.96 | 0.29 |  |  |  |  |  |  |  |  |

^1^2016, 2017 and 2019; published in Golikov *et al.* 2022 and reused in this study;

^2^2016–2018 and 2023.

Table S5. Temporal differences in *δ*^13^C values, Trophic Position (TP) and Specialization index (*s*) of *Gonatus fabricii*. Kruskal–Wallis *H* and Dunn’s *Z* tests are provided in the table. *n* – sample size, n/a – not applicable. Significant *p*-values are in **bold**

| Parameter | *δ*^13^C values, subsection 1^1^:  *d* = 1.79, *H*_4,35_ = 17.21, *p* = **0.0006** | | | | | | TP, subsection 1:  *d* = 1.45, *H*_6,53_ = 21.19, *p* = **0.0007** | | | | | | *s*, *δ*^13^C values:  *d* = 1.99, *H*_6,57_ = 30.31, *p* **< 0.0001** | | | | | |
| --- | --- | --- | --- | --- | --- | --- | --- | --- | --- | --- | --- | --- | --- | --- | --- | --- | --- | --- |
|  | 1900s^2^ | 1930s | 1970s | 2000s | 2010s^2^ | Contem-porary^2^ | 1900s | 1930s | 1970s | 2000s | 2010s | Contem-porary^2^ | 1900s | 1930s | 1970s | 2000s | 2010s | Contem-porary^2^ |
| *n* | 8 | 6 | 9 | 10 | 10 | 11 | 8 | 6 | 9 | 10 | 10 | 11 | 9 | 7 | 9 | 10 | 10 | 13 |
| Mean ± SD | -18.26  ± 0.19 | -17.59  ± 0.10 | -17.88  ± 0.12 | -16.78  ± 0.19 | -17.13  ± 0.13 | -17.98  ± 0.16 | 3.71  ± 0.06 | 3.92  ± 0.08 | 3.56  ± 0.05 | 3.50  ± 0.09 | 3.28  ± 0.08 | 3.76  ± 0.11 | 0.28  ± 0.04 | 0.55  ± 0.06 | 0.50  ± 0.03 | 0.26  ± 0.03 | 0.38  ± 0.03 | 0.56  ± 0.05 |
| 1900s | n/a | n/a | n/a | n/a | n/a | n/a | – | *d* = 0.67,  *Z* = 1.19 | *d* = 0.56,  *Z* = 1.11 | *d* = 0.83,  *Z* = 1.63 | *d* = 1.89,  *Z* = 2.91 | *d* = 0.06,  *Z* = 0.12 | – | *d* = 2.34,  *Z* = 3.04 | *d* = 1.89,  *Z* = 2.91 | *d* = 0.20,  *Z* = 0.44 | *d* = 0.55,  *Z* = 1.15 | *d* = 2.32,  *Z* = 3.55 |
| 1930s | n/a | – | *d* = 0.66,  *Z* = 1.22 | *d* = 0.89,  *Z* = 1.62 | n/a | *d* = 0.88,  *Z* = 1.66 | *p* = 0.23 | – | *d* = 1.43,  *Z* = 2.25 | *d* = 1.88,  *Z* = 2.74 | *d* = 9.85,  *Z* = 3.92 | *d* = 0.59,  *Z* = 1.16 | *p* = **0.0024** | – | *d* = 0.16,  *Z* = 0.32 | *d* = 3.28,  *Z* = 3.52 | *d* = 1.13,  *Z* = 2.03 | *d* = 0.01,  *Z* = 0.02 |
| 1970s | n/a | *p* = 0.23 | – | *d* = 2.16,  *Z* = 3.20 | n/a | *d* = 0.21,  *Z* = 0.47 | *p* = 0.27 | *p* = **0.0248** | – | *d* = 0.23,  *Z* = 0.50 | *d* = 0.93,  *Z* = 1.83 | *d* = 0.62,  *Z* = 1.32 | *p* **= 0.0036** | *p* = 0.75 | – | *d* = 2.55,  *Z* = 3.43 | *d* = 0.93,  *Z* = 1.83 | *d* = 0.17,  *Z* = 0.39 |
| 2000s | n/a | *p* = 0.10 | *p* = **0.0014** | – | n/a | *d* = 3.10,  *Z* = 3.85 | *p* = 0.10 | *p* = **0.0062** | *p* = 0.62 | – | *d* = 0.63,  *Z* = 1.34 | *d* = 0.91,  *Z* = 1.89 | *p* = 0.66 | *p* = **0.0004** | *p* = **0.0006** | – | *d* = 0.79,  *Z* = 1.64 | *d* = 3.42,  *Z* = 4.14 |
| 2010s | n/a | n/a | n/a | n/a | n/a | n/a | *p* = **0.0037** | *p* **< 0.0001** | *p* = 0.07 | *p* = 0.18 | – | *d* = 2.05,  *Z* = 3.28 | *p* = 0.25 | *p* = **0.0421** | *p* = 0.07 | *p* = 0.10 | – | *d* = 1.16,  *Z* = 2.40 |
| Contemporary^2^ | n/a | *p* = 0.10 | *p* = 0.64 | *p* = **0.0001** | n/a | – | *p* = 0.91 | *p* = 0.25 | *p* = 0.19 | *p* = 0.0590 | *p* = **0.0011** | – | *p* = **0.0004** | *p* = 0.99 | *p* = 0.70 | *p* **< 0.0001** | *p* = **0.0163** | – |
| Parameter | *δ*^13^C values, subsection 8^1^:  *d* = 1.45, *H*_4,36_ = 14.35, *p* = **0.0025** | | | | | | TP, subsection 8:  *d* = 1.53, *H*_6,55_ = 23.50, *p* = **0.0003** | | | | | | *s*, TP:  *d* = 5.74, *H*_6,57_ = 51.37, *p* **< 0.0001** | | | | | |
| *n* | 9 | 7 | 9 | 10 | 10 | 11 | 9 | 7 | 9 | 10 | 10 | 11 | 9 | 7 | 9 | 10 | 10 | 13 |
| Mean ± SD | -17.79  ± 0.19 | -16.57  ± 0.13 | -17.12  ± 0.13 | -16.07  ± 0.19 | -16.33  ± 0.14 | -16.76  ± 0.09 | 4.83  ± 0.04 | 5.18  ± 0.06 | 4.72  ± 0.06 | 4.64  ± 0.09 | 4.50  ± 0.09 | 4.97  ± 0.11 | 0.95  ± 0.01 | 0.93  ± 0.01 | 0.89  ± 0.01 | 0.61  ± 0.02 | 0.79  ± 0.01 | 0.69  ± 0.04 |
| 1900s | n/a | n/a | n/a | n/a | n/a | n/a | – | *d* = 0.89,  *Z* = 1.62 | *d* = 0.55,  *Z* = 1.13 | *d* = 1.06,  *Z* = 2.04 | *d* = 1.59,  *Z* = 2.71 | *d* = 0.03,  *Z* = 0.06 | – | *d* = 0.32,  *Z* = 0.64 | *d* = 1.03,  *Z* = 1.94 | *d* = 31.25,  *Z* = 5.90 | *d* = 2.49,  *Z* = 3.40 | *d* = 150.20,  *Z* = 4.83 |
| 1930s | n/a | – | *d* = 1.21,  *Z* = 2.07 | *d* = 0.68,  *Z* = 1.33 | n/a | *d* = 0.38,  *Z* = 0.80 | *p* = 0.11 | – | *d* = 1.81,  *Z* = 2.68 | *d* = 3.42,  *Z* = 3.56 | *d* = 51.14,  *Z* = 4.19 | *d* = 0.91,  *Z* = 1.75 | *p* = 0.52 | – | *d* = 0.62,  *Z* = 1.18 | *d* = 51.50,  *Z* = 4.84 | *d* = 1.54,  *Z* = 2.51 | *d* = 3.16,  *Z* = 3.78 |
| 1970s | n/a | *p* = **0.0384** | – | *d* = 3.18,  *Z* = 3.69 | n/a | *d* = 0.70,  *Z* = 1.47 | *p* = 0.26 | *p* = **0.0074** | – | *d* = 0.41,  *Z* = 0.88 | *d* = 0.77,  *Z* = 1.56 | *d* = 0.52,  *Z* = 1.12 | *p* = 0.0520 | *p* = 0.24 | – | *d* = 4.01,  *Z* = 3.90 | *d* = 0.68,  *Z* = 1.41 | *d* = 1.42,  *Z* = 2.72 |
| 2000s | n/a | *p* = 0.19 | *p* = **0.0002** | – | n/a | *d* = 1.22,  *Z* = 2.38 | *p* = **0.0416** | *p* = **0.0004** | *p* = 0.38 | – | *d* = 0.31,  *Z* = 0.69 | *d* = 1.02,  *Z* = 2.08 | *p* **< 0.0001** | *p* **< 0.0001** | *p* **< 0.0001** | – | *d* = 1.40,  *Z* = 2.57 | *d* = 0.34,  *Z* = 1.46 |
| 2010s | n/a | n/a | n/a | n/a | n/a | n/a | *p* = **0.0067** | *p* **< 0.0001** | *p* = 0.12 | *p* = 0.49 | – | *d* = 1.54,  *Z* = 2.79 | *p* = **0.0007** | *p* = **0.0120** | *p* = 0.16 | *p* = **0.0103** | – | *d* = 0.55,  *Z* = 1.27 |
| Contemporary^2^ | n/a | *p* = 0.43 | *p* = 0.14 | *p* = **0.0175** | n/a | – | *p* = 0.95 | *p* = 0.08 | *p* = 0.26 | *p* = **0.0374** | *p* = **0.0053** | – | *p* **< 0.0001** | *p* = **0.0002** | *p* = **0.0066** | *p* = 0.14 | *p* = 0.21 | – |

^1^Baffin Bay data only, as *δ*^13^C values in *G. fabricii* from the Baffin Bay and Nordic Seas are proven to be significantly different (Golikov *et al.* 2018). Mann–Whitney *U* test for the Nordic Seas (1900s vs. 2010s) is as follows: subsection 1, *d* = 3.07, *U* = 0.0, *p* = **0.0005** and subsection 8, *d* = 3.13, *U* = 0.0 and *p* = **0.0003**;

^2^2016, 2017 and 2019; published in Golikov *et al.* 2022 and reused in this study.

Table S6. Isotopic niche width for different upper beak subsections of *Gonatus fabricii* and *Todarodes sagittatus*. Values are mean ± SD. *n* – sample size

| Sub-  section | *Gonatus fabricii* | | | | | | | | | | | | *Todarodes sagittatus* | | | | | | | |
| --- | --- | --- | --- | --- | --- | --- | --- | --- | --- | --- | --- | --- | --- | --- | --- | --- | --- | --- | --- | --- |
|  | 1900s | | 1930s | | 1970s | | 2000s | | 2010s | | Contemporary^1^ | | 1840s | | 1880s | | 1890s | | Contemporary^2^ | |
|  | *n* | mean ± SD | *n* | mean ± SD | *n* | mean ± SD | *n* | mean ± SD | *n* | mean ± SD | *n* | mean ± SD | *n* | mean ± SD | *n* | mean ± SD | *n* | mean ± SD | *n* | mean ± SD |
| 1 | 8 | 1.31 ± 0.51 | 6 | 0.35 ± 0.15 | 9 | 0.86 ± 0.28 | 10 | 2.93 ± 0.94 | 10 | 1.56 ± 0.52 | 11 | 3.26 ± 1.00 | 4 | 2.26 ± 1.32 | 10 | 1.20 ± 0.43 | 8 | 3.10 ± 1.18 | 7 | 7.61 ± 3.23 |
| 2 | 7 | 0.78 ± 0.31 | 7 | 0.80 ± 0.32 | 7 | 0.79 ± 0.32 | 8 | 2.86 ± 1.11 | 10 | 1.90 ± 0.68 | 9 | 3.52 ± 1.21 | 4 | 1.45 ± 0.84 | 10 | 1.12 ± 0.37 | 10 | 3.20 ± 1.10 | 7 | 4.40 ± 1.89 |
| 3 | 8 | 2.15 ± 0.83 | 7 | 0.90 ±0.37 | 9 | 0.98 ± 0.34 | 8 | 2.35 ± 0.88 | 9 | 2.97 ± 1.02 | 11 | 4.40 ± 1.39 | 4 | 1.31 ± 0.79 | 10 | 1.41 ± 0.48 | 10 | 1.45 ± 0.49 | 7 | 3.23 ± 1.28 |
| 4 | 8 | 1.48 ± 0.62 | 7 | 1.21 ± 0.51 | 9 | 1.35 ± 0.48 | 9 | 3.19 ± 1.09 | 10 | 2.64 ± 0.89 | 13 | 4.81 ± 1.43 | 4 | 2.09 ± 1.38 | 10 | 0.55 ± 0.19 | 10 | 2.65 ± 0.88 | 7 | 2.32 ± 0.89 |
| 5 | 8 | 0.80 ± 0.30 | 7 | 0.63 ± 0.25 | 9 | 1.11 ± 0.41 | 10 | 3.38 ± 1.13 | 10 | 3.16 ± 1.08 | 12 | 2.74 ± 0.84 | 4 | 2.00 ± 1.13 | 10 | 0.54 ± 0.18 | 10 | 0.80 ± 0.26 | 7 | 2.24 ± 0.93 |
| 6 | 9 | 0.53 ± 0.18 | 7 | 0.77 ± 0.30 | 9 | 0.89 ± 0.33 | 10 | 3.34 ± 1.11 | 9 | 3.27 ± 1.17 | 11 | 3.70 ± 1.15 | 4 | 0.91 ± 0.50 | 10 | 0.59 ± 0.20 | 10 | 1.09 ± 0.37 | 7 | 1.33 ± 0.53 |
| 7 | 9 | 0.50 ± 0.18 | 7 | 1.37 ± 0.52 | 9 | 1.13 ± 0.41 | 10 | 2.81 ± 0.93 | 10 | 1.69 ± 0.57 | 13 | 1.93 ± 0.57 | 4 | 1.51 ± 1.02 | 10 | 0.81 ± 0.25 | 10 | 1.15 ± 0.38 | 7 | 1.50 ± 0.57 |
| 8 | 9 | 1.02 ± 0.36 | 7 | 0.64 ± 0.26 | 9 | 1.19 ± 0.43 | 10 | 2.88 ± 0.91 | 10 | 1.39 ± 0.49 | 11 | 1.56 ± 0.54 | 4 | 1.20 ± 0.68 | 10 | 1.69 ± 0.58 | 10 | 1.97 ± 0.69 | 7 | 1.83 ± 0.70 |
| 9 | – | – | 7 | 0.96 ± 0.38 | 9 | 1.20 ± 0.41 | 10 | 3.37 ± 1.14 | 10 | 1.64 ± 0.54 | 12 | 1.77± 0.53 | 4 | 0.10 ± 0.06 | 10 | 0.63 ± 0.21 | 10 | 1.40 ± 0.46 | 7 | 1.84 ± 0.70 |
| 10 | – | – | 7 | 0.61 ± 0.24 | 9 | 1.36 ± 0.51 | 10 | 3.20 ± 1.04 | 10 | 1.50 ± 0.51 | 10 | 1.18 ± 0.39 | 4 | 0.32 ± 0.18 | 10 | 0.78 ± 0.26 | 10 | 1.47 ± 0.46 | 6 | 2.20 ± 0.91 |
| 11 | – | – | – | – | 7 | 0.84 ± 0.32 | 10 | 3.36 ± 1.12 | 9 | 1.84 ± 0.65 | 11 | 1.27 ± 0.37 | 4 | 0.63 ± 0.40 | 10 | 0.33 ± 0.11 | 10 | 1.46 ± 0.47 | 5 | 1.93 ± 0.98 |
| 12 | – | – | – | – | 6 | 0.69 ± 0.29 | – | – | 10 | 2.07 ± 0.67 | 9 | 2.27 ± 0.82 | 4 | 0.80 ± 0.48 | 10 | 0.66 ± 0.23 | 10 | 1.23 ± 0.42 | 6 | 1.82 ± 0.79 |
| 13 | – | – | – | – | – | – | – | – | 10 | 2.17 ± 0.73 | 10 | 3.48 ± 1.12 | – | – | 5 | 0.35 ± 0.17 | 10 | 2.08 ± 0.70 | 6 | 2.20 ± 1.00 |
| 14 | – | – | – | – | – | – | – | – | 10 | 1.98 ± 0.66 | 8 | 2.96 ± 1.16 | – | – | 5 | 0.48 ± 0.26 | 9 | 1.58 ± 0.53 | 6 | 2.16 ± 0.92 |
| 15 | – | – | – | – | – | – | – | – | 10 | 1.96 ± 0.69 | – | – | – | – | – | – | 10 | 1.25 ± 0.40 | 5 | 2.29 ± 1.02 |
| 16 | – | – | – | – | – | – | – | – | – | – | – | – | – | – | – | – | 6 | 1.05 ± 0.47 | – | – |
| 17 | – | – | – | – | – | – | – | – | – | – | – | – | – | – | – | – | 6 | 1.27 ± 0.57 | – | – |

^1^2016, 2017 and 2019; published in Golikov *et al.* 2022 and reused in this study;

^2^2016–2018 and 2023.

Table S7. Overlap among isotopic niches for similar upper beak subsections from different time series in *Gonatus fabricii* and *Todarodes sagittatus*. *n* – sample size. Large overlap values (treated as significant overlap) are in **bold**

| Subsections | *Gonatus fabricii*, Baffin Bay: subsection 1 | | | | | *Gonatus fabricii*, Baffin Bay: subsection 8 | | | | |
| --- | --- | --- | --- | --- | --- | --- | --- | --- | --- | --- |
|  | *n* | 1930s | 1970s | 2000s | Contemporary^1^ | *n* | 1930s | 1970s | 2000s | Contemporary^1^ |
| 1930s | 6 | – | 49.25 | **75.70** | **99.25** | 7 | – | 45.10 | **83.42** | **82.56** |
| 1970s | 9 | 35.38 | – | **81.52** | **98.69** | 9 | 29.19 | – | **84.86** | **74.76** |
| 2000s | 10 | 4.14 | 27.55 | – | 51.97 | 10 | 12.31 | 40.06 | – | 53.42 |
| Contemporary^1^ | 11 | 26.26 | 51.89 | 53.01 | – | 11 | 46.25 | **71.78** | **89.17** | – |
| Subsections | *Gonatus fabricii*, Nordic Seas: subsection 1 | | | | | *Gonatus fabricii*, Nordic Seas: subsection 8 | | | | |
|  | *n* | 1900s | | 2010s | | *n* | 1900s | | 2010s | |
| 1900s | 8 | – | | 40.17 | | 9 | – | | 22.18 | |
| 2010s | 10 | 16.64 | | – | | 10 | 27.63 | | – | |
| Subsections | *Todarodes sagittatus*: subsection 1 | | | | | *Todarodes sagittatus*: subsection 8 | | | | |
|  | *n* | 1840s | 1880s | 1890s | Contemporary^2^ | *n* | 1840s | 1880s | 1890s | Contemporary^2^ |
| 1840s | 4 | – | **62.04** | 29.10 | **75.71** | 4 | – | 2.90 | 18.01 | 5.66 |
| 1880s | 10 | **86.14** | – | 39.28 | **92.77** | 10 | 1.24 | – | 26.38 | **74.06** |
| 1890s | 8 | 26.82 | 10.20 | – | **79.70** | 10 | 7.73 | 31.29 | – | 9.65 |
| Contemporary^2^ | 7 | 15.83 | 18.33 | 46.76 | – | 7 | 1.48 | **73.98** | 9.89 | – |

^1^2016, 2017 and 2019; published in Golikov *et al.* 2022 and reused in this study;

^2^2016–2018 and 2023.

Table S8. Spearman’s rank correlation between *δ*^13^C and *δ*^15^N values within each studied individual of *Gonatus fabricii* and *Todarodes sagittatus*. *n* – sample size, n/a – not applicable. Significant overlap values are in **bold**

| Individual | *Gonatus fabricii* | | | | | | | *Todarodes sagittatus* | | | |
| --- | --- | --- | --- | --- | --- | --- | --- | --- | --- | --- | --- |
|  | Late XIXth  century | 1900s | 1930s | 1970s | 2000s | 2010s | Contemporary^1^ | 1840s | 1880s | 1890s | Contemporary^2^ |
| 1 | *n* = 17,  *r* = 0.48,  *p* = 0.08 | *n* = 10,  *r* = -0.30,  *p* = 0.40 | *n* = 18,  *r* = 0.14,  *p* = 0.36 | *n* = 10,  *r* = 0.27,  *p* = 0.46 | *n* = 13,  *r* = 0.69,  *p* = **0.0087** | *n* = 16,  Pearson *r* = 0.75,  *p* = **0.0009** | *n* = 13,  *r* = 0.65,  *p* = **0.0165** | *n* = 12,  *r* = 0.67,  *p* = **0.0168** | *n* = 14,  *r* = 0.51,  *p* = 0.06 | *n* = 20,  *r* = -0.15,  *p* = 0.53 | *n* = 10,  *r* = 0.75,  *p* = **0.0133** |
| 2 | *n* = 15,  *r* = 0.21,  *p* = 0.45 | *n* = 8,  *r* = 0.25,  *p* = 0.55 | *n* = 18,  *r* = 0.26,  *p* = 0.31 | *n* = 9,  *r* = 0.48,  *p* = 0.20 | *n* = 14,  *r* = 0.30,  *p* = 0.30 | *n* = 15,  Pearson *r* = 0.69,  *p* = **0.0045** | *n* = 13,  *r* = 0.88,  *p* **< 0.0001** | *n* = 12,  *r* = 0.55,  *p* = 0.07 | *n* = 15,  *r* = 0.91,  *p* **< 0.0001** | *n* = 19,  *r* = 0.17,  *p* = 0.50 | *n* = 15,  *r* = 0.97,  *p* **< 0.0001** |
| 3 | – | *n* = 6,  *r* = -0.03,  *p* = 0.99 | *n* = 10,  *r* = 0.02,  *p* = 0.96 | *n* = 12,  *r* = 0.41,  *p* = 0.19 | *n* = 12,  *r* = 0.41,  *p* = 0.19 | *n* = 15,  Pearson *r* = 0.21,  *p* = 0.45 | n/a | *n* = 15,  *r* = 0.73,  *p* = **0.0021** | *n* = 14,  *r* = 0.53,  *p* = 0.0514 | *n* = 18,  *r* = -0.10,  *p* = 0.71 | *n* = 19,  *r* = -037,  *p* = 0.17 |
| 4 | – | *n* = 7,  *r* = -0.29,  *p* = 0.56 | *n* = 12,  *r* = -0.10,  *p* = 0.76 | *n* = 12,  *r* = 0.03,  *p* = 0.93 | *n* = 14,  *r* = 0.20,  *p* = 0.50 | *n* = 14,  Pearson *r* = 0.62,  *p* = **0.0179** | n/a | *n* = 12,  *r* = 0.80,  *p* = **0.0020** | *n* = 14,  *r* = 0.41,  *p* = 0.14 | *n* = 15,  *r* = 0.29,  *p* = 0.30 | *n* = 21,  *r* = 0.98,  *p* **< 0.0001** |
| 5 | – | *n* = 6,  *r* = 0.03,  *p* = 0.98 | *n* = 12,  *r* = 0.34,  *p* = 0.29 | *n* = 12,  *r* = 0.28,  *p* = 0.39 | *n* = 14,  *r* = 0.07,  *p* = 0.83 | *n* = 14,  Pearson *r* = 0.50,  *p* = 0.07 | n/a | – | *n* = 12,  *r* = 0.83,  *p* = **0.0010** | *n* = 17,  *r* = -0.38,  *p* = 0.14 | *n* = 23,  *r* = 0.90,  *p* **< 0.0001** |
| 6 | – | *n* = 7,  *r* = 0.32,  *p* = 0.47 | *n* = 11,  *r* = 0.19,  *p* = 0.58 | *n* = 12,  *r* = 0.14,  *p* = 0.65 | *n* = 11,  *r* = 0.15,  *p* = 0.67 | *n* = 15,  Pearson *r* = 0.31,  *p* = 0.26 | *n* = 15,  *r* = 0.75,  *p* = **0.0014** | – | *n* = 12,  *r* = 0.92,  *p* **< 0.0001** | *n* = 16,  *r* = 0.24,  *p* = 0.38 | *n* = 22,  *r* = 0.96,  *p* **< 0.0001** |
| 7 | – | *n* = 8,  *r* = 0.33,  *p* = 0.41 | *n* = 11,  *r* = 0.19,  *p* = 0.57 | *n* = 12,  *r* = 0.28,  *p* = 0.38 | *n* = 10,  *r* = -0.22,  *p* = 0.53 | *n* = 16,  Pearson *r* = 0.26,  *p* = 0.33 | n/a | – | *n* = 14,  *r* = 0.56,  *p* = **0.0360** | *n* = 15,  *r* = -0.11,  *p* = 0.70 | *n* = 14,  *r* = 0.57,  *p* = **0.0336** |
| 8 | – | *n* = 8,  *r* = 0.36,  *p* = 0.38 | – | *n* = 11,  *r* = 0.51,  *p* = 0.11 | *n* = 11,  *r* = -0.39,  *p* = 0.23 | *n* = 15,  Pearson *r* = 0.66,  *p* = **0.0073** | *n* = 11,  *r* = 0.68,  *p* = **0.0208** | – | *n* = 12,  *r* = 0.78,  *p* = **0.0030** | *n* = 15,  *r* = 0.17,  *p* = 0.55 | – |
| 9 | – | *n* = 9,  *r* = 0.09,  *p* = 0.82 | – | *n* = 11,  *r* = 0.27,  *p* = 0.42 | *n* = 10,  *r* = -0.44,  *p* = 0.20 | *n* = 12,  Pearson *r* = 0.51,  *p* = **0.0498** | *n* = 14,  *r* = 0.77,  *p* = **0.0012** | – | *n* = 12,  *r* = 0.74,  *p* = **0.0057** | *n* = 17,  *r* = -0.38,  *p* = 0.14 | – |
| 10 | – | – | – | – | *n* = 9,  *r* = -0.40,  *p* = 0.29 | *n* = 12,  Pearson *r* = 0.56,  *p* = **0.0297** | n/a | – | *n* = 12,  *r* = 0.89,  *p* = **0.0001** | *n* = 15,  *r* = -0.24,  *p* = 0.38 | – |
| 11 | – | – | – | – | – | – | *n* = 14,  *r* = -0.03,  *p* = 0.83 | – | – | – | – |
| 12 | – | – | – | – | – | – | *n* = 15,  *r* = 0.73,  *p* = **0.0021** | – | – | – | – |
| 13 | – | – | – | – | – | – | n/a | – | – | – | – |

^1^2016, 2017 and 2019; published in Golikov *et al.* 2022 and reused in this study;

^2^2016–2018 and 2023.

Table S9. Temporal differences in *δ*^13^C values, Trophic Position (TP) and Specialization index (*s*) of *Todarodes sagittatus*. Kruskal–Wallis *H* and Dunn’s *Z* tests are provided in the table. *n* – sample size. Significant *p*-values are in **bold**

| Parameter | *δ*^13^C values, subsection 1^1^:  *d* = 1.83, *H*_4,28_ = 14.38, *p* = **0.0024** | | | | TP, subsection 1:  *d* = 2.07, *H*_4,28_ = 15.92, *p* = **0.0012** | | | | *s*, *δ*^13^C values:  *d* = 1.99, *H*_4,28_ = 30.31, *p* **< 0.0001** | | | |
| --- | --- | --- | --- | --- | --- | --- | --- | --- | --- | --- | --- | --- |
|  | 1840s | 1880s | 1890s | Contemporary^1^ | 1840s | 1880s | 1890s | Contemporary^1^ | 1840s | 1880s | 1890s | Contemporary^1^ |
| *n* | 4 | 10 | 8 | 7 | 4 | 10 | 8 | 7 | 4 | 10 | 10 | 7 |
| Mean ± SD | -18.29  ± 0.49 | -17.55  ± 0.12 | -18.13  ± 0.22 | -16.68 ± 0.27 | 2.30  ± 0.15 | 2.49  ± 0.07 | 3.17  ± 0.12 | 3.42 ± 0.24 | 0.43  ± 0.10 | 0.82  ± 0.03 | 0.41  ± 0.03 | 0.49 ± 0.06 |
| 1840s | – | *d* = 0.87,  *Z* = 1.49 | *d* = 0.01,  *Z* = 0.01 | *d* = 0.74,  *Z* = 2.85 | – | *d* = 0.28,  *Z* = 0.51 | *d* = 2.16,  *Z* = 2.54 | *d* = 3.55,  *Z* = 2.89 | – | *d* = 2.43,  *Z* = 2.89 | *d* = 0.08,  *Z* = 0.14 | *d* = 0.28,  *Z* = 0.34 |
| 1880s | *p* = 0.14 | – | *d* = 1.00,  *Z* = 1.89 | *d* = 0.99,  *Z* = 1.83 | *p* = 0.61 | – | *d* = 1.60,  *Z* = 2.65 | *d* = 2.22,  *Z* = 3.06 | *p* = **0.0038** | – | *d* = 4.05,  *Z* = 4.01 | *d* = 1.83,  *Z* = 2.78 |
| 1890s | *p* = 0.99 | *p* = 0.06 | – | *d* = 3.92,  *Z* = 3.45 | *p* = **0.0110** | *p* = **0.0081** | – | *d* = 0.26,  *Z* = 0.49 | *p* = 0.89 | *p* **< 0.0001** | – | *d* = 0.44,  *Z* = 0.89 |
| Contemporary^1^ | *p* = **0.0044** | *p* = 0.07 | *p* = **0.0006** | – | *p* = **0.0039** | *p* = **0.0022** | *p* = 0.63 | – | *p* = 0.57 | *p* = **0.0059** | *p* = 0.38 | – |
| Parameter | *δ*^13^C values, subsection 8^1^:  *d* = 1.04, *H*_4,30_ = 8.75, *p* = **0.0328** | | | | TP, subsection 8:  *d* = 1.87, *H*_4,30_ = 15.59, *p* = **0.0014** | | | | *s*, TP:  *d* = 5.74, *H*_4,30_ = 51.37, *p* **< 0.0001** | | | |
| *n* | 4 | 10 | 10 | 7 | 4 | 10 | 10 | 7 | 4 | 10 | 10 | 7 |
| Mean ± SD | -16.47  ± 0.25 | -15.94  ± 0.13 | -17.20  ± 0.18 | -15.31 ± 0.16 | 3.35  ± 0.09 | 4.49  ± 0.07 | 4.18  ± 0.07 | 4.43 ± 0.10 | 0.88  ± 0.02 | 0.97  ± 0.01 | 0.90  ± 0.02 | 0.58 ± 0.05 |
| 1840s | – | *d* = 0.30,  *Z* = 0.56 | *d* = 1.70,  *Z* = 2.42 | *d* = 1.09,  *Z* = 1.59 | – | *d* = 6.18,  *Z* = 3.56 | *d* = 1.24,  *Z* = 1.97 | *d* = 4.89,  *Z* = 3.07 | – | *d* = 1.81,  *Z* = 2.51 | *d* = 0.24,  *Z* = 0.44 | *d* = 1.05,  *Z* = 1.54 |
| 1880s | *p* = 0.58 | – | *d* = 1.30,  *Z* = 2.43 | *d* = 0.70,  *Z* = 1.36 | *p* = **0.0003** | – | *d* = 1.16,  *Z* = 2.24 | *d* = 0.20,  *Z* = 0.41 | *p* = **0.0122** | – | *d* = 1.54,  *Z* = 2.73 | *d* = 41.98,  *Z* = 4.97 |
| 1890s | *p* = **0.0154** | *p* = **0.0149** | – | *d* = 0.47,  *Z* = 0.94 | *p* = 0.06 | *p* = **0.0252** | – | *d* = 0.85,  *Z* = 1.61 | *p* = 0.66 | *p* = **0.0063** | – | *d* = 1.52,  *Z* = 2.49 |
| Contemporary^1^ | *p* = 0.11 | *p* = 0.17 | *p* = 0.17 | – | *p* = **0.0021** | *p* = 0.68 | *p* = 0.11 | – | *p* = 0.12 | *p* **< 0.0001** | *p* = **0.0128** | – |

^1^2016–2018 and 2023.

Table S10. Outputs of Generalized Additive Models (GAMs) of long-term trends in *δ*^13^C values, Trophic Position (TP) and Specialization index (*s*) of *Todarodes sagittatus*. In GAMs, *k* indicates the number of knots and *s* represents the smoother (year), where values are effective degrees of freedom. *n* – sample size. Significant *p*-values are in **bold**

| *δ*^13^C values | | | | | | | TP | | | | | | |
| --- | --- | --- | --- | --- | --- | --- | --- | --- | --- | --- | --- | --- | --- |
| Subsection 1 | | | | | | | Subsection 1 | | | | | | |
| Time | *n* | Intercept  (mean ± SE) | *k* | *s*(Year) | *p* | Deviance  explained, % | Time | *n* | Intercept  (mean ± SE) | *k* | *s*(Year) | *p* | Deviance  explained, % |
| 1844–2023 | 29 | -17.60 ± 0.12 | 3 | 1.00 | **0.0002** | 40.0 | 1844–2023 | 29 | 2.88 ± 0.08 | 4 | 2.83 | **0.0001** | 56.3 |
| Subsection 8 | | | | | | | Subsection 8 | | | | | | |
| 1844–2023 | 31 | -16.27 ± 0.08 | 5 | 3.55 | **<0.0001** | 75.0 | 1844–2023 | 31 | 4.21 ± 0.05 | 5 | 3.48 | **<0.0001** | 68.3 |
| *s* | | | | | | | *s* | | | | | | |
| 1844–2023 | 31 | 0.56 ± 0.02 | 5 | 3.79 | **<0.0001** | 69.1 | 1844–2023 | 31 | 0.85 ± 0.01 | 3 | 1.93 | **<0.0001** | 82.4 |

Table S11. Comparison of *δ*^13^C values among different upper beak subsections in *Gonatus fabricii*, using the Skillings–Mack test with the Nemenyi post hoc test. *n* – sample size. Significant *p*-values are in **bold**

| Subsections | 1900s: _sm_ = 42.37, d. f. = 7, *p* < **0.0001**  *n*: 9 individuals, 73 subsections | | | | | | | | | | | | | | | | | | | | | | | | | | | |
| --- | --- | --- | --- | --- | --- | --- | --- | --- | --- | --- | --- | --- | --- | --- | --- | --- | --- | --- | --- | --- | --- | --- | --- | --- | --- | --- | --- | --- |
|  | 1 (anterior) | | | | | 2 | | | | | 3 | | | | 4 | | | 5 | | | | 6 | | | 7 (posterior) | | | |
| 2 | 0.50 | | | | | – | | | | | – | | | | – | | | – | | | | – | | | – | | | |
| 3 | **0.0025** | | | | | 0.53 | | | | | – | | | | – | | | – | | | | – | | | – | | | |
| 4 | **0.0003** | | | | | 0.22 | | | | | 1.00 | | | | – | | | – | | | | – | | | – | | | |
| 5 | **0.0036** | | | | | 0.60 | | | | | 1.00 | | | | 1.00 | | | – | | | | – | | | – | | | |
| 6 | 0.24 | | | | | 1.00 | | | | | 0.81 | | | | 0.47 | | | 0.86 | | | | – | | | – | | | |
| 7 | 0.79 | | | | | 1.00 | | | | | 0.26 | | | | 0.08 | | | 0.32 | | | | 0.99 | | | – | | | |
| 8 | 0.99 | | | | | 0.94 | | | | | **0.0433** | | | | **0.0074** | | | 0.06 | | | | 0.76 | | | 1.00 | | | |
| 1930s: _sm_ = 40.87, d. f. = 9, *p* < **0.0001**  *n*: 7 individuals, 91 subsections | | | | | | | | | | | | | | | | | | | | | | | | | | | | |
| Subsections | 1 (anterior) | | 2 | | | 3 | | 4 | | | | 5 | | | 6 | | | 7 | | 8 | | | 9 (posterior) | | | | | |
| 2 | 1.00 | | – | | | – | | – | | | | – | | | – | | | – | | – | | | – | | | | | |
| 3 | 0.73 | | 0.55 | | | – | | – | | | | – | | | – | | | – | | – | | | – | | | | | |
| 4 | **0.0129** | | **0.0048** | | | 0.76 | | – | | | | – | | | – | | | – | | – | | | – | | | | | |
| 5 | **0.0029** | | **0.0009** | | | 0.48 | | 1.00 | | | | – | | | – | | | – | | – | | | – | | | | | |
| 6 | 0.06 | | **0.0275** | | | 0.96 | | 1.00 | | | | 1.00 | | | – | | | – | | – | | | – | | | | | |
| 7 | 0.39 | | 0.24 | | | 1.00 | | 0.96 | | | | 0.81 | | | 1.00 | | | – | | – | | | – | | | | | |
| 8 | 0.48 | | 0.31 | | | 1.00 | | 0.92 | | | | 0.73 | | | 1.00 | | | 1.00 | | – | | | – | | | | | |
| 9 | 0.83 | | 0.67 | | | 1.00 | | 0.64 | | | | 0.36 | | | 0.91 | | | 1.00 | | 1.00 | | | – | | | | | |
| 10 | 0.85 | | 0.70 | | | 1.00 | | 0.61 | | | | 0.34 | | | 0.89 | | | 1.00 | | 1.00 | | | 1.00 | | | | | |
| 1970s: _sm_ = 50.68, d. f. = 9, *p* < **0.0001**  *n*: 9 individuals, 101 subsections | | | | | | | | | | | | | | | | | | | | | | | | | | | | |
| Subsections | 1 (anterior) | | 2 | | | 3 | | 4 | | | | 5 | | | 6 | | | 7 | | 8 | | | 9 (posterior) | | | | | |
| 2 | 0.64 | | – | | | – | | – | | | | – | | | – | | | – | | – | | | – | | | | | |
| 3 | 0.15 | | 1.00 | | | – | | – | | | | – | | | – | | | – | | – | | | – | | | | | |
| 4 | **0.0002** | | 0.18 | | | 0.69 | | – | | | | – | | | – | | | – | | – | | | – | | | | | |
| 5 | **<0.0001** | | **0.0247** | | | 0.23 | | 1.00 | | | | – | | | – | | | – | | – | | | – | | | | | |
| 6 | **0.0003** | | 0.23 | | | 0.77 | | 1.00 | | | | 1.00 | | | – | | | – | | – | | | – | | | | | |
| 7 | **0.0281** | | 0.93 | | | 1.00 | | 0.96 | | | | 0.61 | | | 0.98 | | | – | | – | | | – | | | | | |
| 8 | 0.27 | | 1.00 | | | 1.00 | | 0.50 | | | | 0.12 | | | 0.58 | | | 1.00 | | – | | | – | | | | | |
| 9 | 0.08 | | 0.99 | | | 1.00 | | 0.93 | | | | 0.37 | | | 0.89 | | | 1.00 | | 1.00 | | | – | | | | | |
| 10 | **0.0166** | | 0.87 | | | 1.00 | | 0.98 | | | | 0.72 | | | 0.99 | | | 1.00 | | 0.99 | | | 1.00 | | | | | |
| 2000s: _sm_ = 57.26, d. f. = 10, *p* < **0.0001**  *n*: 10 individuals, 123 subsections | | | | | | | | | | | | | | | | | | | | | | | | | | | | |
| Subsections | 1 (anterior) | | | 2 | | 3 | | 4 | | | | 5 | | | 6 | | 7 | | 8 | | | 9 | | | 10 (posterior) | | | |
| 2 | 0.12 | | | – | | – | | – | | | | – | | | – | | – | | – | | | – | | | – | | | |
| 3 | **0.0015** | | | 0.97 | | – | | – | | | | – | | | – | | – | | – | | | – | | | – | | | |
| 4 | **<0.0001** | | | 0.09 | | 0.81 | | – | | | | – | | | – | | – | | – | | | – | | | – | | | |
| 5 | **<0.0001** | | | 0.56 | | 1.00 | | 1.00 | | | | – | | | – | | – | | – | | | – | | | – | | | |
| 6 | **0.0034** | | | 0.99 | | 1.00 | | 0.68 | | | | 0.99 | | | – | | – | | – | | | – | | | – | | | |
| 7 | **0.0011** | | | 0.96 | | 1.00 | | 0.84 | | | | 1.00 | | | 1.00 | | – | | – | | | – | | | – | | | |
| 8 | 0.12 | | | 1.00 | | 0.97 | | 0.09 | | | | 0.56 | | | 0.99 | | 0.96 | | – | | | – | | | – | | | |
| 9 | 0.73 | | | 1.00 | | 0.44 | | **0.0026** | | | | 0.06 | | | 0.59 | | 0.39 | | 1.00 | | | – | | | – | | | |
| 10 | 0.42 | | | 1.00 | | 0.75 | | **0.0138** | | | | 0.20 | | | 0.86 | | 0.70 | | 1.00 | | | 1.00 | | | – | | | |
| 11 | 0.49 | | | 1.00 | | 0.68 | | **0.0096** | | | | 0.16 | | | 0.81 | | 0.63 | | 1.00 | | | 1.00 | | | 1.00 | | | |
| 2010s: _sm_ = 78.45, d. f. = 14, *p* < **0.0001**  *n*: 13 individuals, 153 subsections | | | | | | | | | | | | | | | | | | | | | | | | | | | | |
| Subsections | 1 | 2 | | | 3 | | 4 | | | 5 | | | 6 | 7 | | 8 | | 9 | | | 10 | | | 11 | | 12 | 13 | 14 |
| 2 | 1.00 | – | | | – | | – | | | – | | | – | – | | – | | – | | | – | | | – | | – | – | – |
| 3 | 0.10 | 0.65 | | | – | | – | | | – | | | – | – | | – | | – | | | – | | | – | | – | – | – |
| 4 | **0.0076** | 0.16 | | | 1.00 | | – | | | – | | | – | – | | – | | – | | | – | | | – | | – | – | – |
| 5 | **0.0013** | 0.0569 | | | 1.00 | | 1.00 | | | – | | | – | – | | – | | – | | | – | | | – | | – | – | – |
| 6 | **0.0210** | 0.31 | | | 1.00 | | 1.00 | | | 1.00 | | | – | – | | – | | – | | | – | | | – | | – | – | – |
| 7 | **0.0022** | 0.22 | | | 1.00 | | 1.00 | | | 1.00 | | | 1.00 | – | | – | | – | | | – | | | – | | – | – | – |
| 8 | 0.25 | 0.88 | | | 1.00 | | 1.00 | | | 0.98 | | | 1.00 | 1.00 | | – | | – | | | – | | | – | | – | – | – |
| 9 | 0.46 | 0.97 | | | 1.00 | | 0.99 | | | 0.88 | | | 1.00 | 1.00 | | 1.00 | | – | | | – | | | – | | – | – | – |
| 10 | 0.90 | 1.00 | | | 0.99 | | 0.72 | | | 0.44 | | | 0.88 | 0.80 | | 1.00 | | 1.00 | | | – | | | – | | – | – | – |
| 11 | **0.0449** | 0.46 | | | 1.00 | | 1.00 | | | 1.00 | | | 1.00 | 1.00 | | 1.00 | | 1.00 | | | 0.95 | | | – | | – | – | – |
| 12 | **0.0449** | 0.46 | | | 1.00 | | 1.00 | | | 1.00 | | | 1.00 | 1.00 | | 1.00 | | 1.00 | | | 0.95 | | | 1.00 | | – | – | – |
| 13 | **0.0004** | **0.0210** | | | 0.99 | | 1.00 | | | 1.00 | | | 1.00 | 1.00 | | 0.90 | | 0.72 | | | 0.25 | | | 1.00 | | 1.00 | – | – |
| 14 | **<0.0001** | **0.0003** | | | 0.48 | | 0.93 | | | 1.00 | | | 0.81 | 0.89 | | 0.23 | | 0.10 | | | **0.0111** | | | 0.67 | | 0.67 | 1.00 | – |
| 15 | **<0.0001** | **<0.0001** | | | 0.11 | | 0.53 | | | 0.80 | | | 0.34 | 0.44 | | **0.0351** | | **0.0111** | | | **0.0007** | | | 0.21 | | 0.21 | 0.93 | 1.00 |
| Contemporary (2016, 2017, 2019)^1^: _sm_ = 55.76, d. f. = 10, *p* < **0.0001**  *n*: 10 individuals, 157 subsections | | | | | | | | | | | | | | | | | | | | | | | | | | | | |
| Subsections | 1 (anterior) | | | 2 | | 3 | | | 4 | | | 5 | | | 6 | | 7 | | 8 | | | 9 | | | 10 (posterior) | | | |
| 2 | 0.81 | | | – | | – | | | – | | | – | | | – | | – | | – | | | – | | | – | | | |
| 3 | 1.00 | | | 1.00 | | – | | | – | | | – | | | – | | – | | – | | | – | | | – | | | |
| 4 | 0.54 | | | 1.00 | | 0.99 | | | – | | | – | | | – | | – | | – | | | – | | | – | | | |
| 5 | **0.0451** | | | 0.92 | | 0.49 | | | 0.99 | | | – | | | – | | – | | – | | | – | | | – | | | |
| 6 | **0.0015** | | | 0.35 | | 0.06 | | | 0.64 | | | 1.00 | | | – | | – | | – | | | – | | | – | | | |
| 7 | **0.0020** | | | 0.40 | | 0.08 | | | 0.68 | | | 1.00 | | | 1.00 | | – | | – | | | – | | | – | | | |
| 8 | **0.0036** | | | 0.49 | | 0.12 | | | 0.78 | | | 1.00 | | | 1.00 | | 1.00 | | – | | | – | | | – | | | |
| 9 | **0.0138** | | | 0.74 | | 0.26 | | | 0.94 | | | 1.00 | | | 1.00 | | 1.00 | | 1.00 | | | – | | | – | | | |
| 10 | **0.0036** | | | 0.23 | | **0.0341** | | | 0.49 | | | 1.00 | | | 1.00 | | 1.00 | | 1.00 | | | 1.00 | | | – | | | |
| 11 | **0.0072** | | | 0.62 | | 0.18 | | | 0.87 | | | 1.00 | | | 1.00 | | 1.00 | | 1.00 | | | 1.00 | | | 1.00 | | | |

^1^published in Golikov *et al.* 2022 and reused in this study.

Table S12. Comparison of trophic position among different upper beak subsections in *Gonatus fabricii*, using the Skillings–Mack test with the Nemenyi post hoc test. *n* – sample size. Significant *p*-values are in **bold**

| Subsections | 1900s: _sm_ = 50.78, d. f. = 7, *p* < **0.0001**  *n*: 9 individuals, 73 subsections | | | | | | | | | | | | | | | | | | | | | | | | | | | | | | | | | | | |
| --- | --- | --- | --- | --- | --- | --- | --- | --- | --- | --- | --- | --- | --- | --- | --- | --- | --- | --- | --- | --- | --- | --- | --- | --- | --- | --- | --- | --- | --- | --- | --- | --- | --- | --- | --- | --- |
|  | 1 (anterior) | | | | | | 2 | | | | | | 3 | | | | 4 | | | | | | 5 | | | | | 6 | | | | 7 (posterior) | | | | |
| 2 | 0.94 | | | | | | – | | | | | | – | | | | – | | | | | | – | | | | | – | | | | – | | | | |
| 3 | 0.88 | | | | | | 1.00 | | | | | | – | | | | – | | | | | | – | | | | | – | | | | – | | | | |
| 4 | 0.60 | | | | | | 1.00 | | | | | | 1.00 | | | | – | | | | | | – | | | | | – | | | | – | | | | |
| 5 | **0.0433** | | | | | | 0.53 | | | | | | 0.67 | | | | 0.92 | | | | | | – | | | | | – | | | | – | | | | |
| 6 | **0.0126** | | | | | | 0.29 | | | | | | 0.40 | | | | 0.73 | | | | | | 1.00 | | | | | – | | | | – | | | | |
| 7 | **0.0009** | | | | | | 0.0575 | | | | | | 0.10 | | | | 0.29 | | | | | | 0.97 | | | | | 1.00 | | | | – | | | | |
| 8 | **0.0030** | | | | | | 0.12 | | | | | | 0.19 | | | | 0.47 | | | | | | 1.00 | | | | | 1.00 | | | | 1.00 | | | | |
| 1930s: _sm_ = 58.43, d. f. = 9, *p* < **0.0001**  *n*: 7 individuals, 91 subsections | | | | | | | | | | | | | | | | | | | | | | | | | | | | | | | | | | | | |
| Subsections | 1 (anterior) | | 2 | | | | 3 | | | 4 | | | | 5 | | | | 6 | | | | | 7 | | | 8 | | | 9 (posterior) | | | | | | | |
| 2 | 1.00 | | – | | | | – | | | – | | | | – | | | | – | | | | | – | | | – | | | – | | | | | | | |
| 3 | 1.00 | | 1.00 | | | | – | | | – | | | | – | | | | – | | | | | – | | | – | | | – | | | | | | | |
| 4 | 0.99 | | 0.92 | | | | 0.99 | | | – | | | | – | | | | – | | | | | – | | | – | | | – | | | | | | | |
| 5 | 0.92 | | 0.70 | | | | 0.98 | | | 1.00 | | | | – | | | | – | | | | | – | | | – | | | – | | | | | | | |
| 6 | 0.52 | | 0.24 | | | | 0.70 | | | 0.98 | | | | 1.00 | | | | – | | | | | – | | | – | | | – | | | | | | | |
| 7 | **0.0151** | | **0.0029** | | | | **0.0365** | | | 0.24 | | | | 0.51 | | | | 0.92 | | | | | – | | | – | | | – | | | | | | | |
| 8 | **0.0365** | | **0.0080** | | | | 0.08 | | | 0.39 | | | | 0.70 | | | | 0.98 | | | | | 1.00 | | | – | | | – | | | | | | | |
| 9 | **0.0080** | | **0.0014** | | | | **0.0205** | | | 0.16 | | | | 0.39 | | | | 0.85 | | | | | 1.00 | | | 1.00 | | | – | | | | | | | |
| 10 | **0.0014** | | **0.0002** | | | | **0.0041** | | | **0.0479** | | | | 0.16 | | | | 0.58 | | | | | 1.00 | | | 1.00 | | | 1.00 | | | | | | | |
| 1970s: _sm_ = 72.86, d. f. = 9, *p* < **0.0001**  *n*: 9 individuals, 101 subsections | | | | | | | | | | | | | | | | | | | | | | | | | | | | | | | | | | | | |
| Subsections | 1 (anterior) | | 2 | | | | 3 | | | 4 | | | | 5 | | | 6 | | | | | | 7 | | | 8 | | | 9 (posterior) | | | | | | | |
| 2 | 0.74 | | – | | | | – | | | – | | | | – | | | – | | | | | | – | | | – | | | – | | | | | | | |
| 3 | 0.98 | | 1.00 | | | | – | | | – | | | | – | | | – | | | | | | – | | | – | | | – | | | | | | | |
| 4 | 0.79 | | 1.00 | | | | 1.00 | | | – | | | | – | | | – | | | | | | – | | | – | | | – | | | | | | | |
| 5 | 0.37 | | 1.00 | | | | 0.98 | | | 1.00 | | | | – | | | – | | | | | | – | | | – | | | – | | | | | | | |
| 6 | **0.0459** | | 0.93 | | | | 0.58 | | | 0.90 | | | | 1.00 | | | – | | | | | | – | | | – | | | – | | | | | | | |
| 7 | **0.0006** | | 0.23 | | | | **0.0459** | | | 0.20 | | | | 0.58 | | | 0.98 | | | | | | – | | | – | | | – | | | | | | | |
| 8 | **<0.0001** | | **0.0407** | | | | **0.0046** | | | **0.0318** | | | | 0.18 | | | 0.72 | | | | | | 1.00 | | | – | | | – | | | | | | | |
| 9 | **<0.0001** | | **0.0166** | | | | **0.0015** | | | **0.0127** | | | | 0.09 | | | 0.53 | | | | | | 1.00 | | | 1.00 | | | – | | | | | | | |
| 10 | **<0.0001** | | **0.0318** | | | | **0.0034** | | | **0.0247** | | | | 0.15 | | | 0.66 | | | | | | 1.00 | | | 1.00 | | | 1.00 | | | | | | | |
| 2000s: _sm_ = 86.14, d. f. = 10, *p* < **0.0001**  *n*: 10 individuals, 123 subsections | | | | | | | | | | | | | | | | | | | | | | | | | | | | | | | | | | | | |
| Subsections | 1 (anterior) | | | 2 | | | 3 | | | 4 | | | | 5 | | | | 6 | | | 7 | | | | 8 | | | 9 | | | | 10 (posterior) | | | | |
| 2 | 0.84 | | | – | | | – | | | – | | | | – | | | | – | | | – | | | | – | | | – | | | | – | | | | |
| 3 | 0.54 | | | 1.00 | | | – | | | – | | | | – | | | | – | | | – | | | | – | | | – | | | | – | | | | |
| 4 | 0.63 | | | 1.00 | | | 1.00 | | | – | | | | – | | | | – | | | – | | | | – | | | – | | | | – | | | | |
| 5 | 0.49 | | | 1.00 | | | 1.00 | | | 1.00 | | | | – | | | | – | | | – | | | | – | | | – | | | | – | | | | |
| 6 | 0.09 | | | 0.97 | | | 1.00 | | | 1.00 | | | | 1.00 | | | | – | | | – | | | | – | | | – | | | | – | | | | |
| 7 | **0.0044** | | | 0.49 | | | 0.81 | | | 0.73 | | | | 0.84 | | | | 1.00 | | | – | | | | – | | | – | | | | – | | | | |
| 8 | **0.0001** | | | 0.08 | | | 0.25 | | | 0.19 | | | | 0.29 | | | | 0.81 | | | 1.00 | | | | – | | | – | | | | – | | | | |
| 9 | **<0.0001** | | | **0.0084** | | | **0.0427** | | | **0.0277** | | | | 0.0525 | | | | 0.35 | | | 0.92 | | | | 1.00 | | | – | | | | – | | | | |
| 10 | **<0.0001** | | | **0.0247** | | | 0.10 | | | 0.07 | | | | 0.12 | | | | 0.56 | | | 0.98 | | | | 1.00 | | | 1.00 | | | | – | | | | |
| 11 | **<0.0001** | | | **0.0138** | | | 0.06 | | | **0.0427** | | | | 0.08 | | | | 0.44 | | | 0.95 | | | | 1.00 | | | 1.00 | | | | 1.00 | | | | |
| 2010s: _sm_ = 119.25, d. f. = 14, *p* < **0.0001**  *n*: 13 individuals, 153 subsections | | | | | | | | | | | | | | | | | | | | | | | | | | | | | | | | | | | | |
| Subsections | 1 | 2 | | | | 3 | | | 4 | | | 5 | | | | 6 | | | 7 | | | 8 | | | 9 | | | 10 | | | 11 | | | 12 | 13 | 14 |
| 2 | 1.00 | – | | | | – | | | – | | | – | | | | – | | | – | | | – | | | – | | | – | | | – | | | – | – | – |
| 3 | 0.92 | 1.00 | | | | – | | | – | | | – | | | | – | | | – | | | – | | | – | | | – | | | – | | | – | – | – |
| 4 | 0.99 | 1.00 | | | | 1.00 | | | – | | | – | | | | – | | | – | | | – | | | – | | | – | | | – | | | – | – | – |
| 5 | 0.85 | 0.99 | | | | 1.00 | | | 1.00 | | | – | | | | – | | | – | | | – | | | – | | | – | | | – | | | – | – | – |
| 6 | 0.07 | 0.28 | | | | 0.97 | | | 0.85 | | | 0.99 | | | | – | | | – | | | – | | | – | | | – | | | – | | | – | – | – |
| 7 | 0.15 | 0.48 | | | | 1.00 | | | 0.96 | | | 1.00 | | | | 1.00 | | | – | | | – | | | – | | | – | | | – | | | – | – | – |
| 8 | **0.0176** | 0.10 | | | | 0.83 | | | 0.59 | | | 0.90 | | | | 1.00 | | | 1.00 | | | – | | | – | | | – | | | – | | | – | – | – |
| 9 | **0.0005** | **0.0056** | | | | 0.25 | | | 0.10 | | | 0.34 | | | | 1.00 | | | 0.97 | | | 1.00 | | | – | | | – | | | – | | | – | – | – |
| 10 | **0.0005** | **0.0051** | | | | 0.23 | | | 0.10 | | | 0.32 | | | | 1.00 | | | 0.96 | | | 1.00 | | | 1.00 | | | – | | | – | | | – | – | – |
| 11 | **0.0001** | **0.0016** | | | | 0.12 | | | **0.0414** | | | 0.17 | | | | 0.97 | | | 0.88 | | | 1.00 | | | 1.00 | | | 1.00 | | | – | | | – | – | – |
| 12 | **<0.0001** | **0.0002** | | | | **0.0250** | | | **0.0069** | | | **0.0414** | | | | 0.77 | | | 0.55 | | | 0.95 | | | 1.00 | | | 1.00 | | | 1.00 | | | – | – | – |
| 13 | **<0.0001** | **0.0002** | | | | **0.0273** | | | **0.0076** | | | **0.0449** | | | | 0.78 | | | 0.57 | | | 0.95 | | | 1.00 | | | 1.00 | | | 1.00 | | | 1.00 | – | – |
| 14 | **<0.0001** | **<0.0001** | | | | **0.0016** | | | **0.0003** | | | **0.0031** | | | | 0.28 | | | 0.13 | | | 0.55 | | | 0.98 | | | 0.98 | | | 1.00 | | | 1.00 | 1.00 | – |
| 15 | **<0.0001** | **<0.0001** | | | | **0.0008** | | | **0.0002** | | | **0.0016** | | | | 0.20 | | | 0.09 | | | 0.44 | | | 0.95 | | | 0.95 | | | 0.99 | | | 1.00 | 1.00 | 1.00 |
| Contemporary (2016, 2017, 2019)^1^: _sm_ = 89.09, d. f. = 10, *p* < **0.0001**  *n*: 10 individuals, 165 subsections | | | | | | | | | | | | | | | | | | | | | | | | | | | | | | | | | | | | |
| Subsections | 1 (anterior) | | | | 2 | | | 3 | | | 4 | | | | 5 | | | | | 6 | | | | 7 | | | 8 | | | 9 | | | 10 (posterior) | | | |
| 2 | 1.00 | | | | – | | | – | | | – | | | | – | | | | | – | | | | – | | | – | | | – | | | – | | | |
| 3 | 1.00 | | | | 1.00 | | | – | | | – | | | | – | | | | | – | | | | – | | | – | | | – | | | – | | | |
| 4 | 0.96 | | | | 1.00 | | | 1.00 | | | – | | | | – | | | | | – | | | | – | | | – | | | – | | | – | | | |
| 5 | 0.83 | | | | 0.96 | | | 1.00 | | | 1.00 | | | | – | | | | | – | | | | – | | | – | | | – | | | – | | | |
| 6 | 0.30 | | | | 0.54 | | | 0.84 | | | 0.99 | | | | 1.00 | | | | | – | | | | – | | | – | | | – | | | – | | | |
| 7 | **0.0090** | | | | **0.0310** | | | 0.12 | | | 0.39 | | | | 0.64 | | | | | 0.98 | | | | – | | | – | | | – | | | – | | | |
| 8 | **0.0020** | | | | **0.0080** | | | **0.0411** | | | 0.18 | | | | 0.37 | | | | | 0.88 | | | | 1.00 | | | – | | | – | | | – | | | |
| 9 | **0.0017** | | | | **0.0072** | | | **0.0375** | | | 0.17 | | | | 0.35 | | | | | 0.87 | | | | 1.00 | | | 1.00 | | | – | | | – | | | |
| 10 | **<0.0001** | | | | **<0.0001** | | | **0.0004** | | | **0.0041** | | | | **0.0153** | | | | | 0.17 | | | | 0.90 | | | 0.99 | | | 0.99 | | | – | | | |
| 11 | **0.0002** | | | | **0.0012** | | | **0.0080** | | | **0.0494** | | | | 0.13 | | | | | 0.60 | | | | 1.00 | | | 1.00 | | | 1.00 | | | 1.00 | | | |

^1^published in Golikov *et al.* 2022 and reused in this study.

Table S13. Ontogenetic changes in isotopic niche overlap in *Gonatus fabricii*, and their comparison among the time series. ML – mantle length

| Time series | | Period | | | |
| --- | --- | --- | --- | --- | --- |
|  |  | Initial | 1^st^ stable | Change | Main stable |
| 1900s | Subsections | 1 | 2 | 3–5 | 6+ (8 analysed) |
|  | ML, mm | 6.6–7.1 | 11.6–12.8 | 17.6–35.9 | ˃ 39.4 |
|  | Commentary | – | – | Overlap shrinkage | – |
| 1930s | Subsections | 1 | 2, 3 | 4–6 | 7+ (10 analysed) |
|  | ML, mm | 7.1–7.5 | 11.9–19.3 | 24.6–44.1 | ˃ 49.0 |
|  | Commentary | – | Relatively prolonged | Overlap shrinkage | Secondary increase at subsection 9 |
| 1970s | Subsections | 1 | 2 | 3–7 | 8+ (12 analysed) |
|  | ML, mm | 7.0–7.5 | 11.5–12.8 | 17.1–53.7 | ˃ 54.9 |
|  | Commentary | – | – | Overlap shrinkage | – |
| 2000s | Subsections | 1, 2 | 3 | 4–6 | 7+ (11 analysed) |
|  | ML, mm | 7.1–12.8 | 17.9–19.3 | 24.6–44.1 | ˃ 49.0 |
|  | Commentary | Relatively prolonged | – | No overlap shrinkage | – |
| 2010s | Subsections | 1 | 2–4 | 5, 6 | 7+ (15 analysed) |
|  | ML, mm | 6.6–7.1 | 11.7–27.5 | 32.0–44.9 | ˃ 48.4 |
|  | Commentary | – | Relatively prolonged | No overlap shrinkage; relatively shortened | Secondary increase at subsections 11 and 14 |
| Contemporary  (2016, 2017, 2019)^1^ | Subsections | 1, 2 | 3 | 4–6 | 7+ (14 analysed) |
|  | ML, mm | 7.0–12.8 | 17.3–19.3 | 23.7–44.2 | ˃ 47.0 |
|  | Commentary | Relatively prolonged | – | No overlap shrinkage | – |

^1^published in Golikov *et al.* 2022 and reused in this study.

Table S14. Overlap between isotopic niches for different upper beak subsections of *Gonatus fabricii*. *n* – sample size. Large overlap values (treated as significant overlap) are in **bold**

| Subsections | 1900s | | | | | | | | | | | | | | | |
| --- | --- | --- | --- | --- | --- | --- | --- | --- | --- | --- | --- | --- | --- | --- | --- | --- |
|  | *n* | 1 | | 2 | | 3 | | 4 | | 5 | | 6 | | 7 | | 8 |
| 1 | 8 | – | | **62.15** | | 52.13 | | 7.34 | | 0.79 | | 0.06 | | 0.02 | | 0.05 |
| 2 | 7 | **86.01** | | – | | **89.37** | | 26.63 | | 1.55 | | 0.02 | | 0.00 | | 0.00 |
| 3 | 8 | 47.18 | | 43.82 | | – | | 59.64 | | 14.19 | | 1.22 | | 0.29 | | 1.30 |
| 4 | 8 | 18.42 | | 15.17 | | **77.16** | | – | | 51.79 | | 11.50 | | 5.35 | | 19.52 |
| 5 | 8 | 3.05 | | 1.17 | | 30.38 | | **77.85** | | – | | **69.19** | | 47.44 | | **70.42** |
| 6 | 9 | 0.39 | | 0.03 | | 8.75 | | 51.38 | | **88.56** | | – | | **83.96** | | **92.23** |
| 7 | 9 | 0.16 | | 0.00 | | 4.35 | | 44.34 | | **79.44** | | **86.71** | | – | | **95.25** |
| 8 | 9 | 0.17 | | 0.00 | | 5.76 | | 51.56 | | **66.53** | | **66.38** | | **69.42** | | – |
| Subsections | 1930s | | | | | | | | | | | | | | | |
|  | *n* | 1 | | 2 | | 3 | | 4 | | 5 | | 6 | 7 | 8 | 9 | 10 |
| 1 | 6 | – | | **82.65** | | 56.79 | | 42.42 | | 25.60 | | 0.15 | 0.60 | 0.18 | 0.23 | 0.21 |
| 2 | 7 | 45.52 | | – | | **80.83** | | **62.73** | | 17.33 | | 0.02 | 0.76 | 0.10 | 0.08 | 0.19 |
| 3 | 7 | 13.47 | | **74.96** | | – | | **79.45** | | 9.58 | | 0.09 | 1.98 | 0.05 | 0.38 | 0.24 |
| 4 | 7 | 10.71 | | 36.45 | | **62.04** | | – | | 26.93 | | 2.79 | 10.17 | 0.84 | 2.02 | 0.90 |
| 5 | 7 | 9.57 | | 8.51 | | 12.28 | | 52.92 | | – | | 49.20 | 58.33 | 16.27 | 23.94 | 6.80 |
| 6 | 7 | 0.44 | | 0.06 | | 0.19 | | 6.88 | | 34.15 | | – | **90.98** | **72.03** | **76.39** | 54.29 |
| 7 | 7 | 0.29 | | 0.18 | | 0.42 | | 4.81 | | 16.28 | | **63.59** | – | **68.98** | **80.33** | **67.79** |
| 8 | 7 | 0.16 | | 0.03 | | 0.05 | | 2.15 | | 8.27 | | **80.56** | **95.26** | – | **93.31** | **81.40** |
| 9 | 7 | 0.15 | | 0.05 | | 0.13 | | 2.02 | | 6.20 | | **63.06** | **93.12** | **80.47** | – | **81.53** |
| 10 | 7 | 0.08 | | 0.05 | | 0.08 | | 1.20 | | 2.32 | | 53.51 | **95.48** | **78.03** | **94.15** | – |
| Subsections | 1970s | | | | | | | | | | | | | | | |
|  | *n* | 1 | | 2 | | 3 | | 4 | 5 | 6 | 7 | 8 | 9 | 10 | 11 | 12 |
| 1 | 9 | – | | **75.52** | | 55.45 | | 37.99 | 4.76 | 0.55 | 0.37 | 0.18 | 0.37 | 2.82 | 0.29 | 0.00 |
| 2 | 7 | **81.87** | | – | | **79.82** | | **79.24** | 17.10 | 1.17 | 1.13 | 0.66 | 1.23 | 5.96 | 0.97 | 0.03 |
| 3 | 9 | 40.55 | | **67.44** | | – | | **92.13** | 49.40 | 3.96 | 3.30 | 1.61 | 2.74 | 6.00 | 3.73 | 0.32 |
| 4 | 9 | 15.34 | | 33.28 | | **79.26** | | – | **70.04** | 14.21 | 10.54 | 7.51 | 8.11 | 12.58 | 10.69 | 1.90 |
| 5 | 9 | 1.87 | | 5.57 | | 36.18 | | **74.80** | – | **65.30** | 55.52 | 48.40 | 48.21 | 55.22 | 51.29 | 20.19 |
| 6 | 9 | 0.16 | | 0.42 | | 4.25 | | 27.06 | **81.57** | – | **92.66** | **90.86** | **90.41** | **91.67** | **84.56** | 58.35 |
| 7 | 9 | 0.10 | | 0.28 | | 1.99 | | 14.32 | **61.61** | **85.40** | – | **92.68** | **92.83** | **92.40** | **81.68** | **66.32** |
| 8 | 9 | 0.08 | | 0.18 | | 1.36 | | 10.87 | 51.59 | **79.65** | **90.89** | – | **91.73** | **89.49** | **74.64** | **62.52** |
| 9 | 9 | 0.12 | | 0.28 | | 1.64 | | 11.10 | 53.71 | **81.08** | **91.49** | **92.28** | – | **92.16** | **79.34** | **65.78** |
| 10 | 9 | 0.48 | | 0.96 | | 2.86 | | 13.73 | 56.14 | **78.89** | **87.84** | **87.61** | **89.78** | – | **80.09** | **62.49** |
| 11 | 7 | 0.09 | | 0.21 | | 2.31 | | 13.93 | 43.17 | **83.86** | **91.63** | **90.98** | **92.29** | **94.13** | – | **77.08** |
| 12 | 6 | 0.01 | | 0.01 | | 0.36 | | 4.77 | 40.24 | **69.06** | **88.87** | **90.24** | **90.85** | **89.39** | **84.46** | – |
| Subsections | 2000s | | | | | | | | | | | | | | | |
|  | *n* | 1 | | 2 | | 3 | | 4 | 5 | 6 | | 7 | 8 | 9 | 10 | 11 |
| 1 | 10 | – | | **85.39** | | **63.79** | | **63.87** | 33.02 | 24.86 | | 11.81 | 10.33 | 19.96 | 20.68 | 21.83 |
| 2 | 8 | **88.23** | | – | | **80.89** | | **79.27** | 46.34 | 31.50 | | 13.74 | 12.16 | 24.08 | 24.37 | 25.86 |
| 3 | 8 | **84.87** | | **88.56** | | – | | **93.31** | **75.28** | 54.51 | | 29.44 | 26.12 | 39.61 | 38.16 | 41.87 |
| 4 | 9 | **67.72** | | **67.22** | | **80.56** | | – | **86.06** | **70.05** | | 47.93 | 43.77 | 52.03 | 49.51 | 53.35 |
| 5 | 10 | 43.06 | | 36.57 | | 57.84 | | **84.06** | – | **89.39** | | **79.11** | **76.71** | **79.45** | **77.53** | **79.76** |
| 6 | 10 | 25.73 | | 20.19 | | 36.06 | | **65.87** | **89.58** | – | | **89.84** | **89.04** | **90.63** | **89.84** | **90.48** |
| 7 | 10 | 17.30 | | 12.70 | | 26.41 | | 57.40 | **88.46** | **94.98** | | – | **93.07** | **93.73** | **93.19** | **93.38** |
| 8 | 10 | 14.28 | | 10.81 | | 22.30 | | 49.87 | **86.06** | **93.65** | | **92.15** | – | **94.47** | **93.71** | **93.95** |
| 9 | 10 | 15.27 | | 13.18 | | 22.80 | | 44.46 | **79.91** | **89.82** | | **87.73** | **89.56** | – | **92.69** | **93.01** |
| 10 | 10 | 16.12 | | 13.64 | | 23.55 | | 45.48 | **80.61** | **90.47** | | **88.63** | **89.85** | **93.64** | – | **93.00** |
| 11 | 10 | 16.81 | | 14.16 | | 24.63 | | 46.39 | **81.12** | **90.43** | | **88.06** | **89.52** | **93.54** | **92.66** | – |
| Subsections | 2010s | | | | | | | | | | | | | | | |
|  | *n* | 1 | 2 | 3 | 4 | 5 | 6 | 7 | 8 | 9 | 10 | 11 | 12 | 13 | 14 | 15 |
| 1 | 10 | – | **85.64** | **87.37** | 57.68 | 59.94 | 25.84 | 0.87 | 0.23 | 0.24 | 0.30 | 0.08 | 0.54 | 1.13 | 0.23 | 0.00 |
| 2 | 10 | **77.42** | – | **95.38** | **85.12** | **82.27** | 58.01 | 8.28 | 2.49 | 3.06 | 3.29 | 0.63 | 4.37 | 5.77 | 1.10 | 0.08 |
| 3 | 9 | 47.96 | **77.82** | – | **87.19** | **86.35** | **71.24** | 28.90 | 14.93 | 17.81 | 17.86 | 5.12 | 18.46 | 20.80 | 8.34 | 2.11 |
| 4 | 10 | 27.66 | **65.50** | **90.34** | – | **87.96** | **76.01** | 39.47 | 17.83 | 24.02 | 22.94 | 4.42 | 25.48 | 26.82 | 12.11 | 3.44 |
| 5 | 10 | 15.53 | 38.98 | **79.17** | **76.06** | – | **85.44** | **65.55** | 50.27 | 55.94 | 53.22 | 32.05 | 57.24 | **61.24** | 41.29 | 21.07 |
| 6 | 9 | 6.53 | 22.12 | 57.24 | 49.58 | **81.20** | – | **76.15** | **65.89** | **69.58** | **67.99** | 51.18 | **73.86** | **75.31** | **61.24** | 37.34 |
| 7 | 10 | 0.68 | 7.38 | 52.20 | 40.75 | **89.29** | **96.96** | – | **89.01** | **91.85** | **89.73** | **76.43** | **93.03** | **93.25** | **78.80** | 42.41 |
| 8 | 10 | 0.21 | 3.44 | 41.42 | 27.09 | **86.29** | **96.70** | **94.66** | – | **95.02** | **93.26** | **88.51** | **96.17** | **96.05** | **83.83** | 48.30 |
| 9 | 10 | 0.25 | 3.63 | 41.20 | 28.41 | **82.83** | **94.72** | **92.49** | **90.35** | – | **91.39** | **85.25** | **94.44** | **93.83** | **81.12** | 46.36 |
| 10 | 10 | 0.34 | 4.32 | 42.70 | 28.98 | **81.78** | **95.61** | **93.39** | **91.89** | 94.57 | – | **85.38** | **94.85** | **93.95** | **78.24** | 39.90 |
| 11 | 9 | 0.12 | 1.11 | 23.73 | 11.49 | **70.51** | **80.43** | **77.55** | **78.89** | **82.63** | **78.04** | – | **84.71** | **84.51** | **72.33** | 50.72 |
| 12 | 10 | 0.24 | 2.72 | 31.64 | 21.62 | **76.79** | **93.45** | **87.57** | **84.84** | **87.83** | **83.58** | **81.54** | – | **92.95** | **86.38** | **62.84** |
| 13 | 10 | 0.34 | 2.75 | 29.85 | 20.47 | **78.73** | **92.50** | **84.21** | **81.38** | **83.45** | **77.36** | **80.35** | **91.36** | – | **87.75** | **68.29** |
| 14 | 10 | 0.05 | 0.73 | 17.32 | 11.60 | **70.22** | **88.93** | **73.13** | **69.51** | **71.70** | **61.66** | **73.85** | **88.18** | **91.74** | – | **86.49** |
| 15 | 10 | 0.00 | 0.11 | 8.92 | 6.60 | **62.99** | **71.96** | 50.06 | 46.05 | 48.74 | 35.43 | **65.05** | **74.58** | **83.03** | **88.44** | – |
| Subsections | Contemporary (2016, 2017, 2019)^1^ | | | | | | | | | | | | | | | |
|  | *n* | 1 | | 2 | 3 | 4 | 5 | 6 | 7 | 8 | 9 | 10 | 11 | 12 | 13 | 14 |
| 1 | 11 | – | | **92.71** | **92.42** | **78.44** | 38.19 | 54.16 | 11.87 | 3.56 | 4.61 | 2.04 | 2.79 | 9.81 | 22.07 | 22.27 |
| 2 | 9 | **90.60** | | – | **93.89** | **85.06** | 47.09 | 44.30 | 20.03 | 8.80 | 10.68 | 4.71 | 6.34 | 17.06 | 31.85 | 31.88 |
| 3 | 11 | **79.75** | | **86.85** | – | **90.57** | **66.91** | **78.62** | 41.08 | 26.00 | 29.45 | 16.83 | 20.59 | 37.75 | 54.10 | 52.05 |
| 4 | 13 | **62.54** | | **74.95** | **90.07** | – | **78.29** | **87.83** | 58.53 | 44.79 | 48.77 | 31.09 | 36.13 | 56.25 | **71.87** | **67.94** |
| 5 | 12 | 47.67 | | **67.48** | **92.39** | **95.93** | – | **95.39** | **80.35** | **68.76** | **73.69** | 50.61 | 58.54 | **80.58** | **90.14** | **83.66** |
| 6 | 11 | 44.39 | | **62.63** | **88.51** | **94.46** | **87.56** | – | **76.99** | **64.98** | **69.68** | 50.79 | 57.34 | **76.51** | **87.68** | **82.58** |
| 7 | 13 | 25.04 | | 47.13 | **86.90** | **96.58** | **90.32** | **97.65** | – | **86.56** | **90.84** | **81.90** | **86.18** | **94.63** | **98.38** | **96.51** |
| 8 | 11 | 20.08 | | 44.92 | **85.95** | **96.95** | **89.57** | **96.31** | **92.62** | – | **94.82** | **81.24** | **85.48** | **94.47** | **97.99** | **95.39** |
| 9 | 12 | 19.50 | | 42.90 | **84.96** | **96.35** | **89.18** | **96.30** | **93.22** | **92.36** | – | **82.74** | **86.87** | **95.06** | **98.05** | **95.49** |
| 10 | 10 | 14.73 | | 36.25 | **85.86** | **97.47** | **91.88** | **98.29** | **96.60** | **91.92** | **95.15** | – | **95.03** | **97.83** | **99.22** | **97.62** |
| 11 | 11 | 15.42 | | 37.30 | **85.55** | **97.23** | **91.00** | **98.13** | **96.51** | **91.41** | **94.77** | **92.05** | – | **97.65** | **99.29** | **97.63** |
| 12 | 9 | 19.23 | | 40.07 | **82.38** | **94.09** | **86.90** | **95.60** | **90.93** | **83.63** | **88.19** | **79.84** | **83.93** | – | **97.10** | **93.55** |
| 13 | 10 | 19.31 | | 35.63 | **72.26** | **87.70** | **75.32** | **89.50** | **82.27** | **72.47** | **77.81** | **66.90** | **71.97** | **84.92** | – | **90.05** |
| 14 | 8 | 19.00 | | 34.50 | **68.76** | **85.96** | **70.46** | **87.37** | **81.81** | **71.98** | **77.00** | **65.55** | **70.75** | **83.37** | **93.68** | – |

^1^published in Golikov *et al.* 2022 and reused in this study.

Table S15. Ontogenetic changes in *δ*^13^C values and Trophic Position (TP) of *Todarodes sagittatus*. GAMM – generalized additive mixed effect model, n/a – not applicable. Significant *p*-values are in **bold**

| *δ*^13^C | | | | | | | | | | | |
| --- | --- | --- | --- | --- | --- | --- | --- | --- | --- | --- | --- |
| Time series | *n* | | Main increase | | After main increase | | *s*, min–max  (mean ± SE) | GAMM | | | |
|  | individuals | subsections | rate | subsections | rate | subsections |  | Intercept  (mean ± SE) | *k*, *s*(Subsection)^1^ | *p* | Deviance  explained, % |
| 1840s | 4 | 51 | 0.37 ‰ | 1 to 5 | 0.02‰ | 5 to 12 | 0.23–0.66 (0.43 ± 0.10) | -17.05 ± 0.28 | 3, 1.00 | **0.0001** | 62.5 |
| 1880s | 10 | 167 | 0.35 ‰ | 1 to 5 | -0.01‰ | 5 to 14 | 0.63–0.89 (0.82 ± 0.03) | -16.44 ± 0.07 | 3, 1.99 | **<0.0001** | 91.8 |
| 1890s | 10 | 127 | 0.20 ‰ | 1 to 3 | -0.01‰ | 3 to 18 | 0.23–0.58 (0.41 ± 0.03) | -17.48 ± 0.15 | 4, 2.79 | **<0.0001** | 76.6 |
| Contemporary  (2016–2018, 2023) | 7 | 124 | 0.29 ‰ | 1 to 5 | 0.07‰ | 5 to 21 | 0.31–0.76 (0.49 ± 0.06) | -15.80 ± 0.18 | 3, 1.97 | **<0.0001** | 89.8 |
| Trophic position | | | | | | | | | | | |
| 1840s | 4 | 51 | 0.34 TP | 2 to 4 | 0.08 TP | 4 to 12 | 0.85–0.93 (0.88 ± 0.02) | 2.90 ± 0.10 | 3, 1.89 | **<0.0001** | 88.7 |
| 1880s | 10 | 167 | 0.42 TP | 1 to 5 | 0.10 TP | 5 to 14 | 0.96–0.98 (0.97 ± 0.01) | 3.82 ± 0.05 | 4, 2.85 | **<0.0001** | 92.0 |
| 1890s | 10 | 127 | 0.16 TP | 2 to 7 | 0.05 TP | 7 to 18 | 0.79–0.96 (0.90 ± 0.02) | 3.81 ± 0.05 | 3, 1.95 | **<0.0001** | 82.1 |
| Contemporary  (2016–2018, 2023) | 7 | 124 | 0.28 TP | 1 to 3 | 0.05 TP | 3 to 21 | 0.46–0.81 (0.58 ± 0.05) | 4.08 ± 0.15 | 3, 1.96 | **<0.0001** | 93.5 |

^1^in GAMMs, *k* indicates the number of knots and *s* represents the smoother (subsection), where values are effective degrees of freedom.

Table S16. Comparison of *δ*^13^C values among different upper beak subsections in *Todarodes sagittatus*, using the Skillings–Mack test with the Nemenyi post hoc test. *n* – sample size. Significant *p*-values are in **bold**

| Subsections | 1840s: _sm_ = 38.62, d. f. = 11, *p* < **0.0001**  *n*: 4 individuals, 51 subsections | | | | | | | | | | | | | | | | | | | | | | | | | | | | |  |  |
| --- | --- | --- | --- | --- | --- | --- | --- | --- | --- | --- | --- | --- | --- | --- | --- | --- | --- | --- | --- | --- | --- | --- | --- | --- | --- | --- | --- | --- | --- | --- | --- |
|  | 1 (anterior) | | 2 | | | 3 | | | | 4 | | | 5 | | 6 | | 7 | | | | 8 | | 9 | | | 10 | | 11 (posterior) | |  |  |
| 2 | 1.00 | | – | | | – | | | | – | | | – | | – | | – | | | | – | | – | | | – | | – | |  |  |
| 3 | 1.00 | | 1.00 | | | – | | | | – | | | – | | – | | – | | | | – | | – | | | – | | – | |  |  |
| 4 | 0.99 | | 1.00 | | | 1.00 | | | | – | | | – | | – | | – | | | | – | | – | | | – | | – | |  |  |
| 5 | 0.72 | | 0.84 | | | 1.00 | | | | 1.00 | | | – | | – | | – | | | | – | | – | | | – | | – | |  |  |
| 6 | 0.95 | | 0.98 | | | 1.00 | | | | 1.00 | | | 1.00 | | – | | – | | | | – | | – | | | – | | – | |  |  |
| 7 | 0.07 | | 0.13 | | | 0.65 | | | | 0.69 | | | 0.99 | | 0.88 | | – | | | | – | | – | | | – | | – | |  |  |
| 8 | **0.0060** | | **0.0130** | | | 0.18 | | | | 0.20 | | | 0.75 | | 0.40 | | 1.00 | | | | – | | – | | | – | | – | |  |  |
| 9 | **0.0050** | | **0.0110** | | | 0.16 | | | | 0.18 | | | 0.72 | | 0.37 | | 1.00 | | | | 1.00 | | – | | | – | | – | |  |  |
| 10 | 0.06 | | 0.11 | | | 0.62 | | | | 0.65 | | | 0.99 | | 0.86 | | 1.00 | | | | 1.00 | | 1.00 | | | – | | – | |  |  |
| 11 | 0.37 | | 0.51 | | | 0.97 | | | | 0.98 | | | 1.00 | | 1.00 | | 1.00 | | | | 0.96 | | 0.95 | | | 1.00 | | – | |  |  |
| 12 | 0.47 | | 0.62 | | | 0.99 | | | | 0.99 | | | 1.00 | | 1.00 | | 1.00 | | | | 0.92 | | 0.90 | | | 1.00 | | 1.00 | |  |  |
| 1880s: _sm_ = 91.45, d. f. = 11, *p* < **0.0001**  *n*: 10 individuals, 167 subsections | | | | | | | | | | | | | | | | | | | | | | | | | | | | | |  |  |
| Subsections | 1 (anterior) | | 2 | | | 3 | | | | 4 | | | 5 | | 6 | | 7 | | | | 8 | | 9 | | | 10 | | 11 (posterior) | |  |  |
| 2 | 1.00 | | – | | | – | | | | – | | | – | | – | | – | | | | – | | – | | | – | | – | |  |  |
| 3 | 0.97 | | 1.00 | | | – | | | | – | | | – | | – | | – | | | | – | | – | | | – | | – | |  |  |
| 4 | 0.66 | | 0.96 | | | 1.00 | | | | – | | | – | | – | | – | | | | – | | – | | | – | | – | |  |  |
| 5 | 0.14 | | 0.48 | | | 0.93 | | | | 1.00 | | | – | | – | | – | | | | – | | – | | | – | | – | |  |  |
| 6 | **0.0060** | | 0.0519 | | | 0.33 | | | | 0.80 | | | 1.00 | | – | | – | | | | – | | – | | | – | | – | |  |  |
| 7 | **0.0006** | | **0.0076** | | | 0.09 | | | | 0.41 | | | 0.94 | | 1.00 | | – | | | | – | | – | | | – | | – | |  |  |
| 8 | **0.0003** | | **0.0041** | | | 0.06 | | | | 0.31 | | | 0.88 | | 1.00 | | 1.00 | | | | – | | – | | | – | | – | |  |  |
| 9 | **<0.0001** | | **<0.0001** | | | **0.0003** | | | | **0.0053** | | | 0.10 | | 0.64 | | 0.54 | | | | 0.97 | | – | | | – | | – | |  |  |
| 10 | **<0.0001** | | **<0.0001** | | | **0.0002** | | | | **0.0037** | | | 0.08 | | 0.57 | | 0.91 | | | | 0.95 | | 1.00 | | | – | | – | |  |  |
| 11 | **<0.0001** | | **<0.0001** | | | **0.0019** | | | | **0.0258** | | | 0.28 | | 0.89 | | 1.00 | | | | 1.00 | | 1.00 | | | 1.00 | | – | |  |  |
| 12 | **0.0189** | | 0.13 | | | 0.55 | | | | 0.94 | | | 1.00 | | 1.00 | | 1.00 | | | | 1.00 | | 0.41 | | | 0.35 | | 0.73 | |  |  |
| 1890s: _sm_ = 58.67, d. f. = 14, *p* < **0.0001**  *n*: 10 individuals, 127 subsections | | | | | | | | | | | | | | | | | | | | | | | | | | | | | | |  |
| Subsections | 1 | 2 | | 3 | | | 4 | | 5 | | 6 | | | 7 | | 8 | | 9 | | 10 | | 11 | | 12 | | | 13 | | 14 | |  |
| 2 | 1.00 | – | | – | | | – | | – | | – | | | – | | – | | – | | – | | – | | – | | | – | | – | |  |
| 3 | 1.00 | 0.81 | | – | | | – | | – | | – | | | – | | – | | – | | – | | – | | – | | | – | | – | |  |
| 4 | 1.00 | 0.55 | | 1.00 | | | – | | – | | – | | | – | | – | | – | | – | | – | | – | | | – | | – | |  |
| 5 | 0.99 | 0.44 | | 1.00 | | | 1.00 | | – | | – | | | – | | – | | – | | – | | – | | – | | | – | | – | |  |
| 6 | 1.00 | 0.63 | | 1.00 | | | 1.00 | | 1.00 | | – | | | – | | – | | – | | – | | – | | – | | | – | | – | |  |
| 7 | 0.87 | 0.14 | | 1.00 | | | 1.00 | | 1.00 | | 1.00 | | | – | | – | | – | | – | | – | | – | | | – | | – | |  |
| 8 | 0.80 | 0.10 | | 1.00 | | | 1.00 | | 1.00 | | 1.00 | | | 1.00 | | – | | – | | – | | – | | – | | | – | | – | |  |
| 9 | **0.0297** | **0.0003** | | 0.29 | | | 0.55 | | 0.67 | | 0.48 | | | 0.94 | | 0.97 | | – | | – | | – | | – | | | – | | – | |  |
| 10 | 0.10 | **0.0015** | | 0.55 | | | 0.81 | | 0.89 | | 0.75 | | | 1.00 | | 1.00 | | 1.00 | | – | | – | | – | | | – | | – | |  |
| 11 | 0.31 | **0.0101** | | 0.87 | | | 0.98 | | 0.99 | | 0.96 | | | 1.00 | | 1.00 | | 1.00 | | 1.00 | | – | | – | | | – | | – | |  |
| 12 | 0.55 | **0.0351** | | 0.97 | | | 1.00 | | 1.00 | | 1.00 | | | 1.00 | | 1.00 | | 1.00 | | 1.00 | | 1.00 | | – | | | – | | – | |  |
| 13 | 1.00 | 0.80 | | 1.00 | | | 1.00 | | 1.00 | | 1.00 | | | 1.00 | | 1.00 | | 0.31 | | 0.57 | | 0.88 | | 0.98 | | | – | | – | |  |
| 14 | 1.00 | 0.96 | | 1.00 | | | 1.00 | | 1.00 | | 1.00 | | | 0.99 | | 0.97 | | 0.12 | | 0.29 | | 0.63 | | 0.85 | | | 1.00 | | – | |  |
| 15 | 1.00 | 1.00 | | 1.00 | | | 1.00 | | 0.99 | | 1.00 | | | 0.85 | | 0.78 | | **0.0273** | | 0.09 | | 0.29 | | 0.53 | | | 1.00 | | 1.00 | |  |
| Contemporary (2016–2018, 2023): _sm_ = 49.56, d. f. = 8, *p* < **0.0001**  *n*: 7 individuals, 124 subsections | | | | | | | | | | | | | | | | | | | | | | | | | | | | | | | |
| Subsections | 1 (anterior) | | 2 | | 3 | | | 4 | | | | 5 | | | 6 | | | | 7 | | | | | | 8 (posterior) | | | | | | |
| 2 | 1.00 | | – | | – | | | – | | | | – | | | – | | | | – | | | | | | – | | | | | | |
| 3 | 0.91 | | 1.00 | | – | | | – | | | | – | | | – | | | | – | | | | | | – | | | | | | |
| 4 | 0.58 | | 0.87 | | 1.00 | | | – | | | | – | | | – | | | | – | | | | | | – | | | | | | |
| 5 | 0.08 | | 0.26 | | 0.83 | | | 0.98 | | | | – | | | – | | | | – | | | | | | – | | | | | | |
| 6 | **0.0065** | | **0.0348** | | 0.32 | | | 0.71 | | | | 1.00 | | | – | | | | – | | | | | | – | | | | | | |
| 7 | **0.0014** | | **0.0093** | | 0.14 | | | 0.44 | | | | 0.96 | | | 1.00 | | | | – | | | | | | – | | | | | | |
| 8 | **0.0004** | | **0.0031** | | 0.06 | | | 0.26 | | | | 0.87 | | | 1.00 | | | | 1.00 | | | | | | – | | | | | | |
| 9 | **<0.0001** | | **0.0006** | | **0.0184** | | | 0.11 | | | | 0.65 | | | 0.98 | | | | 1.00 | | | | | | 1.00 | | | | | | |

Table S17. Comparison of trophic position among different upper beak subsections in *Todarodes sagittatus*, using the Skillings–Mack test with the Nemenyi post hoc test. *n* – sample size. Significant *p*-values are in **bold**

| Subsections | 1840s: _sm_ = 104.85, d. f. = 11, *p* < **0.0001**  *n*: 4 individuals, 51 subsections | | | | | | | | | | | | | | | | | | | | | | | | | | |  |  |  |
| --- | --- | --- | --- | --- | --- | --- | --- | --- | --- | --- | --- | --- | --- | --- | --- | --- | --- | --- | --- | --- | --- | --- | --- | --- | --- | --- | --- | --- | --- | --- |
|  | 1 (anterior) | | 2 | | | 3 | | | 4 | | | 5 | | 6 | | | 7 | | | 8 | 9 | | 10 | | 11 (posterior) | | |  |  |  |
| 2 | 1.00 | | – | | | – | | | – | | | – | | – | | | – | | | – | – | | – | | – | | |  |  |  |
| 3 | 1.00 | | 1.00 | | | – | | | – | | | – | | – | | | – | | | – | – | | – | | – | | |  |  |  |
| 4 | 0.99 | | 0.98 | | | 1.00 | | | – | | | – | | – | | | – | | | – | – | | – | | – | | |  |  |  |
| 5 | 0.99 | | 0.98 | | | 1.00 | | | 1.00 | | | – | | – | | | – | | | – | – | | – | | – | | |  |  |  |
| 6 | 0.88 | | 0.84 | | | 0.99 | | | 1.00 | | | 1.00 | | – | | | – | | | – | – | | – | | – | | |  |  |  |
| 7 | 0.69 | | 0.62 | | | 0.95 | | | 1.00 | | | 1.00 | | 1.00 | | | – | | | – | – | | – | | – | | |  |  |  |
| 8 | 0.28 | | 0.23 | | | 0.65 | | | 0.95 | | | 0.97 | | 1.00 | | | 1.00 | | | – | – | | – | | – | | |  |  |  |
| 9 | 0.06 | | **0.0407** | | | 0.23 | | | 0.65 | | | 0.69 | | 0.92 | | | 0.99 | | | 1.00 | – | | – | | – | | |  |  |  |
| 10 | 0.11 | | 0.09 | | | 0.37 | | | 0.81 | | | 0.84 | | 0.98 | | | 1.00 | | | 1.00 | 1.00 | | – | | – | | |  |  |  |
| 11 | **0.0106** | | **0.0073** | | | 0.06 | | | 0.31 | | | 0.34 | | 0.65 | | | 0.86 | | | 0.99 | 1.00 | | 1.00 | | – | | |  |  |  |
| 12 | **0.0060** | | **0.0041** | | | **0.0407** | | | 0.23 | | | 0.25 | | 0.55 | | | 0.78 | | | 0.98 | 1.00 | | 1.00 | | 1.00 | | |  |  |  |
| 1880s: _sm_ = 104.85, d. f. = 11, *p* < **0.0001**  *n*: 10 individuals, 167 subsections | | | | | | | | | | | | | | | | | | | | | | | | | | | |  |  |  |
| Subsections | 1 (anterior) | | 2 | | | 3 | | | 4 | | | 5 | | 6 | | | 7 | | | 8 | 9 | | 10 | | 11 (posterior) | | |  |  |  |
| 2 | 1.00 | | – | | | – | | | – | | | – | | – | | | – | | | – | – | | – | | – | | |  |  |  |
| 3 | 0.91 | | 1.00 | | | – | | | – | | | – | | – | | | – | | | – | – | | – | | – | | |  |  |  |
| 4 | 0.82 | | 0.99 | | | 1.00 | | | – | | | – | | – | | | – | | | – | – | | – | | – | | |  |  |  |
| 5 | 0.35 | | 0.78 | | | 1.00 | | | 1.00 | | | – | | – | | | – | | | – | – | | – | | – | | |  |  |  |
| 6 | 0.08 | | 0.35 | | | 0.94 | | | 0.98 | | | 1.00 | | – | | | – | | | – | – | | – | | – | | |  |  |  |
| 7 | **0.0135** | | 0.10 | | | 0.66 | | | 0.78 | | | 0.99 | | 1.00 | | | – | | | – | – | | – | | – | | |  |  |  |
| 8 | **0.0011** | | **0.0135** | | | 0.24 | | | 0.35 | | | 0.82 | | 0.99 | | | 1.00 | | | – | – | | – | | – | | |  |  |  |
| 9 | **<0.0001** | | **0.0013** | | | 0.0519 | | | 0.09 | | | 0.41 | | 0.84 | | | 0.99 | | | 1.00 | – | | – | | – | | |  |  |  |
| 10 | **<0.0001** | | **<0.0001** | | | **0.0028** | | | **0.0060** | | | 0.06 | | 0.30 | | | 0.68 | | | 0.97 | 1.00 | | – | | – | | |  |  |  |
| 11 | **<0.0001** | | **<0.0001** | | | **0.0002** | | | **0.0005** | | | **0.0082** | | 0.07 | | | 0.28 | | | 0.70 | 0.97 | | 1.00 | | – | | |  |  |  |
| 12 | **<0.0001** | | **<0.0001** | | | **<0.0001** | | | **0.0001** | | | **0.0025** | | **0.0258** | | | 0.14 | | | 0.48 | 0.87 | | 1.00 | | 1.00 | | |  |  |  |
| 1890s: _sm_ = 129.93, d. f. = 14, *p* < **0.0001**  *n*: 10 individuals, 127 subsections | | | | | | | | | | | | | | | | | | | | | | | | | | | | |  |  |
| Subsections | 1 | 2 | | 3 | | | 4 | | | 5 | | | 6 | | | 7 | | 8 | | 9 | 10 | | 11 | 12 | | 13 | 14 | | | |
| 2 | 1.00 | – | | – | | | – | | | – | | | – | | | – | | – | | – | – | | – | – | | – | – | | | |
| 3 | 1.00 | 1.00 | | – | | | – | | | – | | | – | | | – | | – | | – | – | | – | – | | – | – | | | |
| 4 | 1.00 | 1.00 | | 1.00 | | | – | | | – | | | – | | | – | | – | | – | – | | – | – | | – | – | | | |
| 5 | 1.00 | 1.00 | | 1.00 | | | 1.00 | | | – | | | – | | | – | | – | | – | – | | – | – | | – | – | | | |
| 6 | 1.00 | 0.80 | | 0.97 | | | 1.00 | | | 1.00 | | | – | | | – | | – | | – | – | | – | – | | – | – | | | |
| 7 | 0.85 | 0.22 | | 0.52 | | | 0.93 | | | 0.97 | | | 1.00 | | | – | | – | | – | – | | – | – | | – | – | | | |
| 8 | 0.53 | 0.06 | | 0.21 | | | 0.68 | | | 0.80 | | | 0.99 | | | 1.00 | | – | | – | – | | – | – | | – | – | | | |
| 9 | 0.59 | 0.08 | | 0.25 | | | 0.74 | | | 0.84 | | | 1.00 | | | 1.00 | | 1.00 | | – | – | | – | – | | – | – | | | |
| 10 | 0.28 | **0.0176** | | 0.08 | | | 0.40 | | | 0.53 | | | 0.93 | | | 1.00 | | 1.00 | | 1.00 | – | | – | – | | – | – | | | |
| 11 | 0.08 | **0.0025** | | **0.0147** | | | 0.14 | | | 0.21 | | | 0.67 | | | 0.99 | | 1.00 | | 1.00 | 1.00 | | – | – | | – | – | | | |
| 12 | **0.0147** | **0.0003** | | **0.0020** | | | **0.0297** | | | 0.0526 | | | 0.31 | | | 0.88 | | 0.99 | | 0.98 | 1.00 | | 1.00 | – | | – | – | | | |
| 13 | **0.0004** | **<0.0001** | | **<0.0001** | | | **0.0010** | | | **0.0022** | | | **0.0297** | | | 0.31 | | 0.65 | | 0.59 | 0.88 | | 0.99 | 1.00 | | – | – | | | |
| 14 | **<0.0001** | **<0.0001** | | **<0.0001** | | | **0.0001** | | | **0.0003** | | | **0.0051** | | | 0.10 | | 0.31 | | 0.26 | 0.57 | | 0.89 | 0.99 | | 1.00 | – | | | |
| 15 | **<0.0001** | **<0.0001** | | **<0.0001** | | | **<0.0001** | | | **<0.0001** | | | **0.0018** | | | **0.0449** | | 0.17 | | 0.14 | 0.39 | | 0.75 | 0.96 | | 1.00 | 1.00 | | | |
| Contemporary (2016–2018, 2023): _sm_ = 49.56, d. f. = 8, *p* < **0.0001**  *n*: 7 individuals, 124 subsections | | | | | | | | | | | | | | | | | | | | | | | | | | | | | |  |
| Subsections | 1 (anterior) | | 2 | | 3 | | | 4 | | | 5 | | | | 6 | | | | 7 | | | 8 (posterior) | | | | | | | |  |
| 2 | 1.00 | | – | | – | | | – | | | – | | | | – | | | | – | | | – | | | | | | | |  |
| 3 | 0.94 | | 1.00 | | – | | | – | | | – | | | | – | | | | – | | | – | | | | | | | |  |
| 4 | 0.51 | | 0.83 | | 1.00 | | | – | | | – | | | | – | | | | – | | | – | | | | | | | |  |
| 5 | 0.14 | | 0.38 | | 0.87 | | | 1.00 | | | – | | | | – | | | | – | | | – | | | | | | | |  |
| 6 | **0.0348** | | 0.14 | | 0.58 | | | 0.96 | | | 1.00 | | | | – | | | | – | | | – | | | | | | | |  |
| 7 | **0.0014** | | **0.0093** | | 0.11 | | | 0.51 | | | 0.91 | | | | 0.99 | | | | – | | | – | | | | | | | |  |
| 8 | **0.0002** | | **0.0014** | | **0.0255** | | | 0.21 | | | 0.65 | | | | 0.91 | | | | 1.00 | | | – | | | | | | | |  |
| 9 | **<0.0001** | | **<0.0001** | | **0.00021** | | | **0.0348** | | | 0.21 | | | | 0.51 | | | | 0.96 | | | 1.00 | | | | | | | |  |

Table S18. Overlap between isotopic niches for different upper beak subsections of *Todarodes sagittatus*. *n* – sample size. Large overlap values (treated as significant overlap) are in **bold**

| Subsections | | 1840s | | | | | | | | | | | | | | | | | | | | | | | | | | | | | | | | | | | | | | | | |  |
| --- | --- | --- | --- | --- | --- | --- | --- | --- | --- | --- | --- | --- | --- | --- | --- | --- | --- | --- | --- | --- | --- | --- | --- | --- | --- | --- | --- | --- | --- | --- | --- | --- | --- | --- | --- | --- | --- | --- | --- | --- | --- | --- | --- |
|  |  | *n* | | | | 1 | | | | | | | | | | 2 | | 3 | | 4 | | 5 | | 6 | | 7 | | | | 8 | | | 9 | | | | 10 | | 11 | | 12 | |  |
| 1 | | 4 | | | | – | | | | | | | | | | 32.06 | | 26.73 | | 29.22 | | 15.99 | | 3.65 | | 3.63 | | | | 0.68 | | | 0.01 | | | | 0.04 | | 0.06 | | 0.11 | |  |
| 2 | | 4 | | | | 52.53 | | | | | | | | | | – | | 36.14 | | 34.82 | | 24.93 | | 3.67 | | 10.62 | | | | 3.83 | | | 0.01 | | | | 0.07 | | 0.10 | | 0.04 | |  |
| 3 | | 4 | | | | 53.31 | | | | | | | | | | 49.35 | | – | | **67.43** | | 59.00 | | 12.70 | | 25.04 | | | | 7.00 | | | 0.00 | | | | 0.01 | | 0.08 | | 0.05 | |  |
| 4 | | 4 | | | | 25.97 | | | | | | | | | | 15.72 | | 35.27 | | – | | **82.89** | | **60.59** | | **64.12** | | | | 43.11 | | | 1.03 | | | | 4.36 | | 7.43 | | 6.64 | |  |
| 5 | | 4 | | | | 15.51 | | | | | | | | | | 14.64 | | 27.86 | | **82.22** | | – | | **61.98** | | **72.57** | | | | 51.05 | | | 1.02 | | | | 4.50 | | 8.03 | | 7.59 | |  |
| 6 | | 4 | | | | 9.96 | | | | | | | | | | 2.49 | | 9.38 | | **91.15** | | **91.75** | | – | | **85.97** | | | | **71.66** | | | 1.46 | | | | 7.49 | | 13.44 | | 11.41 | |  |
| 7 | | 4 | | | | 5.62 | | | | | | | | | | 8.49 | | 12.63 | | **80.41** | | **84.79** | | **68.97** | | – | | | | **75.52** | | | 2.80 | | | | 12.09 | | 20.44 | | 18.14 | |  |
| 8 | | 4 | | | | 2.70 | | | | | | | | | | 3.30 | | 3.69 | | **75.17** | | **77.77** | | **67.48** | | **84.89** | | | | – | | | 6.97 | | | | 28.29 | | 41.37 | | 35.07 | |  |
| 9 | | 4 | | | | 0.95 | | | | | | | | | | 0.14 | | 0.24 | | 56.10 | | 53.62 | | 44.03 | | **71.41** | | | | **85.41** | | | – | | | | **83.36** | | **87.57** | | **83.21** | |  |
| 10 | | 4 | | | | 1.37 | | | | | | | | | | 0.24 | | 0.24 | | 57.71 | | 58.86 | | 47.71 | | **77.17** | | | | **87.95** | | | 37.75 | | | | – | | **86.45** | | **77.24** | |  |
| 11 | | 4 | | | | 1.42 | | | | | | | | | | 0.16 | | 0.23 | | 44.66 | | 46.66 | | 31.49 | | **64.73** | | | | **77.20** | | | 21.07 | | | | 56.06 | | – | | **84.42** | |  |
| 12 | | 4 | | | | 1.32 | | | | | | | | | | 0.09 | | 0.16 | | 34.84 | | 36.94 | | 21.31 | | 52.69 | | | | **64.97** | | | 11.46 | | | | 35.71 | | **75.32** | | – | |  |
| Subsections | | 1880s | | | | | | | | | | | | | | | | | | | | | | | | | | | | | | | | | | | | | | | | |  |
|  |  | *n* | | | 1 | | | | | | | 2 | | | | 3 | | 4 | | 5 | | 6 | | 7 | | 8 | | | | 9 | | | 10 | | 11 | | 12 | | 13 | | 14 | |  |
| 1 | | 10 | | | – | | | | | | | 58.91 | | | | 15.56 | | 0.01 | | 0.00 | | 0.00 | | 0.00 | | 0.01 | | | | 0.00 | | | 0.00 | | 0.00 | | 0.00 | | 0.00 | | 0.00 | |  |
| 2 | | 10 | | | 50.36 | | | | | | | – | | | | **63.80** | | 2.87 | | 0.40 | | 0.24 | | 0.19 | | 1.24 | | | | 0.02 | | | 0.01 | | 0.00 | | 0.00 | | 0.00 | | 0.00 | |  |
| 3 | | 10 | | | 8.17 | | | | | | | 53.89 | | | | – | | 49.22 | | 26.25 | | 21.28 | | 17.54 | | 36.90 | | | | 6.06 | | | 4.12 | | 1.13 | | 1.93 | | 0.80 | | 1.00 | |  |
| 4 | | 10 | | | 0.14 | | | | | | | 21.10 | | | | **91.77** | | – | | **62.58** | | 39.91 | | 26.69 | | **66.18** | | | | 1.28 | | | 0.48 | | 0.01 | | 0.03 | | 0.04 | | 0.05 | |  |
| 5 | | 10 | | | 0.02 | | | | | | | 5.02 | | | | **80.24** | | **72.74** | | – | | **87.31** | | **78.81** | | **93.53** | | | | 9.83 | | | 2.99 | | 0.05 | | 0.36 | | 0.13 | | 0.28 | |  |
| 6 | | 10 | | | 0.00 | | | | | | | 2.27 | | | | **69.52** | | 38.71 | | **79.72** | | – | | **93.30** | | **98.13** | | | | 34.12 | | | 11.43 | | 0.27 | | 0.96 | | 0.31 | | 0.43 | |  |
| 7 | | 10 | | | 0.01 | | | | | | | 1.10 | | | | 59.06 | | 17.26 | | 52.69 | | **81.93** | | – | | **96.88** | | | | 58.37 | | | 33.56 | | 3.99 | | 8.23 | | 2.72 | | 3.49 | |  |
| 8 | | 10 | | | 0.01 | | | | | | | 1.31 | | | | 48.63 | | 17.07 | | 36.90 | | 58.92 | | **73.43** | | – | | | | **60.68** | | | 43.86 | | 7.44 | | 12.18 | | 3.67 | | 4.88 | |  |
| 9 | | 10 | | | 0.00 | | | | | | | 0.23 | | | | 33.53 | | 1.33 | | 6.90 | | 36.25 | | **70.49** | | **98.03** | | | | – | | | **82.80** | | 9.48 | | 17.21 | | 2.64 | | 3.88 | |  |
| 10 | | 10 | | | 0.00 | | | | | | | 0.05 | | | | 25.58 | | 0.35 | | 1.52 | | 9.99 | | 42.43 | | **89.85** | | | | **74.57** | | | – | | 46.37 | | 53.23 | | 13.88 | | 17.23 | |  |
| 11 | | 10 | | | 0.00 | | | | | | | 0.00 | | | | 22.91 | | 0.02 | | 0.13 | | 1.88 | | 25.51 | | **77.80** | | | | 40.10 | | | **93.16** | | – | | **93.38** | | 40.12 | | 45.74 | |  |
| 12 | | 10 | | | 0.00 | | | | | | | 0.00 | | | | 20.10 | | 0.06 | | 0.42 | | 2.05 | | 24.83 | | 58.15 | | | | 23.45 | | | **69.96** | | **64.08** | | – | | **68.84** | | **75.41** | |  |
| 13 | | 5 | | | 0.00 | | | | | | | 0.00 | | | | 18.81 | | 0.09 | | 0.33 | | 1.41 | | 21.95 | | 49.62 | | | | 15.57 | | | **61.32** | | 54.18 | | **93.69** | | – | | **90.16** | |  |
| 14 | | 5 | | | 0.00 | | | | | | | 0.00 | | | | 16.23 | | 0.12 | | 0.51 | | 1.58 | | 21.89 | | 46.46 | | | | 14.41 | | | 54.62 | | 44.94 | | **91.47** | | **79.45** | | – | |  |
| Subsections | |  | | | | | | | |  | | | 1890s | | | | | | | | | | | | | | | | | | | | | | | | | | | | | | |
|  |  | *n* | | 1 | | | | 2 | | | 3 | | | | 4 | | | 5 | | 6 | | 7 | | 8 | | 9 | | 10 | | | 11 | | 12 | | 13 | | 14 | | 15 | | 16 | 17 | |
| 1 | | 8 | | – | | | | **87.40** | | | 32.85 | | | | 51.68 | | | 4.03 | | 4.98 | | 2.88 | | 6.14 | | 3.57 | | 3.05 | | | 2.01 | | 1.21 | | 2.32 | | 0.85 | | 0.37 | | 0.28 | 0.29 | |
| 2 | | 10 | | **88.55** | | | | – | | | 52.74 | | | | **62.89** | | | 11.02 | | 13.61 | | 7.85 | | 15.03 | | 9.48 | | 8.39 | | | 5.81 | | 3.43 | | 6.08 | | 3.08 | | 1.30 | | 0.91 | 1.23 | |
| 3 | | 10 | | **84.20** | | | | **93.28** | | | – | | | | **92.55** | | | 40.01 | | 44.55 | | 24.75 | | 46.52 | | 28.93 | | 22.36 | | | 12.66 | | 6.14 | | 15.25 | | 3.43 | | 0.54 | | 0.48 | 0.73 | |
| 4 | | 10 | | **67.10** | | | | **80.64** | | | **71.91** | | | | – | | | 41.35 | | 51.09 | | 38.01 | | 54.74 | | 44.45 | | 41.67 | | | 33.30 | | 23.43 | | 33.06 | | 15.91 | | 7.87 | | 6.12 | 6.48 | |
| 5 | | 10 | | 51.56 | | | | **83.48** | | | **77.22** | | | | **92.59** | | | – | | **94.89** | | **83.00** | | **93.83** | | **83.06** | | **76.80** | | | 59.84 | | 28.41 | | 55.25 | | 24.48 | | 3.53 | | 1.84 | 3.84 | |
| 6 | | 10 | | 42.42 | | | | **76.27** | | | **63.79** | | | | **90.79** | | | **84.12** | | – | | **86.28** | | **94.52** | | **87.15** | | **84.40** | | | **73.11** | | 49.28 | | 69.10 | | 38.11 | | 10.84 | | 5.95 | 8.65 | |
| 7 | | 10 | | 28.84 | | | | **60.43** | | | 42.90 | | | | **80.01** | | | **61.87** | | **85.89** | | – | | **97.02** | | **92.40** | | **92.27** | | | **88.20** | | **76.91** | | **87.26** | | **60.37** | | 31.23 | | 17.46 | 22.90 | |
| 8 | | 10 | | 26.28 | | | | 52.49 | | | 35.84 | | | | **76.26** | | | 46.63 | | **72.63** | | **81.53** | | – | | **87.25** | | **87.76** | | | **83.46** | | **74.58** | | **84.43** | | **60.64** | | 39.85 | | 28.38 | 31.72 | |
| 9 | | 10 | | 24.79 | | | | 51.92 | | | 33.22 | | | | **83.79** | | | 41.78 | | **74.29** | | **84.47** | | **96.28** | | – | | **93.88** | | | **91.04** | | **83.76** | | **90.49** | | **65.47** | | 43.88 | | 32.45 | 34.80 | |
| 10 | | 10 | | 20.09 | | | | 45.62 | | | 24.79 | | | | **77.95** | | | 33.09 | | **66.94** | | **79.33** | | **94.66** | | **91.53** | | – | | | **91.08** | | **85.15** | | **91.42** | | **70.13** | | 51.28 | | 38.22 | 40.90 | |
| 11 | | 10 | | 15.71 | | | | 40.15 | | | 16.28 | | | | **68.33** | | | 23.57 | | 57.85 | | **76.99** | | **94.08** | | **90.54** | | **93.04** | | | – | | **88.89** | | **94.56** | | **80.84** | | **65.24** | | 50.17 | 53.39 | |
| 12 | | 10 | | 13.05 | | | | 34.09 | | | 12.33 | | | | 59.68 | | | 13.60 | | 49.47 | | **74.14** | | **93.42** | | **87.68** | | **90.79** | | | **92.70** | | – | | **96.73** | | **83.75** | | **73.67** | | 59.73 | **62.52** | |
| 13 | | 10 | | 9.30 | | | | 25.25 | | | 8.75 | | | | 39.47 | | | 11.53 | | 32.22 | | 54.14 | | **80.09** | | **69.42** | | **73.81** | | | **79.32** | | **81.05** | | – | | **84.90** | | **77.01** | | **66.61** | **70.97** | |
| 14 | | 9 | | 5.13 | | | | 18.40 | | | 2.54 | | | | 24.20 | | | 5.20 | | 18.49 | | 40.81 | | **73.51** | | **60.20** | | **66.31** | | | **75.22** | | **75.54** | | **93.54** | | – | | **86.56** | | **77.50** | **83.41** | |
| 15 | | 10 | | 4.24 | | | | 14.99 | | | 1.01 | | | | 22.60 | | | 1.47 | | 11.14 | | 35.75 | | **75.03** | | **60.25** | | **67.28** | | | **78.13** | | **80.85** | | **95.55** | | **93.91** | | – | | **86.32** | **89.68** | |
| 16 | | 6 | | 3.79 | | | | 13.75 | | | 0.97 | | | | 23.29 | | | 0.73 | | 8.03 | | 25.17 | | **71.42** | | 56.90 | | **63.75** | | | **74.05** | | **76.98** | | **95.00** | | **92.88** | | **92.60** | | – | **91.23** | |
| 17 | | 6 | | 3.05 | | | | 11.96 | | | 0.85 | | | | 16.48 | | | 1.00 | | 6.92 | | 22.57 | | **61.78** | | 46.35 | | 53.02 | | | **64.98** | | **68.00** | | **92.76** | | **91.50** | | **88.65** | | **84.10** | – | |
| Subsections | | Contemporary | | | | | | | | | | | | | | | | | | | | | | | | | | | | | | | | | | | | | | |  |  |  |
|  |  | *n* | | | | 1 | | 2 | | | | | 3 | | | 4 | | 5 | | 6 | | 7 | | 8 | | 9 | | 10 | | | 11 | | 12 | | 13 | | 14 | | 15 | |  |  |  |
| 1 | | 7 | | | | – | | **78.43** | | | | | 55.57 | | | 37.86 | | 28.66 | | 14.90 | | 12.82 | | 14.25 | | 11.04 | | 13.30 | | | 7.91 | | 6.50 | | 7.49 | | 9.00 | | 7.28 | |  |  |  |
| 2 | | 7 | | | | **94.74** | | – | | | | | **76.46** | | | **60.14** | | 50.54 | | 30.00 | | 26.05 | | 27.98 | | 21.81 | | 25.52 | | | 15.68 | | 13.49 | | 14.32 | | 17.20 | | 13.01 | |  |  |  |
| 3 | | 7 | | | | **90.43** | | **89.15** | | | | | – | | | 83.96 | | 78.26 | | 58.30 | | 56.17 | | 57.95 | | 50.73 | | 53.94 | | | 39.97 | | 36.06 | | 37.44 | | 42.54 | | 35.29 | |  |  |  |
| 4 | | 7 | | | | **88.01** | | **87.88** | | | | | **93.68** | | | – | | **87.77** | | **71.77** | | **70.74** | | **72.33** | | **65.44** | | **68.00** | | | 54.34 | | 49.94 | | 50.97 | | 55.71 | | 47.49 | |  |  |  |
| 5 | | 7 | | | | **81.61** | | **83.55** | | | | | **92.04** | | | **89.93** | | – | | **78.92** | | **80.31** | | **82.31** | | **77.76** | | **80.04** | | | **68.87** | | **65.05** | | **65.55** | | **69.94** | | **62.16** | |  |  |  |
| 6 | | 7 | | | | **79.60** | | **83.02** | | | | | **94.16** | | | **93.30** | | **95.44** | | – | | **91.12** | | **93.01** | | **89.99** | | **91.25** | | | **80.57** | | **78.32** | | **79.87** | | **83.72** | | **76.60** | |  |  |  |
| 7 | | 7 | | | | **75.64** | | **78.81** | | | | | **92.14** | | | **90.35** | | **93.24** | | **86.65** | | – | | **93.06** | | **91.63** | | **92.97** | | | **84.70** | | **83.36** | | **84.63** | | **87.33** | | **81.74** | |  |  |  |
| 8 | | 7 | | | | **72.36** | | **74.32** | | | | | **87.66** | | | **85.46** | | **89.56** | | **81.69** | | **87.01** | | – | | **90.11** | | **91.98** | | | **83.24** | | **82.42** | | **84.94** | | **86.93** | | **82.53** | |  |  |  |
| 9 | | 7 | | | | **68.20** | | **70.04** | | | | | **85.29** | | | **82.76** | | **87.87** | | **79.40** | | **86.16** | | **90.59** | | – | | **92.80** | | | **85.54** | | **85.47** | | **88.23** | | **89.41** | | **86.72** | |  |  |  |
| 10 | | 6 | | | | **61.42** | | **63.31** | | | | | **79.03** | | | **76.18** | | **82.73** | | **73.76** | | **80.62** | | **86.12** | | **86.41** | | – | | | **83.33** | | **83.57** | | **86.28** | | **87.64** | | **85.13** | |  |  |  |
| 11 | | 5 | | | | 54.14 | | 57.35 | | | | | **75.33** | | | **73.10** | | **82.29** | | **72.00** | | **79.46** | | **85.36** | | **85.69** | | **90.02** | | | – | | **87.69** | | **89.06** | | **89.51** | | **88.02** | |  |  |  |
| 12 | | 6 | | | | 51.93 | | 54.90 | | | | | **73.42** | | | **70.75** | | **80.80** | | **71.83** | | **79.30** | | **85.67** | | **86.57** | | **91.05** | | | **88.87** | | – | | **91.21** | | **91.09** | | **90.27** | |  |  |  |
| 13 | | 6 | | | | 49.80 | | 51.58 | | | | | **69.89** | | | **67.19** | | **76.25** | | **66.54** | | **74.39** | | **81.59** | | **83.34** | | **88.35** | | | **84.54** | | **85.47** | | – | | **88.88** | | **88.55** | |  |  |  |
| 14 | | 6 | | | | 51.66 | | 53.86 | | | | | **71.95** | | | **96.60** | | **78.23** | | **68.96** | | **76.14** | | **82.99** | | **83.81** | | **89.19** | | | **85.37** | | **85.67** | | **88.95** | | – | | **88.49** | |  |  |  |
| 15 | | 5 | | | | 48.54 | | 49.10 | | | | | **66.87** | | | **64.08** | | **72.19** | | **62.74** | | **70.91** | | **79.20** | | **80.74** | | **95.98** | | | **81.40** | | **82.92** | | **87.25** | | **87.02** | | – | |  |  |  |

Fig. S1. Scheme of the upper beak subsections cut in *Gonatus fabricii* from inside the crest: subsections from the rostrum (anterior end) to the mid-point of the crest = 1 mm, and subsections from the mid-point of the crest towards its transparent part (posterior end) = 2 mm. Modified from Golikov *et al.* 2022, published under CC BY 4.0 (= no permission required to reuse).


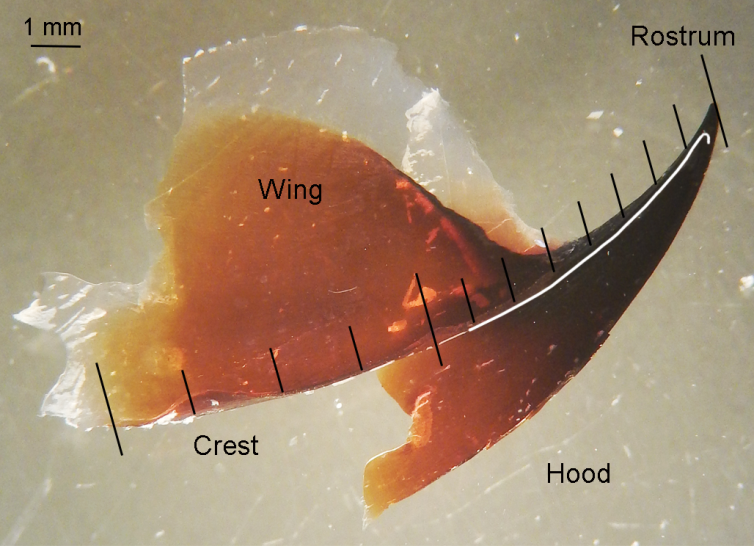


Fig. S2. Random two-dimensional elliptical projections of niche area of 95% of smaller (= beak subsection 1) and larger (= beak subsection 8) *Gonatus fabricii* (**a**–**d**) and *Todarodes sagittatus* (**e**, **f**) from the different time series. **a**, **c**. *Gonatus fabricii* with mantle length (ML) 6.6–7.5 mm (= beak subsection 1) from the Baffin Bay (**a**) and Nordic Seas (**c**). **b**, **d**. *Gonatus fabricii* ML 54.9–64.5 mm (= beak subsection 8) from the Baffin Bay (**b**) and Nordic Seas (**d**). **e**. *Todarodes sagittatus* ML 9.1–10.0 mm (= beak subsection 1). **h**. *Todarodes sagittatus* ML 72.7–80.4 mm (= beak subsection 8).


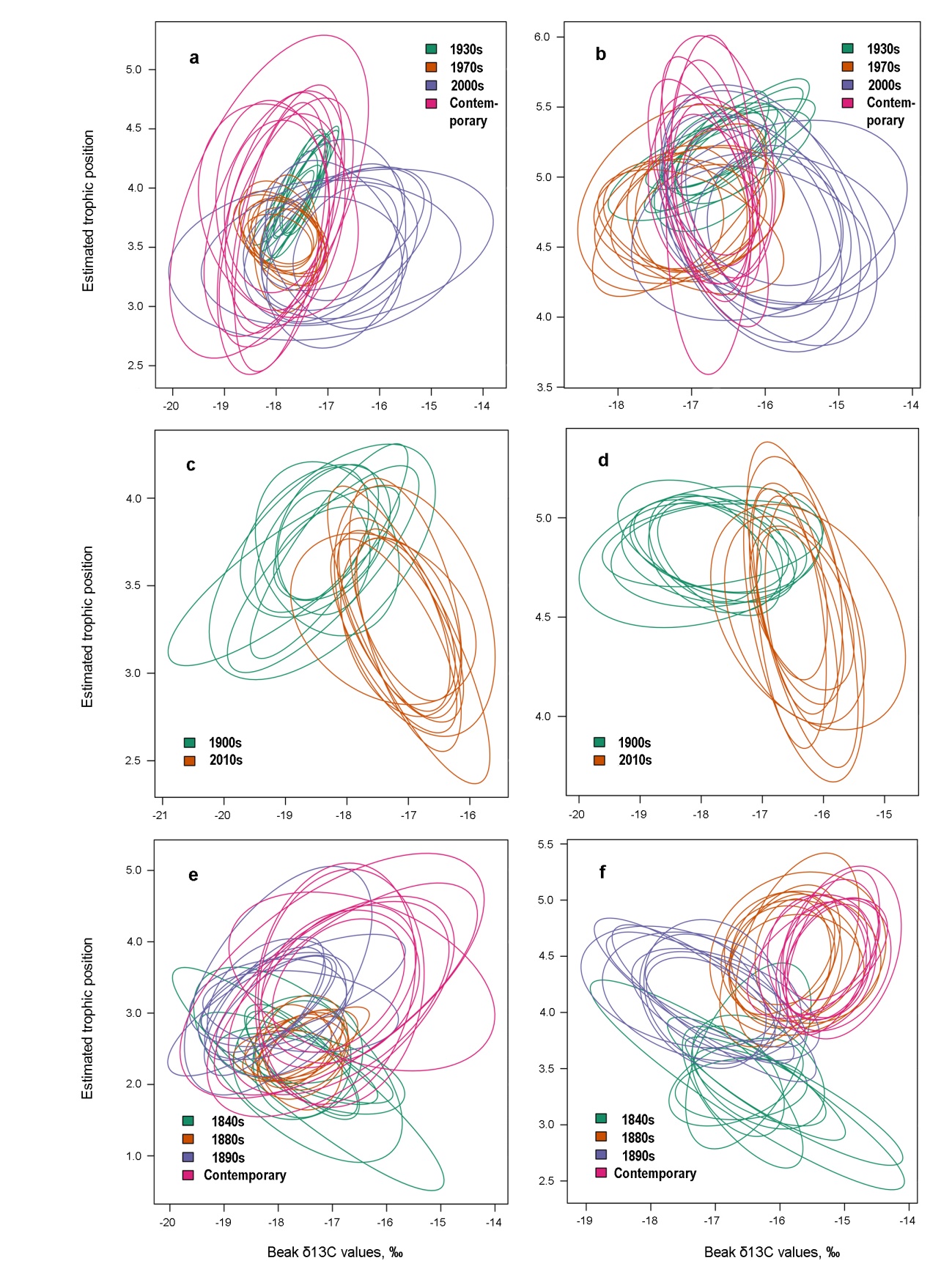


Fig. S3. Long-term trends in *δ*^13^C values (**a**, **b**), estimated trophic position (**c**, **d**) and specialization index (**e**, **f**) of *Todarodes sagittatus* between 1844 and 2023. **a**, **c**. Squid with mantle length (ML) 9.1–10.0 mm (= beak subsection 1). **b**, **d**. Squid ML 72.7–80.4 mm (= beak subsection 8). **e**. Specialization index of *δ*^13^C, entire beaks. **f**. Specialization index of trophic position, entire beaks. Blue line represents generalized additive model and grey area represents confidence intervals. All individual data points are shown.


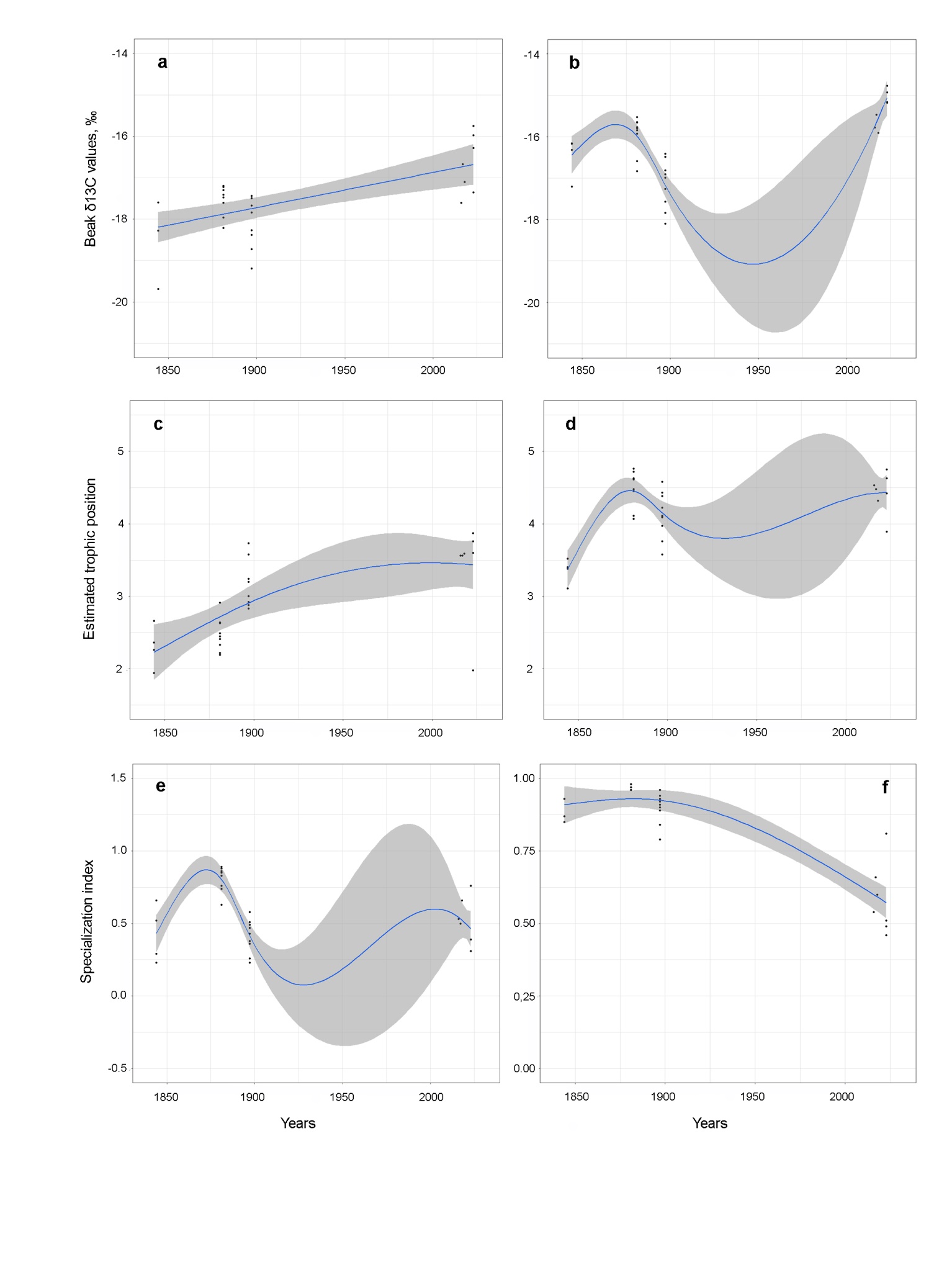


Fig. S4. Ontogenetic trends in *δ*^13^C values of *Gonatus fabricii* with mantle length 6.6–64.5 mm (= beak subsections 1 to 8) as generalized additive mixed effect models (**a**–**g**) and graph (**h**). **a**. 1890s. **b**. 1900s. **c**. 1930s. **d**. 1970s. **e**. 2000s. **f**. 2010s. **g**. Contemporary (2016, 2017 and 2019). **h**. Graph with all the time series, where violet = 1890s, black = 1900s, blue = 1930s, yellow = 1970s, magenta = 2000s, brown = 2010s and turquoise = contemporary time series. Blue line represents model of values and colour dots represent factor for individual number, and grey area represents confidence intervals. Mean values and standard error are shown on the graph.


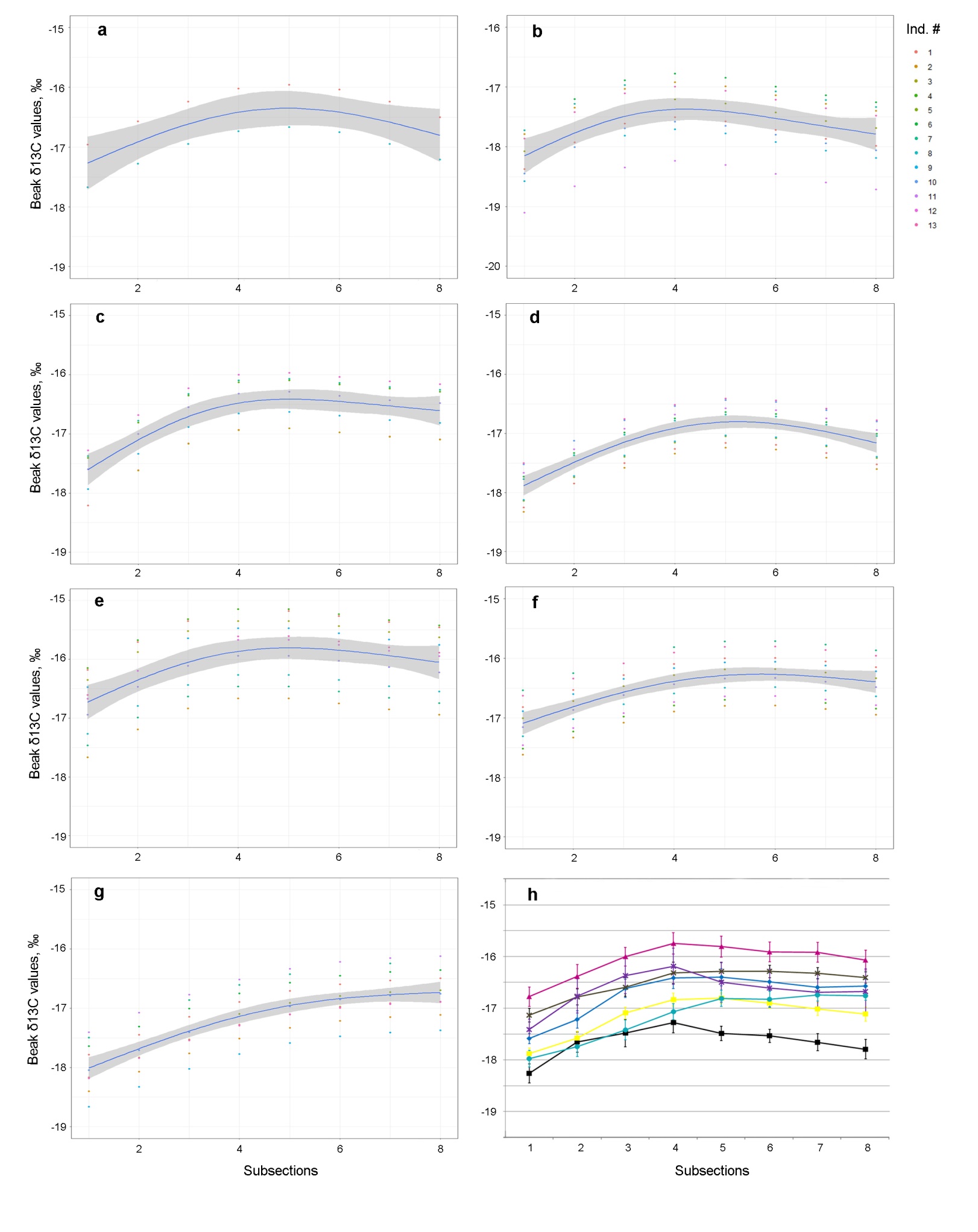


Fig. S5. Ontogenetic trends in estimated trophic position of *Gonatus fabricii* with mantle length 6.6–64.5 mm (= beak subsections 1 to 8) as generalized additive mixed effect models (**a**–**g**) and graph (**h**). **a**. 1890s. **b**. 1900s. **c**. 1930s. **d**. 1970s. **e**. 2000s. **f**. 2010s. **g**. Contemporary (2016, 2017 and 2019). **h**. Graph with all the time series, where violet = 1890s, black = 1900s, blue = 1930s, yellow = 1970s, magenta = 2000s, brown = 2010s and turquoise = contemporary time series. Blue line represents model of values and colour dots represent factor for individual number, and grey area represents confidence intervals. Mean values and standard error are shown on the graph.


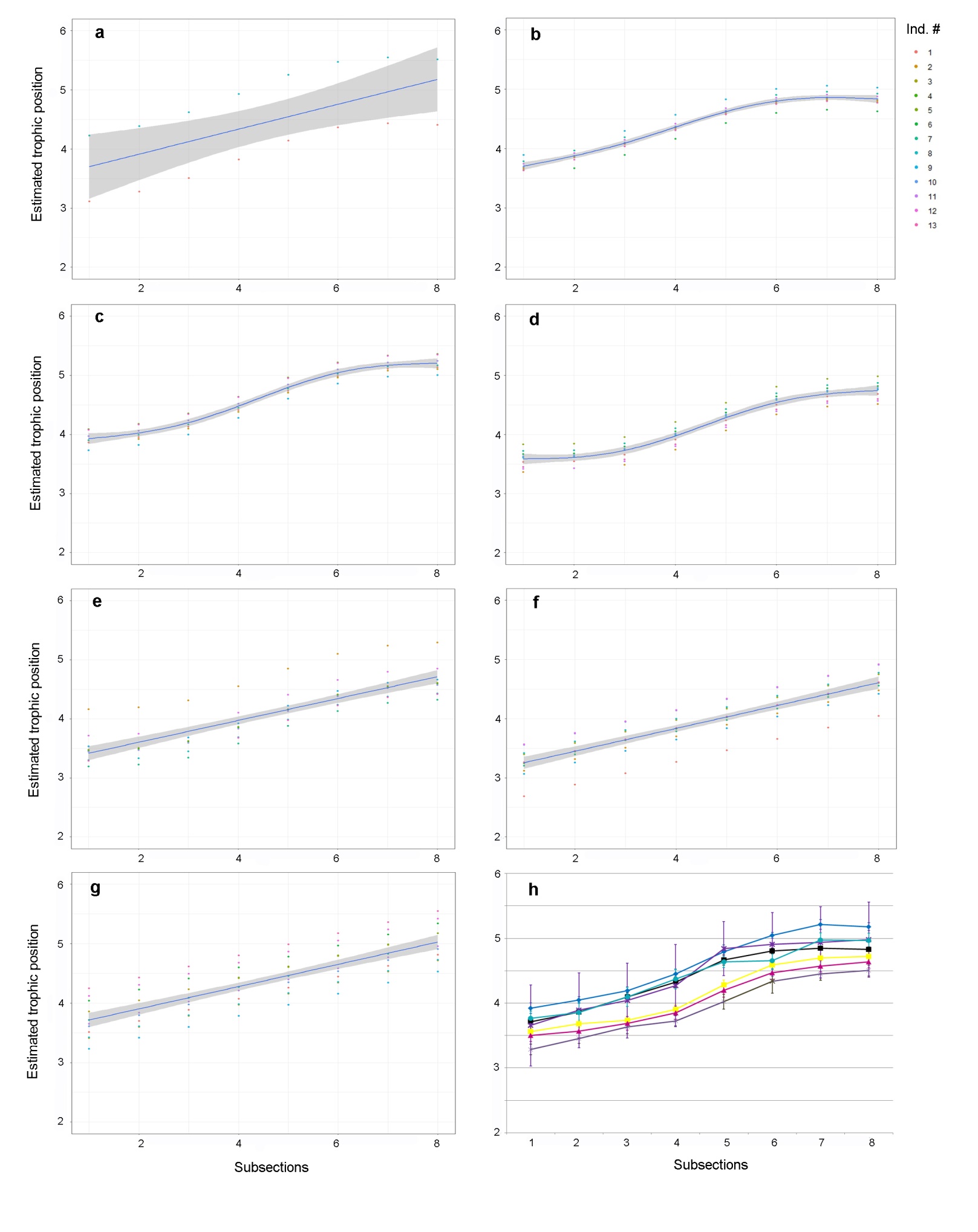


Fig. S6. Random two-dimensional elliptical projections of niche area of 95% of each beak subsection within a given time series of *Gonatus fabricii*. **a**. 1900s. **b**. 1930s. **c**. 1970s. **d**. 2000s. **e**. 2010s. **f**. Contemporary (2016, 2017 and 2019).


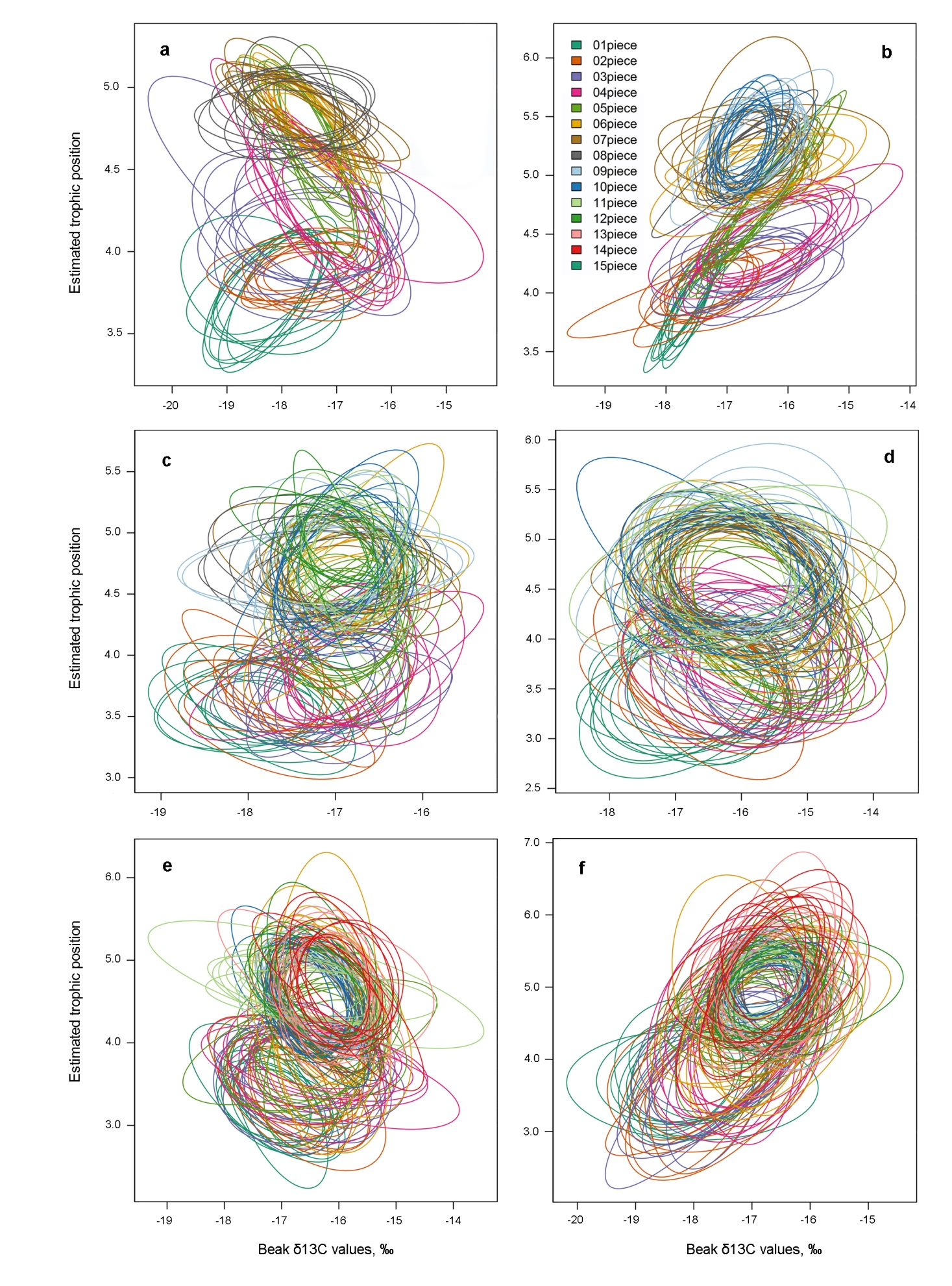


Fig. S7. Random two-dimensional elliptical projections of niche area of 95% of each beak subsection within a 1970s time series of *Gonatus fabricii*, exemplifying the four periods found in niche changes history based on overlap patterns.


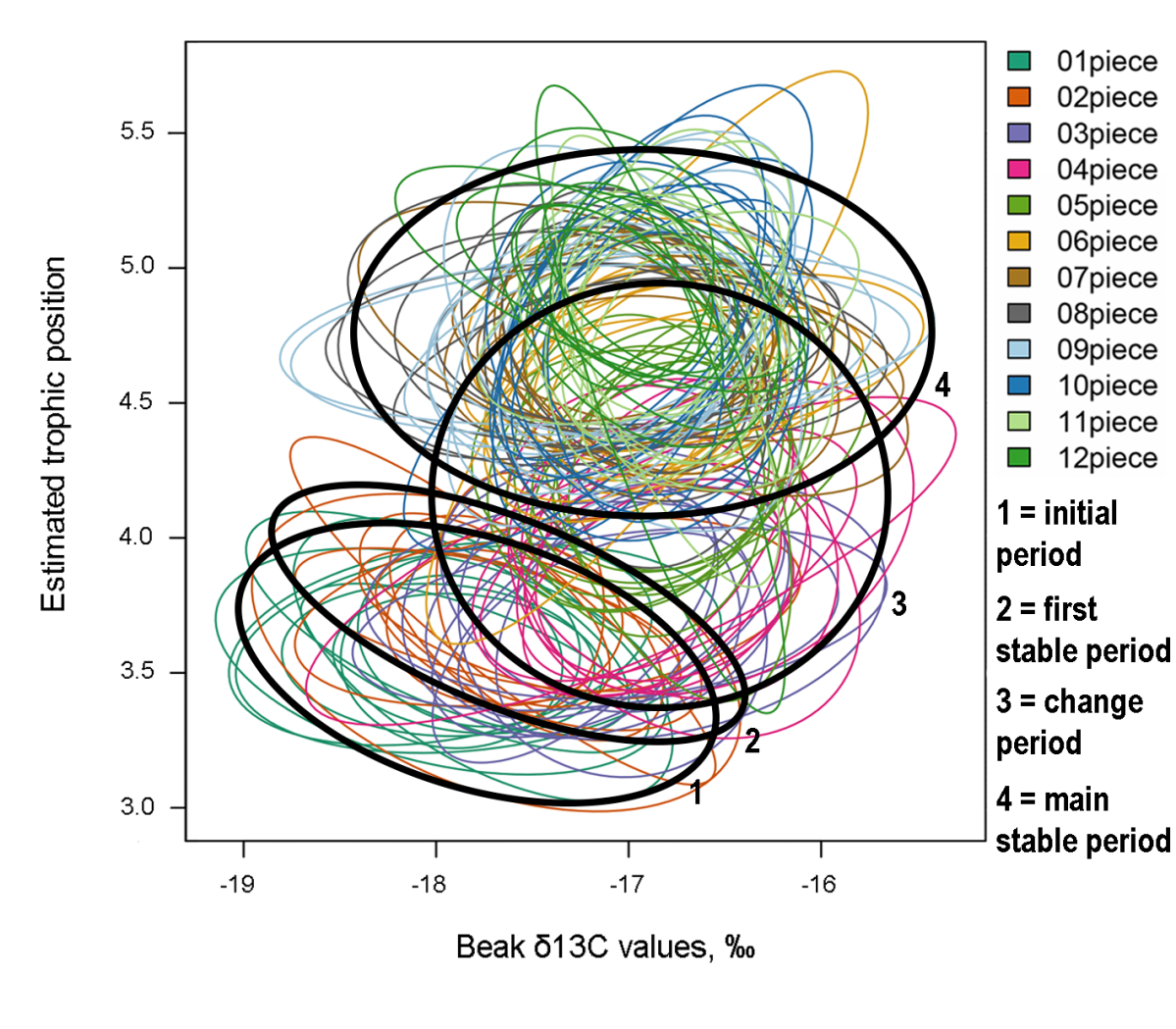


Fig. S8. Ontogenetic trends in *δ*^13^C values and estimated trophic position (TP) of *Todarodes sagittatus* with mantle length 9.1–80.4 mm (= beak subsections 1 to 8) as generalized additive mixed effect models (**a**–**h**) and graph (**i**, **j**). **a**, **e**. 1840s, *δ*^13^C values (**a**) and TP (**e**). **b**, **f**. 1880s, *δ*^13^C values (**b**) and TP (**f**). **c**, **g**. 1890s, *δ*^13^C values (**c**) and TP (**g**). **d**, **h**. Contemporary (2016–2018 and 2023), *δ*^13^C values (**d**) and TP (**h**). **i**, **j**. Graphs for *δ*^13^C values (**i**) and TP (**j**) with all the time series, where blue = 1840s, magenta = 1880s, black = 1890s and turquoise = contemporary time series. Blue line represents model of values and colour dots represent factor for individual number, and grey area represents confidence intervals. Mean values and standard error are shown on the graph.


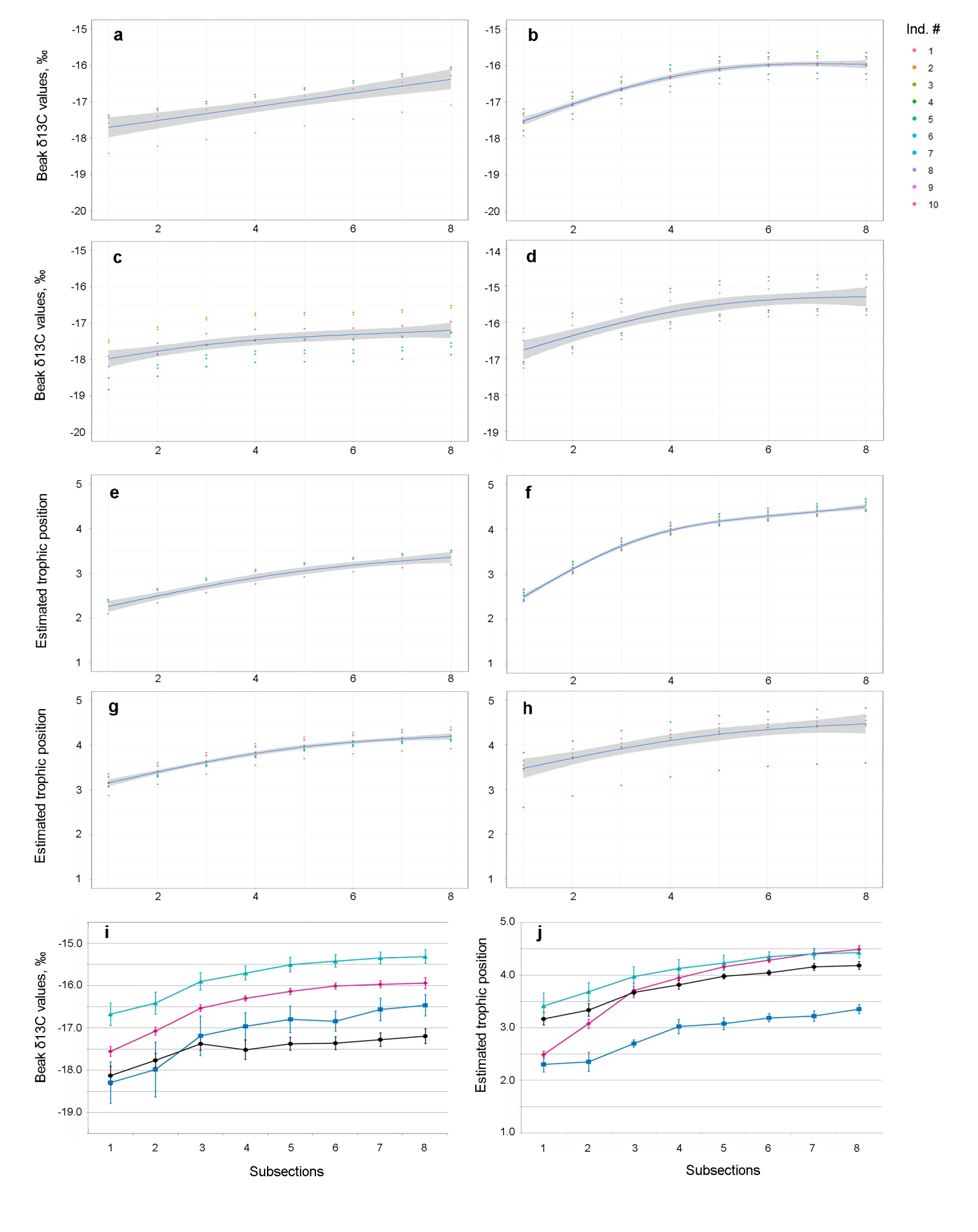


Fig. S9. Random two-dimensional elliptical projections of niche area of 95% of each beak subsection within a given time series of *Todarodes sagittatus*. **a**. 1840s. **b**. 1880s. **c**. 1890s. **d**. Contemporary (2016–2018 and 2023).


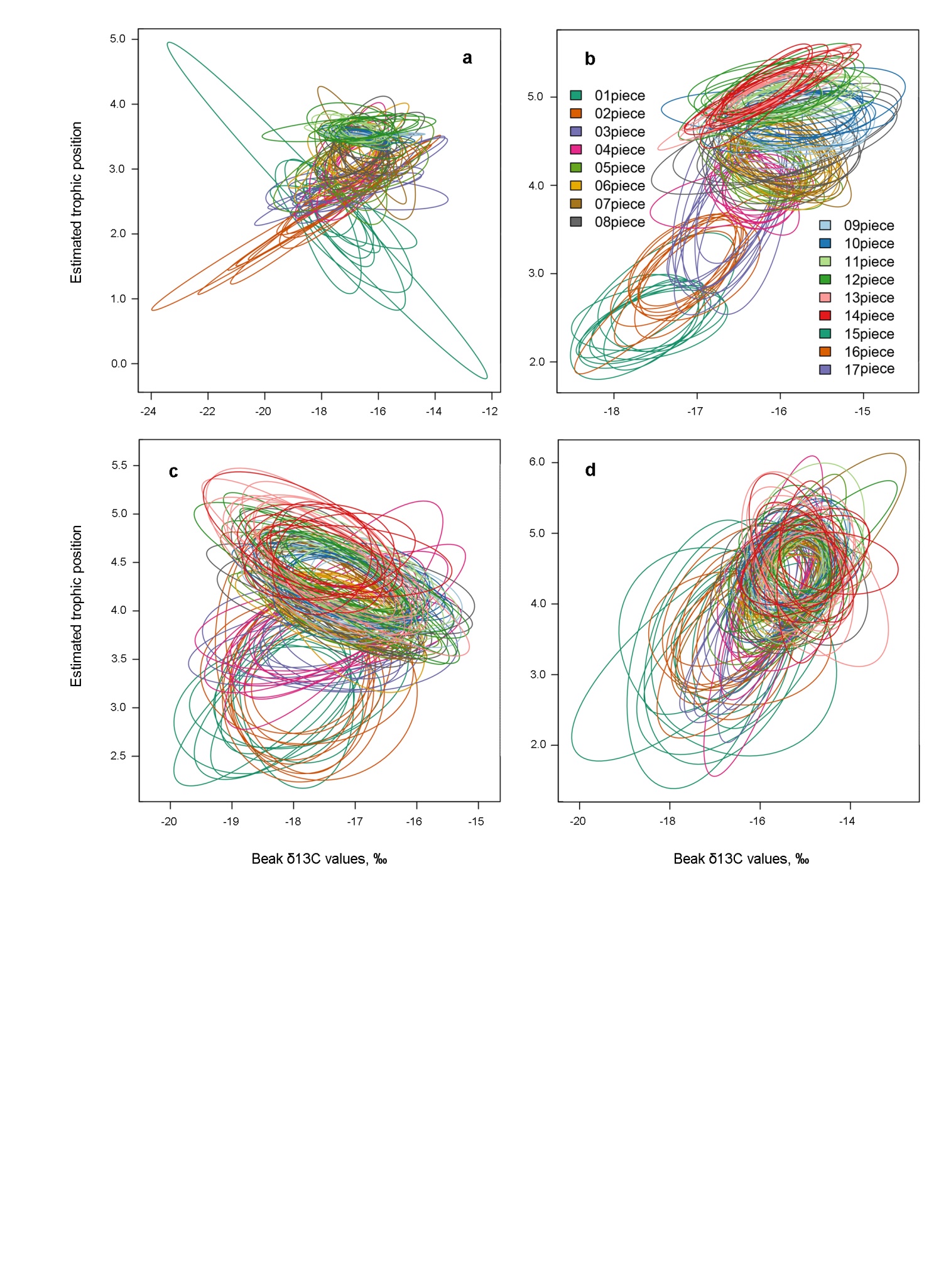


**References**

Aubail, A. *et al.* Temporal trend of mercury in polar bears (*Ursus maritimus*) from Svalbard using teeth as a biomonitoring tissue. J. Environ. Monit. 14, 56–63 (2012).

Aubail, A., Dietz, R., Rigét, F., Simon-Bouhet, B. & Caurant, F. An evaluation of teeth of ringed seals (*Phoca hispida*) from Greenland as a matrix to monitor spatial and temporal trends of mercury and stable isotopes. Sci. Total Environ. 408, 5137–5146 (2010).

Dietz, R. *et al.* Analysis of narwhal tusks reveals lifelong feeding ecology and mercury exposure. Curr. Biol. 31, 2012–2019 (2021).

Dietz, R. *et al.* Temporal trends and future predictions of mercury concentrations in northwest Greenland polar bear (*Ursus maritimus*) hair. Environ. Sci. Technol. 45, 1458–1465 (2011).

Golikov, A. V. *et al.* Ontogenetic changes in stable isotope (*δ*^13^C and *δ*^15^N) values in squid *Gonatus fabricii* (Cephalopoda) reveal its important ecological role in the Arctic. *Mar. Ecol. Prog. Ser.* **606**, 65–78 (2018)

Golikov, A. V. *et al.* Life history of the arctic squid *Gonatus fabricii* (Cephalopoda: Oegopsida) reconstructed by analysis of individual ontogenetic stable isotopic trajectories. Animals 12, 3548 (2022).

Matthews, C. J. D. & Ferguson, S. H. Validation of dentine deposition rates in beluga whales by interspecies cross dating of temporal d13C trends in teeth. NAMMCO Sci. Publ. 10, 1–19 (2014).

Yurkowski, D. J., Hussey, N. E., Ferguson, S. H. & Fisk, A. T. A temporal shift in trophic diversity among a predator assemblage in a warming Arctic. R. Soc. Open Sci. 5, 180259 (2018).

Zhao, S., Davoren, G. K., Matthews, C. J. D., Ferguson, S. H. & Watt, C. A. Isotopic (*δ*^15^N and *δ*^13^C) profiles in dentine indicate sex differences and individual variability in resource use among narwhals (*Monodon monoceros*). Mar. Mammal Sci. 38, 1182–1199 (2022).
